# From Dead Ends to Catalyst Reservoirs: Bimetallic Reactivation of Homoleptic Nickel Pincer Complexes

**DOI:** 10.1021/acs.inorgchem.6c01379

**Published:** 2026-06-02

**Authors:** Jun-Yang Ye, Zheng-Feng Zhang, Matthias Zeller, Ming-Der Su, Wei-Tsung Lee

**Affiliations:** † Department of Chemistry, 34911 National Central University, Taoyuan 32001, Taiwan; ‡ Department of Applied Chemistry, 63107 National Chiayi University, Chiayi 60004, Taiwan; § Department of Chemistry, 8522 Purdue University, West Lafayette, Indiana 47907, United States; ∥ Department of Medicinal and Applied Chemistry, Kaohsiung Medical University, Kaohsiung 80708, Taiwan

## Abstract

Homoleptic nickel­(II) pincer complexes are commonly regarded as thermodynamically stable off-cycle species that terminate catalytic activity. Here we show that such complexes can undergo clean conversion to monohalide LNiBr species through a bimetallic reactivation pathway. Treatment of a homoleptic Ni­(II) pincer complex with NiBr_2_ generates a transient binuclear intermediate formed by association of the external metal salt with the pincer complex. UV–vis monitoring reveals the accumulation and subsequent decay of this intermediate during the transformation. Time-dependent DFT calculations identify characteristic metal–metal charge-transfer (MMCT) transitions that provide an electronic signature for the binuclear species and support the proposed structure. Notably, simple halide salts such as LiBr also promote conversion of the homoleptic complex to the monohalide LNiBr complex. These results provide insight into the reactivity of homoleptic nickel pincer complexes and illustrate how transient bimetallic interactions can mediate their transformation.

## Graphical abstract

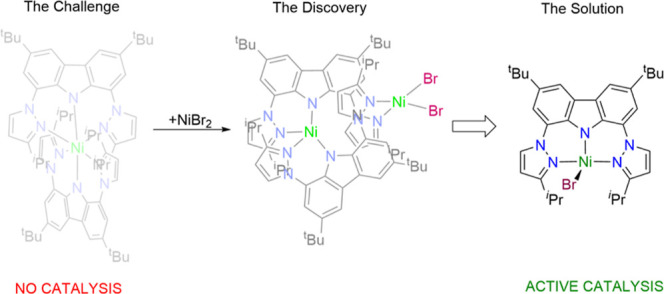

## Introduction

Transition metal complexes have long been at the forefront of catalysis, playing an important role in enabling various chemical transformations critical to both industrial processes and academic research. From the Haber-Bosch process for ammonia synthesis to modern cross-coupling reactions such as the Suzuki–Miyaura and Heck reactions, transition metal catalysts have revolutionized synthetic chemistry by offering high activity, selectivity, and tunability.^1–3^ Nickel-based catalysts, in particular, have garnered significant attention due to their earth-abundance, cost-effectiveness, and ability to facilitate reactions traditionally dominated by more expensive metals like palladium.^4,5^ For instance, nickel complexes bearing pincer ligands, which provide a robust tridentate coordination environment, have been extensively explored for their ability to catalyze C–C and C–X bond-forming reactions, as well as reductions and oxidations, owing to their well-defined coordination geometry and electronic properties.^6–13^

Despite widespread utility, a persistent challenge in transition metal catalysis is the deactivation of catalysts, which can lead to the premature shutdown of reactions and limit their practical applicability. Catalyst deactivation can occur through various pathways, including ligand dissociation, metal aggregation, or the formation of stable, catalytically inactive species.^14–16^ Among these, the formation of homoleptic metal complexes, such as L_n_M (L = multidentate ligand; M = metal; *n* = 2–3) (Chart 1), represents a particularly well-known and challenging deactivation route.^17,18^ For example, in nickel catalysis, the formation of bis-ligated species like L_2_Ni (where L is a pincer ligand) is often viewed as a thermodynamic sink or “dead-end” due to their lack of vacant coordination sites or electronic flexibility required for catalytic turnover.^19,20^ This stability renders them inert to further reactivity, effectively halting the catalytic process and posing a significant barrier to the development of efficient and sustainable catalytic systems.

**Chart 1:**
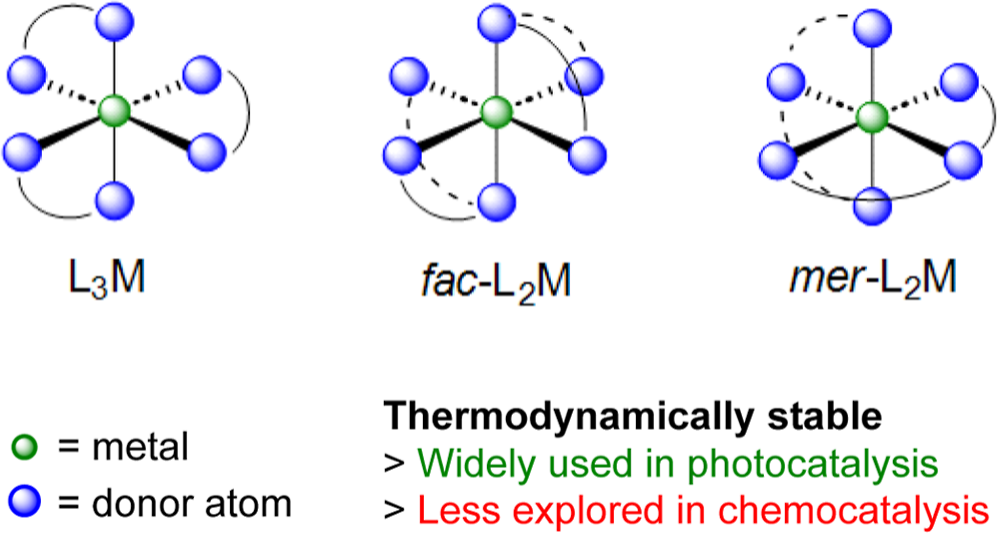
Structures of homoleptic metal complexes.

However, it is worth noting that fully coordinated homoleptic complexes with multidentate ligands are widely recognized for their utility in photocatalysis, where robust ligand frameworks protect metal centers and could facilitate photochemical processes.^21–23^ In contrast, their application as chemocatalysts remains largely underexplored, as detailed mechanistic insights into their catalytic behavior are scarce.^24,25^ For example, Paul and co-workers reported the use of a homoleptic nickel complex for catalytic alcohol oxidation (Scheme 1a), yet explicitly noted that such species are suboptimal for catalysis.^26^ Additionally, Zhou et al. utilized a homoleptic nickel complex in a Suzuki cross-coupling reaction (Scheme 1b). While they provided a mechanistic discussion for related heteroleptic nickel species, no mechanistic explanation was offered for the homoleptic system, despite the report of moderate product yields.^27^ Consequently, the assumed inertness of homoleptic complexes has discouraged efforts to explore their potential for in situ activation or catalytic turnover. Overcoming this perceived limitation by developing strategies to convert these otherwise dormant species back into catalytically active forms would represent a paradigm shift in catalyst design and utilization. Such an approach could significantly enhance catalyst longevity, enable recycling, and reduce catalyst loadings, thereby improving the economic and environmental sustainability of catalytic processes.^28^

**Scheme 1:**
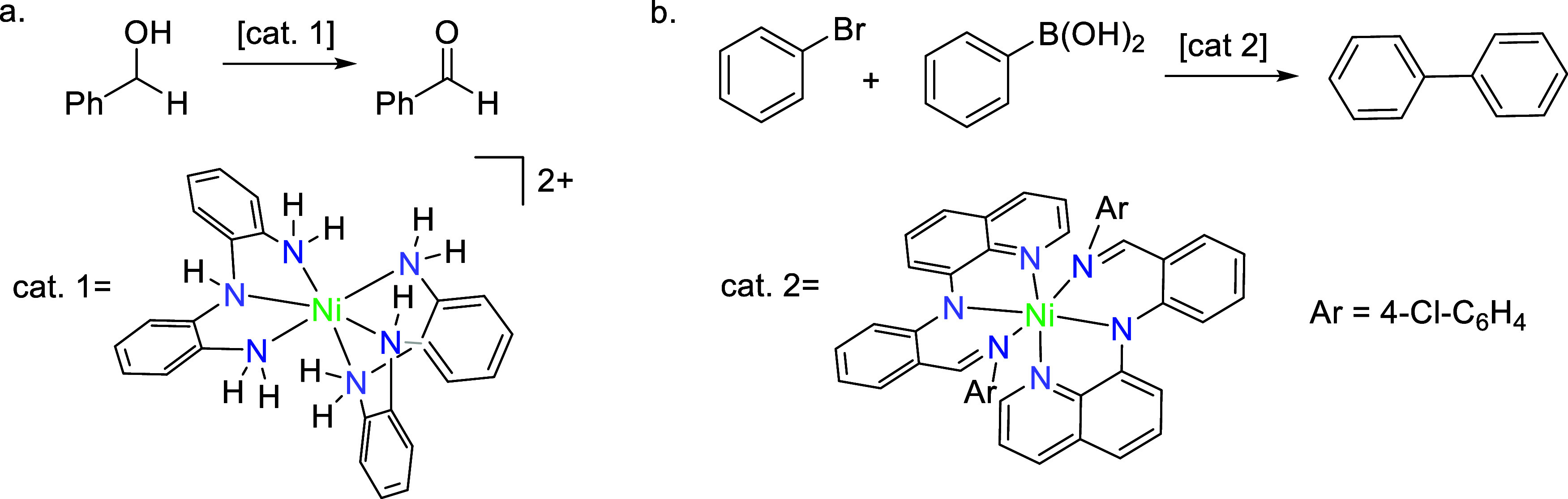
Selected Examples of Reactions Catalyzed by Homoleptic Complexes: (a) Alcohol Oxidation Reported by Paul and Co-Workers, and (b) Suzuki Cross-Coupling Reaction Reported by Zhou et al

This study reports a distinct finding that challenges the conventional perception of homoleptic complexes as catalytically inert. We demonstrate that a nickel homoleptic complex with NNN pincer ligands, L_2_Ni, is converted into the active LNiBr species upon treatment with NiBr_2_, transforming a stable but inactive form into a catalytic precursor. Additionally, the addition of simple alkali salts, such as LiBr, enhances the release of LNiBr, enabling its effective catalysis in subsequent reactions. This approach offers a method to overcome catalyst deactivation, utilizing inexpensive reagents to regenerate active species. Our results provide insights into the reactivity of homoleptic complexes and support the development of more sustainable catalytic systems catalytic systems.

## Results and Discussion

### Discovery of Thermodynamically Reversible Homoleptic Nickel Species

Our initial objective was to synthesize and characterize two analogues, derived by modifying the known nickel complex Cz^*t*Bu^(Pyr^*i*Pr^)_2_NiBr with less bulky substituents around the nickel center. This approach followed established protocols for the synthesis of Cz^*t*Bu^(Pyr^*i*Pr^)_2_MX (M = Fe, Co, Ni; X = Cl, Br, I) (Scheme 2a),^29–31^ with the anticipation that reducing steric bulk would promote a more planar coordination geometry (larger θ, Scheme 2b), thereby enabling better control over the reactivity of the complexes. The synthesis of Cz^*t*Bu^(Pyr^Me^)_2_NiBr (crystal structure shown in Figure 1) proceeded smoothly following literature procedures. In contrast, during the synthesis and reaction optimization of Cz^*t*Bu^(Pyr^H^)_2_NiBr (**1**) (crystal structure shown in Figure 1), we encountered a series of unexpected observations, which ultimately led to the serendipitous discovery of a distinct reactivity pattern.

**Scheme 2:**
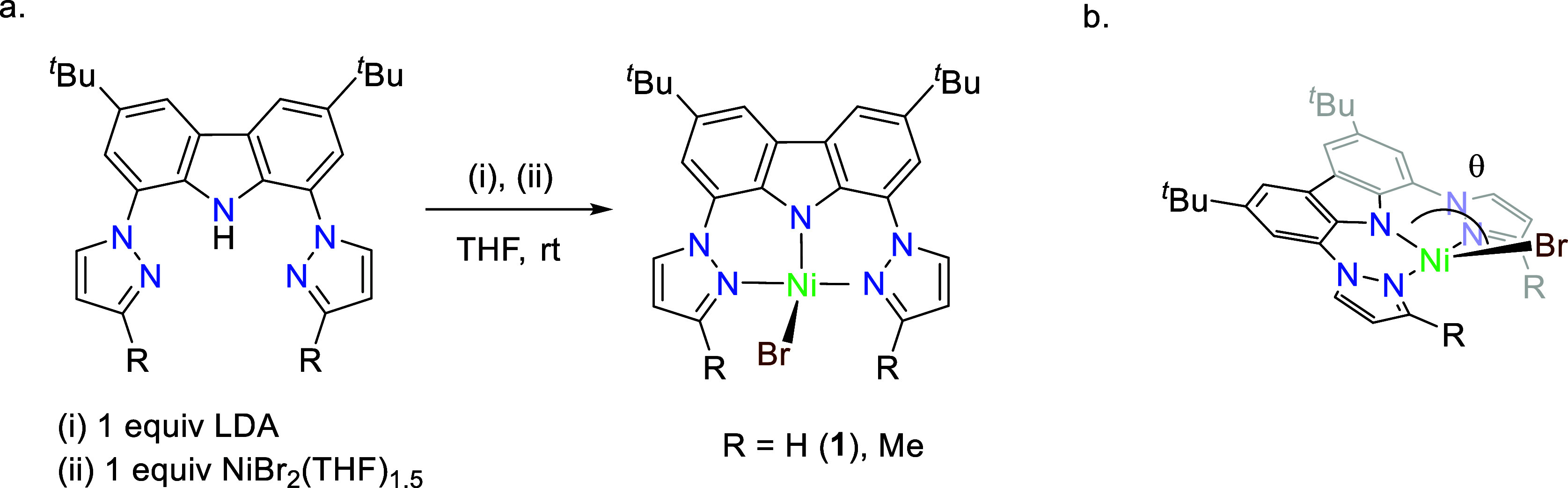
(a) Synthesis of **1** and Cz^*t*Bu^(Pyr^Me^)_2_NiBr, and (b) Geometric Representation of the Coordination Site Showing the Planarization Angle θ

**Figure 1:**
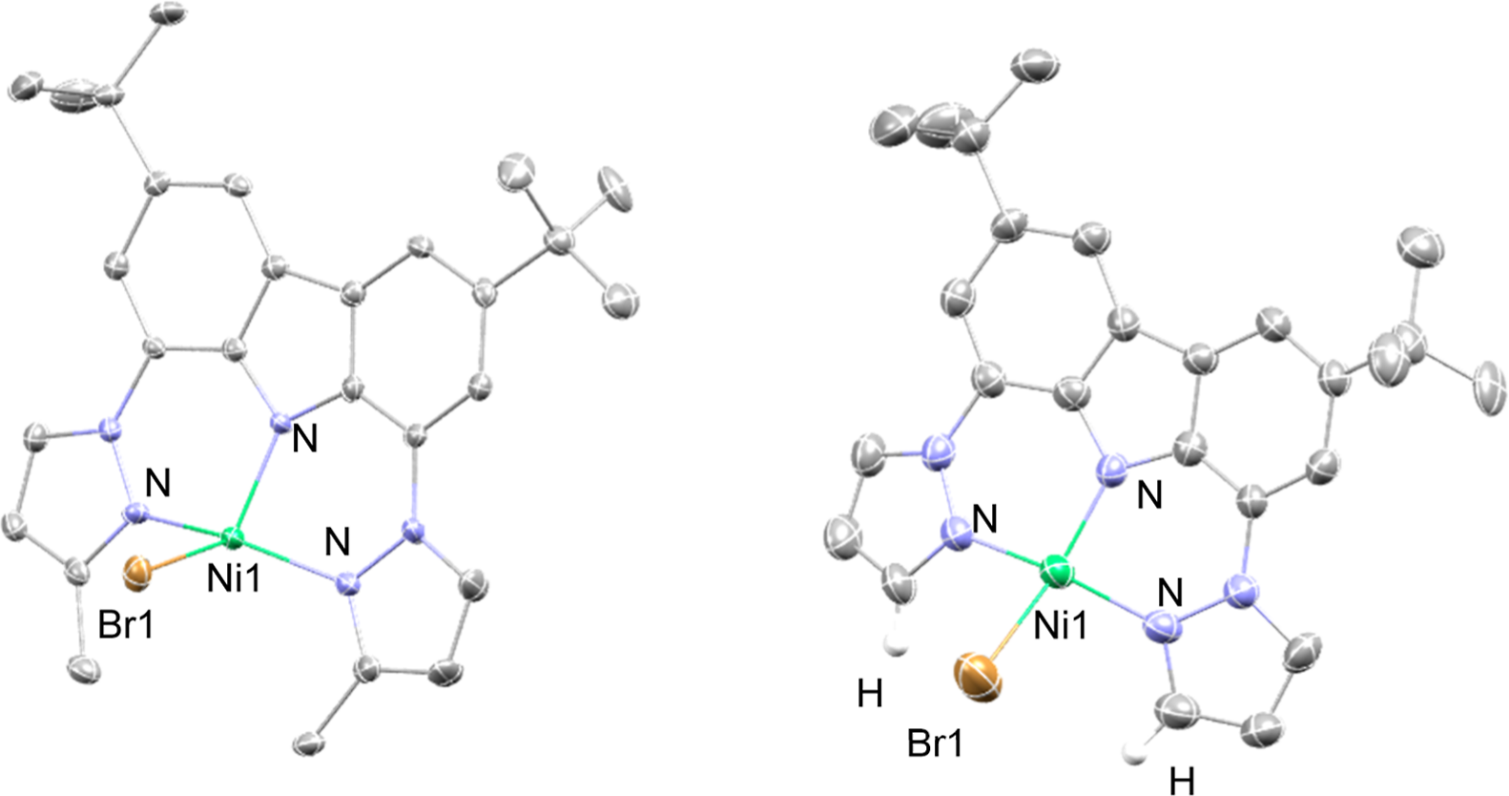
Molecular structure of Cz^*t*Bu^(Pyr^Me^)_2_NiBr (left) and **1** (right) with thermal ellipsoids at the 50% probability level. Noncoordinated solvent molecules and most hydrogen atoms are omitted for clarity. Color key: turquoise = Ni, blue = N, gray = C, brown = Br, white = H.

Analysis of the targeted **1** by ^1^H NMR in THF-*d*_8_ revealed paramagnetic resonances, featuring a major set of signals for a homoleptic octahedral Ni­(II) complex, [(Cz^*t*Bu^(Pyr^H^)_2_)_2_Ni] (**2**) (Figure S1), which is generally observed for minimal steric hindrance ligand profiles, and several unidentified paramagnetic species. However, surprisingly, when the reaction mixture was heated to 80 °C (Scheme 3), the near-complete disappearance of **2** and the emergence of the previously minor unidentified paramagnetic signals as the dominant species. Single-crystal X-ray diffraction analysis confirmed the unknown species as **1**(**THF**)_2_ (Figure S2), a THF-solvated 6-coordinate octahedral Ni­(II) complex, where the reversible THF coordination has been observed in prior studies.^27^ This finding indicated that **2** can be converted to **1** in the presence of NiBr_2_, highlighting a potential ligand exchange rendering a reactivated pathway.

**Scheme 3:**
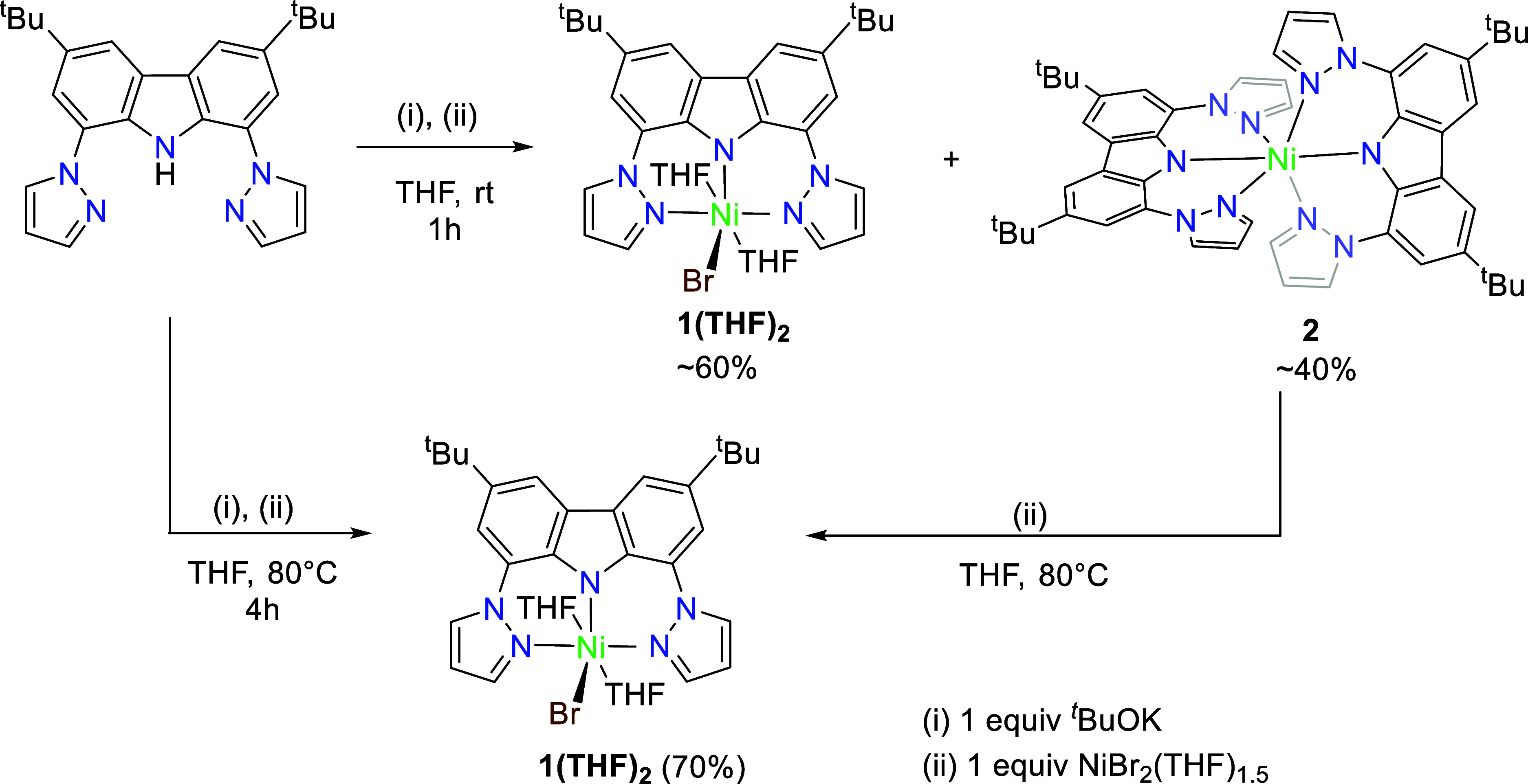
Synthesis of **1**(**THF**)_2_ and Its Reversible Interconversion With Homoleptic Complex **2**

To further elucidate this conversion, analogue homoleptic complex [(Cz^*t*Bu^(Pyr^Me^)_2_)_2_Ni] (**3**) and [(Cz^*t*Bu^(Pyr^*i*Pr^)_2_)_2_Ni] (**4**) featuring increased steric hindrance compared to **2**, were synthesized and structurally characterized (Figure S3). Comparative analysis of the crystal structures of these three complexes reveals a steric profile that correlates with the substituent bulkiness in the order ^*i*^Pr > Me > H. For instance, the average Ni–N bond lengths and torsion angles of the carbazolide planes indicate increasing steric crowding within the coordination sphere, following the trend **4** > **3** > **2**, as quantified in Table 1 and Figure S4. Compared to the structurally related analogue, [(Dpa^Me^(Pyr^H^)_2_)_2_Ni],^32^ complex **2** exhibits a more idealized octahedral geometry (τ_6_ = 0.08 vs 0.10).^33^ This is reflected in a shorter Ni–N_central_ bond and elongated Ni–N_pyr_ distances. These differences could be attributed to the more rigid and planar carbazolide backbone, which enforces stronger interaction with the nickel center relative to the more flexible diphenylamide framework. For **3** and **4**, the pyrazole–pyrazole distances (3.0–3.2 Å) are consistent with potential weak π–π interactions between adjacent rings, although their influence on the observed structural parameters cannot be conclusively established.

**Table 1:** Selected Bond Distances (Å) and Angles (°) for **2**–**4**

	**2**	**3**	**4**
Ni1–N3	2.140(2)	2.219(1)	2.241(3)
Ni1–N5	2.119(2)	2.254(1)	2.285(3)
Ni1–N8	2.140(2)	2.214(1)	2.203(3)
Ni1–N10	2.150(2)	2.220(1)	2.349(3)
∠C1–C2–N2–N3	19.0(3)	22.5(2)	20.0(5)
∠C12–C11–N4–N5	4.2(3)	30.4(2)	33.0(5)
∠C27–C28–N7–N8	16.1(3)	18.6(2)	15.1(6)
∠C38–C37–N9–N10	24.7(3)	21.7(2)	30.1(5)
Avg Ni–N	2.137	2.226	2.269
Avg∠C–C–N–N	16.0	23.3	24.5

### Spectroscopic Monitoring of Homoleptic Nickel Reactivation

To probe the reaction kinetics, UV–Vis spectroscopy was used to monitor the conversion process. However, the limited solubility of NiBr_2_ in the employed solvents prevented achieving sufficiently high reactant concentrations, which in turn restricted the determination of activation parameters and confined the study to relative conversion rates. Although the transformation proceeds more rapidly in THF (Figures S5 and S6), the reactions were monitored in toluene because it provides a distinct absorption band at ∼640 nm, characteristic of the four-coordinate seesaw geometry in the Cz^*t*Bu^(Pyr^R^)_2_NiBr complexes.^29^ At 80 °C, complete conversion of **3** and **4** to Cz^*t*Bu^(Pyr^Me^)_2_NiBr and Cz^*t*Bu^(Pyr^*i*Pr^)_2_NiBr required 240 and 90 min (Figure 2), respectively, whereas complex **2** exhibited no detectable reaction under identical conditions (Figure S7). Though reliable monitoring of this transformation was feasible only in MeCN due to the overlapping UV–Vis absorbance features of complex **2** and **1**(**THF**)_2_ in THF (Figure S8).^34^ The observed reaction rates in toluene correlate with the degree of steric crowding within the coordination sphere, as a more twisted coordination environment results in elongated Ni–N bond lengths, facilitating pyrazole ligand dissociation. The liberated pyrazole can subsequently coordinate to NiBr_2_, ultimately promoting the formation of Cz^*t*Bu^(Pyr^R^)_2_NiBr.

**Figure 2:**
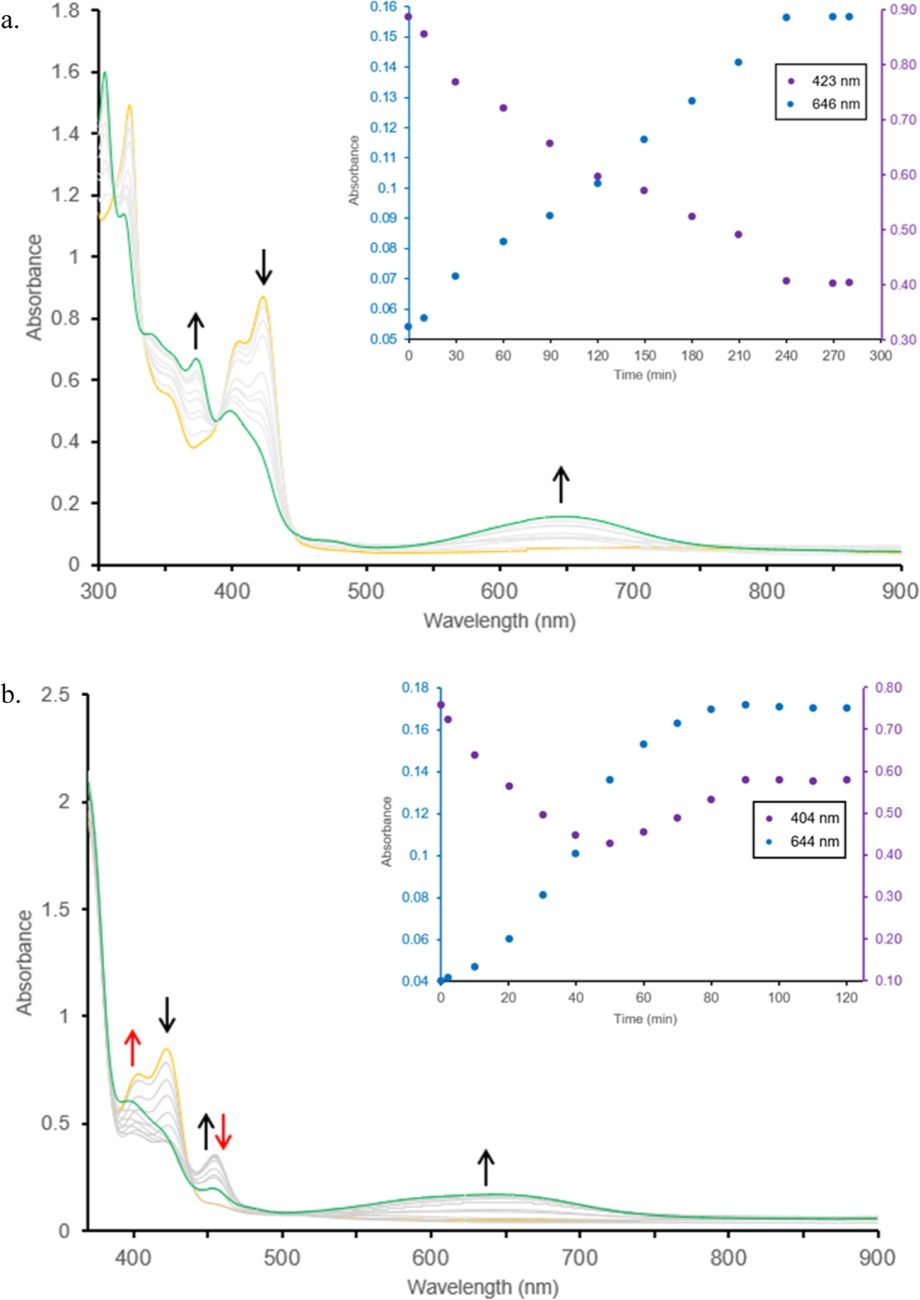
UV–Vis spectra showing the conversion of (a) complex **3** to Cz^*t*Bu^(Pyr^Me^)_2_NiBr and (b) complex **4** to Cz^*t*Bu^(Pyr^*i*Pr^)_2_NiBr upon addition of NiBr_2_ in toluene at 80 °C. Inset: Absorbance vs time plot.

Particularly, UV–Vis monitoring indicates the formation of an intermediate species during the conversion of complex **4** to Cz^*t*Bu^(Pyr^*i*Pr^)_2_NiBr, as evidenced by an unusual absorbance–time profile indicative of a sequential two-step pathway (Scheme 4) for complex **4** (Figure 2b), whereas the presence of isosbestic points in the reactions of complexes **2** (Figure S9) and **3** (Figure 2a) were observed. The conversion of complex **4** to Cz^*t*Bu^(Pyr^*i*Pr^)_2_NiBr is likely driven by the steric repulsion between the two bulky isopropyl groups within the coordination sphere, facilitating the dissociation of pyrazole ligands. This two-step reaction pathway was further elucidated using UV–Vis spectroscopy (Figures S10 and S11). The first step, spanning approximately 50 min, was marked by the emergence of absorption bands at approximately 456 and 640 nm, accompanied by the diminution of a band at 424 nm, indicative of the formation of intermediate species, with a concomitant color change from yellow to red. The second step initiated after 50 min, characterized by the diminution of the 424 nm band, the development of a new growing absorption feature at 400 nm, and a slight blue shift of the 650 nm band, suggesting a structural reorganization leading to the formation of Cz^*t*Bu^(Pyr^*i*Pr^)_2_NiBr, accompanied by a color change from red to green and isosbestic points in this phase.

**Scheme 4:**
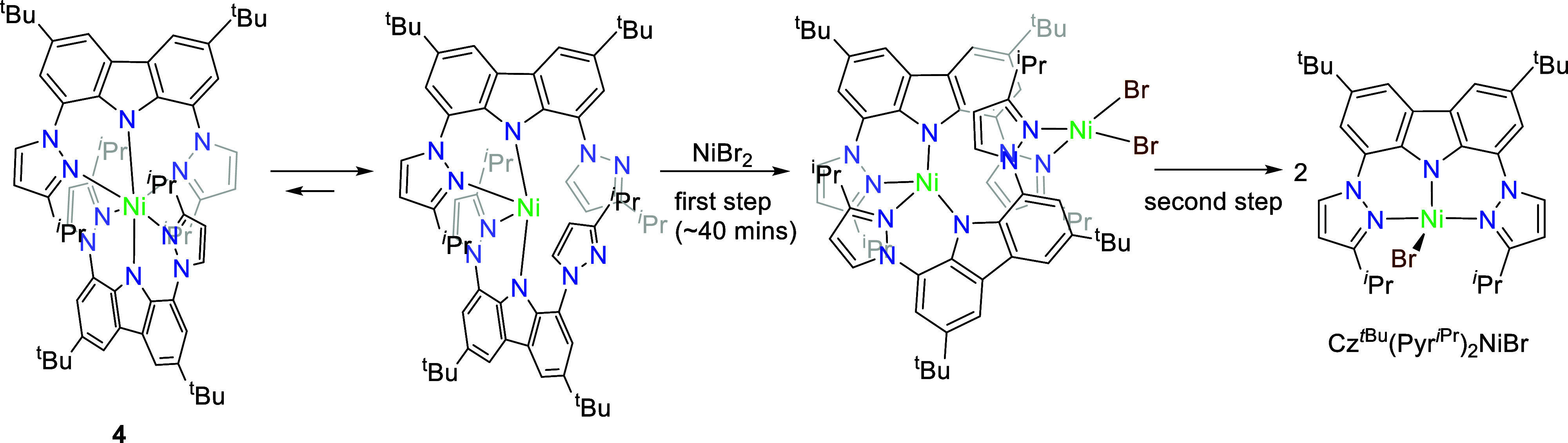
Proposed Bimetallic Pathway for Reactivation of Homoleptic Nickel Pincer Complexes

The conversion of complex **4** to Cz^*t*Bu^(Pyr^*i*Pr^)_2_NiBr was further investigated using ^1^H NMR spectroscopy, with the reaction conducted in C_6_D_6_ at 80 °C over 24 h (Figure 3). The spectra revealed a gradual transformation of **4** into Cz^*t*Bu^(Pyr^*i*Pr^)_2_NiBr, characterized by the absence of additional peaks that could be assigned to the proposed dinuclear intermediate, contrasting with the intermediate species observed via UV–Vis monitoring. This discrepancy may be attributed to the higher concentration employed in the NMR experiments, which could favor a direct ligand exchange.

**Figure 3:**
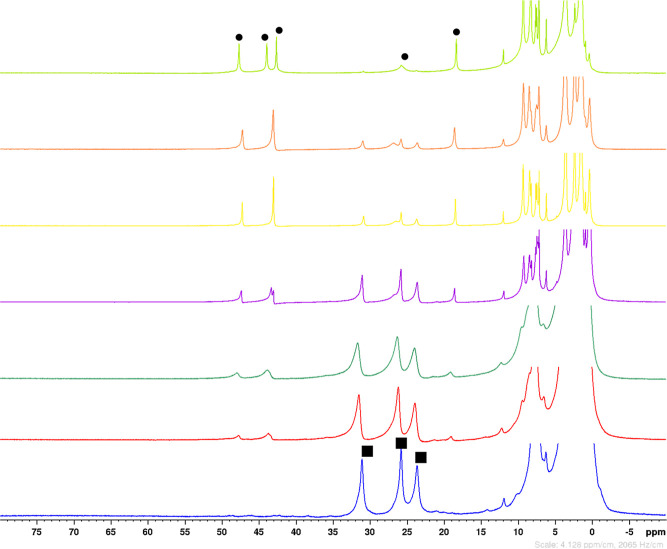
^1^H NMR spectroscopic monitoring of the conversion of **4** to Cz^*t*Bu^(Pyr^*i*Pr^)_2_NiBr upon addition of NiBr_2_. Overlay of ^1^H NMR spectra (500 MHz, in C_6_D_6_, at 80 °C) recorded at different time points. Key signals corresponding to reactant **4** (■) and product Cz^*t*Bu^(Pyr^*i*Pr^)_2_NiBr (•) are indicated. Each spectrum represents a time point of 0 h (−), 1 h (−), 4 h (−), 8 h (−), 14 h (−), 20 h (−), 24 h (−).

Despite numerous attempts, the synthesis of a dinuclear nickel complex analogue to the spectroscopically observed intermediate was unsuccessful, even though a dicobalt analogue had been successfully synthesized and characterized in previous studies.^31^ The only well-defined nickel species isolated from these efforts was a heteronuclear nickel–silver complex, which could be crystallized but exhibited quite limited stability in solution. X-ray crystallography (Figure 4) revealed a distorted tetrahedral geometry at the nickel center (τ_4_ = 0.77). This structural assignment supports the tetrahedral geometry proposed for the spectroscopic intermediate. Moreover, the blue shift observed in the UV–Vis spectra (Figure S12) during conversion to the final product is consistent with an increase in ligand-to-metal charge transfer (LMCT) transition energy, as the coordination environment changes from a tetrahedral to a seesaw geometry.^29^

**Figure 4:**
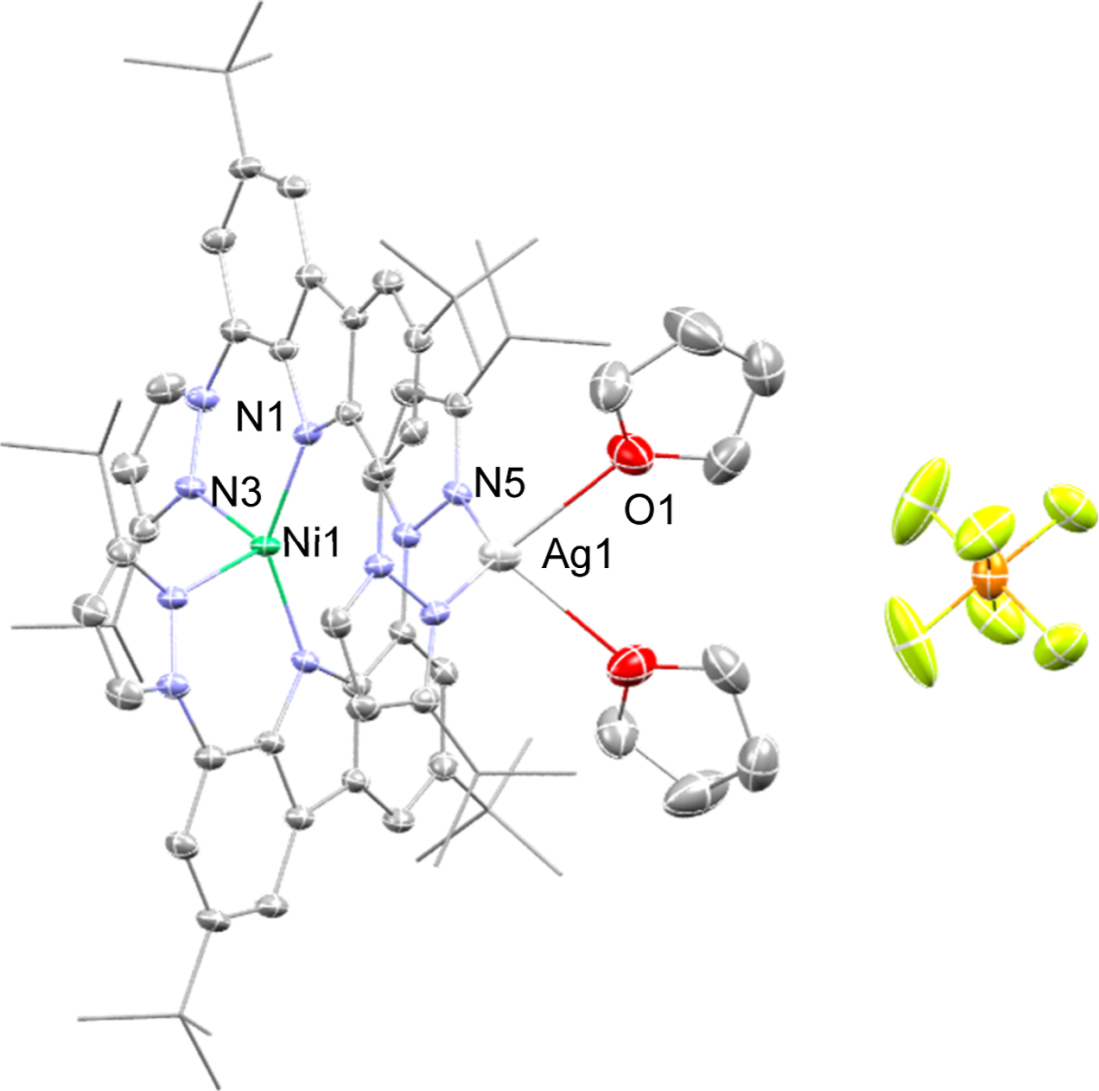
Molecular structure of (Cz^*t*Bu^(Pyr^*i*Pr^)_2_)_2_Ni–Ag–PF_6_ with thermal ellipsoids at the 50% probability level. Noncoordinated solvent molecules, disorder and hydrogen atoms are omitted for clarity. Color key: turquoise = Ni, white = Ag, orange = P, yellow-green = F, red = O, blue = N, gray = C.

### DFT Mechanistic Insights into Nickel Pincer Reactivation

To elucidate the reactivation pathway, density functional theory (DFT) calculations were performed for both **3** and **4** at the UM06L-D3/def2-SVP level, consistent with the triplet ground state of these Ni­(II) complexes (Table S3). The optimized structures reveal pronounced asymmetry in the Ni–N coordination sphere, with four shorter equatorial Ni–N bonds (avg. ∼1.9 Å) and two elongated axial interactions (avg. ∼2.2 Å), indicative of partial hemilability of the pyrazolyl donors. This structural feature provides a plausible entry point for ligand dissociation upon treatment with NiBr_2_. The computed free–energy profiles (Figure 5) support a common stepwise mechanism for both complexes. Initial partial dissociation of a pyrazolyl arm enables association with NiBr_2_ via the first transition state (TS1) to form a binuclear intermediate. Subsequent Ni–N bond cleavage proceeds through a four-membered rhomboidal [Ni­(μ-N)­(μ-Br)­Ni] transition state (TS2), ultimately affording the monohalide species. In both systems, a well-defined intermediate minimum is located between TS1 and TS2, consistent with a sequential transformation rather than a concerted process.

**Figure 5:**
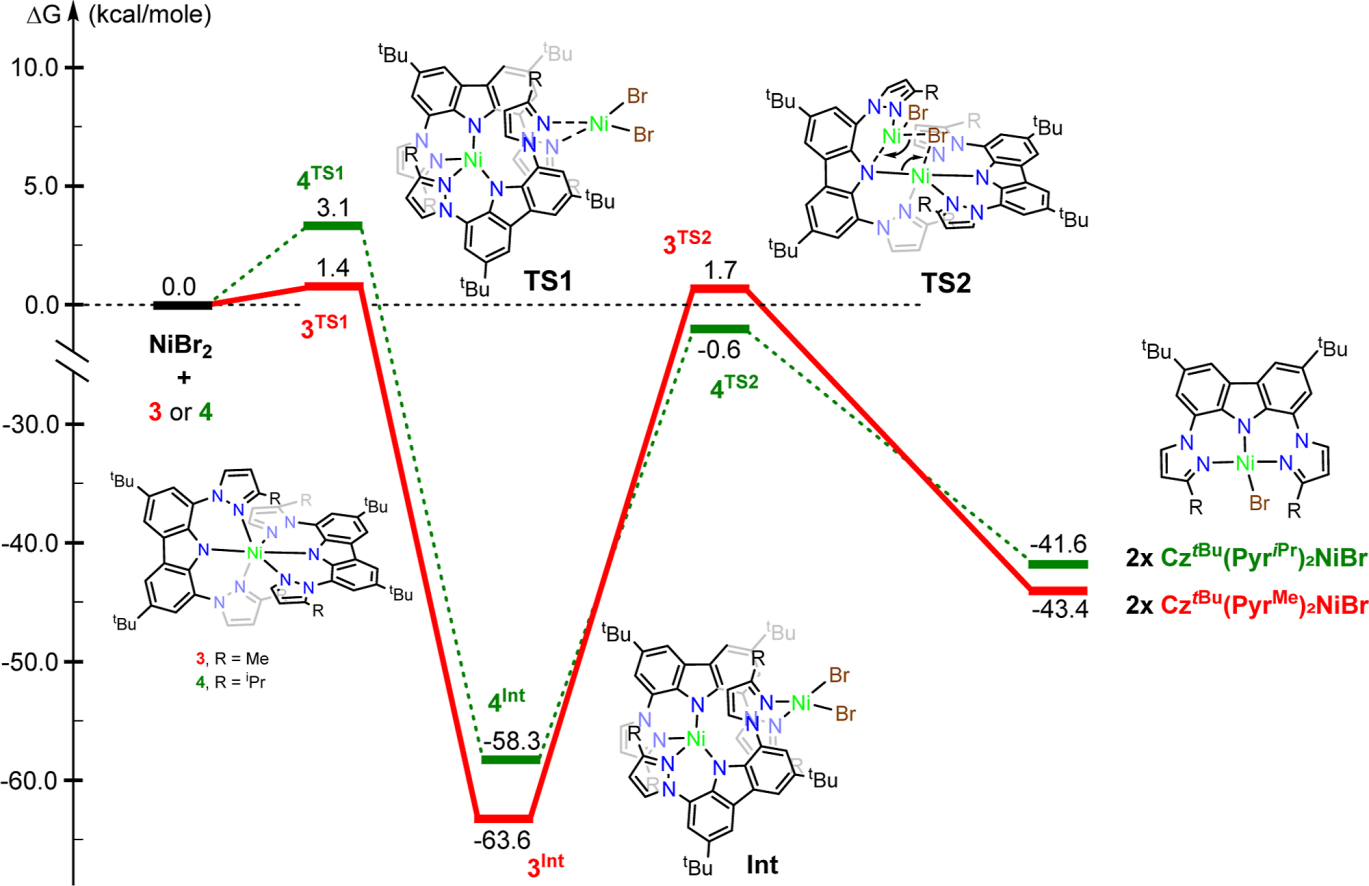
Free energy coordinate for the conversion of **3** and **4** into monohalide active catalysts through reaction with NiBr_2_ (UM06L-D3/def2-svp).

The two-stage absorbance evolution observed during the reaction of **4** with NiBr_2_ was further elucidated by time-dependent DFT (TD-DFT) calculations. The computed electronic spectrum of the intermediate (**4** ^**Int**^) displays a low-energy transition at approximately 540 nm. While this reflects a systematic bathochromic shift common in TD-DFT treatments of Ni­(II) systems, the appearance of an isolated visible–region transition is in qualitative agreement with the experimentally observed band, and the relative spectral pattern is well reproduced.

Analysis of the excited-state configurations indicates that the visible absorption originates from two related transitions (Tables S3 and S4, Figures S19–S24). The dominant excitation (T_3_, f = 0.065) is characterized primarily by β-HOMO–2 to LUMO promotion and possesses substantial LMCT character involving the pincer framework and the nickel center. A second transition (T_4_) contains a significant contribution (63%) from HOMO–3 to LUMO+1 excitation, corresponding to charge transfer from orbitals localized on the exogenous NiBr_2_ fragment into the acceptor manifold of the primary nickel core. This metal-to-metal charge-transfer (MMCT) component is diagnostic of electronic communication between the two nickel centers. The combined LMCT/MMCT character of these excitations is therefore consistent with a structurally and electronically coupled binuclear intermediate.

Notably, calculations indicate that the corresponding methyl-substituted intermediate (**3** ^**Int**^) is thermodynamically comparable, and slightly lower in free energy, relative to **4** ^**Int**^. Nevertheless, no distinct intermediate is resolved experimentally for **3** under identical monitoring conditions. Given the similar computed electronic structures of **3** ^**Int**^ and **4** ^**Int**^, this disparity does not suggest a change in mechanistic pathway. Rather, it likely reflects subtle differences in steric environment that influence the effective population and persistence of the binuclear intermediate in solution. The more sterically encumbered isopropyl substituents in **4** may favor a more well-defined coordination geometry, facilitating accumulation of a spectroscopically distinguishable species. It should be noted that the computed pathway is intended to provide qualitative mechanistic insight consistent with the experimental observations. Given the complexity of the system, including potential solvent coordination and metal–metal interactions, alternative pathways cannot be excluded.

### Hydrodehalogenation Catalysis by Reactivated Nickel Pincer Complexes

To demonstrate that homoleptic nickel complexes can be converted into catalytically active species, we selected a well-studied nickel complex,^35–39^ [(^Me^N_2_N)­NiBr] (Chart 2),^40^ along with its homoleptic analogue, [(^Me^N_2_N)_2_Ni] (Chart 2), for evaluation of both their interconversion and catalytic performance. The transformation was carried out in C_6_D_6_ in the presence of 1 equiv of NiBr_2_ at 80 °C, leading to a clean and complete conversion of [(^Me^N_2_N)_2_Ni] to [(^Me^N_2_N)­NiBr] within 3 h, as confirmed by ^1^H NMR spectroscopy (Figure S13). Subsequently, the catalytic activity of the in situ activated [(^Me^N_2_N)_2_Ni] was then tested as a catalyst in a model hydrodehalogenation reaction (Table S1, entries 1 and 2).^41^ Importantly, the conversion and catalytic efficacy were found to be comparable to those obtained when [(^Me^N_2_N)­NiCl] was used directly as the catalyst.^39^

**Chart 2:**
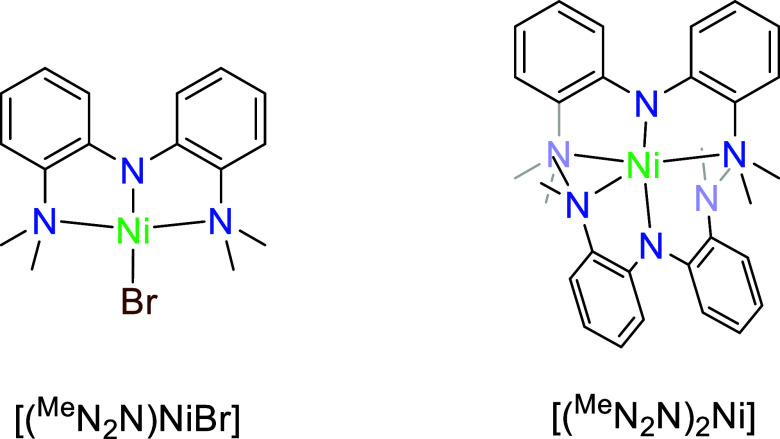
Chemical structure of [(^Me^N_2_N)­NiBr] and [(^Me^N_2_N)_2_Ni].

### Generality of Homoleptic Reactivation

The scope of this strategy was expanded by examining the effect of simple inorganic salts, such as alkali halides, given their widespread use as catalytic additives despite the limited understanding of their mechanistic roles.^42,43^ To probe this effect, complex **4** was treated with various alkali salts. For example, upon mixing with LiBr, UV–Vis spectroscopy showed clean conversion of the homoleptic species **4** to Cz^*t*Bu^(Pyr^*i*Pr^)_2_NiBr, consistent with cation-assisted *in-situ* activation (Figure S14).

The practical relevance of this activation pathway was then evaluated in the previously described hydrodehalogenation reaction, where sodium alkoxides served as both base and alkali metal source. Under these conditions, external NiBr_2_ was no longer required, with conversion efficiencies comparable to those using preadded NiBr_2_ (Table S1, entries 3 and 4). This finding highlights the critical role of alkali metal cations in promoting in situ activation and suppressing deactivation via the formation of homoleptic complexes.

Ultimately, to explore the generality of the approach, we also investigated whether other first–row transition metal halides could promote analogous transformations. Specifically, homoleptic Fe­(II) and Co­(II) complexes supported by the Cz^*t*Bu^(Pyr^*i*Pr^)_2_^–^ ligand were synthesized^30,31^ and treated with their corresponding metal halides (CoCl_2_ and FeCl_2_). In both cases, ^1^H NMR spectroscopy revealed clean conversion of the homoleptic species to the corresponding monohalide complexes (Cz^*t*Bu^(Pyr^*i*Pr^)_2_CoCl and Cz^*t*Bu^(Pyr^*i*Pr^)_2_FeCl) (Figures S15 and S16), analogous to the behavior observed for nickel counterparts. Furthermore, we examined cross-reactivity by treating complex **4** with CoCl_2_ or FeCl_3_, respectively, which led to statistically distributed mixtures of Cz^*t*Bu^(Pyr^*i*Pr^)_2_NiCl^29^ and Cz^*t*Bu^(Pyr^*i*Pr^)_2_CoCl^31^ or Cz^*t*Bu^(Pyr^*i*Pr^)_2_FeCl_2_,^44^ respectively, in approximately 1:1 ratios as determined by ^1^H NMR analysis (Figures S17 and 18). This mixed-metal reactivity demonstrates an effective pathway for disrupting homoleptic aggregation and generating catalytically relevant monohalide species in situ. Moreover, the formation of mixed-metal complex mixtures raises intriguing possibilities for future applications in cooperative or tandem catalysis, where two distinct metal centers could operate synergistically within the same reaction environment.

## Conclusions

In summary, this study demonstrates that homoleptic Ni­(II) pincer complexes, commonly regarded as irreversible catalytic dead ends, can be converted to catalytically relevant monohalide species through a bimetallic reactivation pathway. Spectroscopic and computational studies support the formation of a transient binuclear intermediate during this transformation. Time-dependent DFT calculations reveal characteristic MMCT transitions that provide an electronic fingerprint for this species and offer insight into its electronic structure. The observation that simple halide salts such as LiBr promote regeneration of the active LNiBr complex further highlights the dynamic nature of these systems in solution. Preliminary observations with cobalt and iron analogues suggest that related reactivation pathways may operate across multiple first–row transition metals.

## Experimental Section

All reagents were purchased from chemical vendors and used as received without further purification, unless otherwise specified. All manipulations were performed under a nitrogen atmosphere using standard Schlenk techniques or within a Vigor N_2_-atmosphere glovebox, unless otherwise specified. Glassware was dried at 150 °C overnight. Diethyl ether, *n*-pentane, tetrahydrofuran, and toluene were purified using a solvent purification system (LC Technology Solutions Inc.). Before use, an aliquot of each solvent was tested with a drop of sodium benzophenone ketyl in THF solution. HCz^*t*Bu^(Pyr^H^)_2_, HCz^*t*Bu^(Pyr^Me^)_2_, HCz^*t*Bu^(Pyr^*i*Pr^)_2_ and Cz^*t*Bu^(Pyr^*i*Pr^)_2_NiBr were prepared according to literature procedures.^29,^ ^45^ ^1^H NMR data were recorded on Bruker 500 MHz spectrometer at 22 °C. Resonances in the ^1^H NMR spectra are referenced to residual C_6_D_5_H at δ = 7.16 ppm. UV–visible spectra were recorded on a JASCO V-750 spectrophotometer. Mass spectra were recorded on Bruker Autoflex Speed, Triple Quadrupole, MALDI. DFT calculations have been performed with the Gaussian16 software packages. A more detailed explanation of the experimental and computational details can be found in the Supporting Information.

## Supplementary Material



## Notes

Experimental information and compound characterization data; X-ray crystallographic data; NMR spectra (PDF)
