# Supplementary material for: From Dead Ends to Catalyst Reservoirs: Bimetallic Reactivation of Homoleptic Nickel Pincer Complexes
Source: Inorg Chem. 2026 Jun 2;65(23):13097–106. doi: 10.1021/acs.inorgchem.6c01379 (PMC13273811; doi:10.1021/acs.inorgchem.6c01379)
Supplement: Supplementary file 1 [file ic6c01379_si_001.pdf]

Supplementary Information

From Dead Ends to Catalyst Reservoirs: Bimetallic Reactivation of Homoleptic Nickel Pincer Complexes

Jun-Yang Ye,[†] *Zheng-Feng Zhang,* [‡] *Matthias Zeller,* [§] *Ming-Der Su,* [*,‡,ǁ] *Wei-Tsung Lee* [*,†]

[†] Department of Chemistry, National Central University, Taoyuan 32001, Taiwan.

[‡] Department of Applied Chemistry, National Chiayi University, Chiayi 60004, Taiwan

[§] Department of Chemistry, Purdue University, West Lafayette, IN 47907, United States.

[ǁ] Department of Medicinal and Applied Chemistry, Kaohsiung Medical University, Kaohsiung 80708, Taiwan

*Email: midesu@mail.ncyu.edu.tw.

*Email: wlee5@ncu.edu.tw.

Table of Contents

	Page
General Synthesis of Ni Complexes	S3
General Catalytic Procedures	S6
**Table S1.** Catalytic hydrodehalogenation of $[(^{Me}N_2N)_2Ni]$	S6
Crystallography	S7
**Table S2.** Crystal data and structure refinement detail for **1−4**	S8
**Table S3.** Crystal data and structure refinement detail for **1(THF)₂**, $Cz^{tBu}(Pyr^{Me})_2NiBr$, and $(Cz^{tBu}(Pyr^{iPr})_2)_2Ni$-Ag-$PF_6$	S10
Computational Methods	S11
**Figure S1**. Molecular structure of $[(Cz^{tBu}(Pyr^{H})_2)_2Ni]$ (**2**)	S12
**Figure S2**. Molecular structure of **1(THF)$_2$**	S12
**Figure S3**. Molecular structure of **3** and **4**	S13
**Figure S4**. Side view of molecular structure of **2–4**	S13
**Figure S5.** Electronic absorption spectra **3** and $Cz^{tBu}(Pyr^{Me})_2NiBr$	S14
**Figure S6.** Electronic absorption spectra **4** and $Cz^{tBu}(Pyr^{iPr})_2NiBr$	S14
**Figure S7.** UV–Vis monitoring of the reaction between complex **2** and $NiBr_2$ in toluene at 80 °C over 12h	S15
**Figure S8.** Electronic absorption spectra **2** and **1(THF)$_2$**	S15
**Figure S9.** UV–Vis monitoring of the reaction between complex **2** and $NiBr_2$ in MeCN at 80 °C	S16
**Figure S10.** UV–Vis monitoring of the reaction between complex **4** and $NiBr_2$ in toluene at 80 °C in the first 40 mins	S16
**Figure S11.** UV–Vis monitoring of the reaction between complex **4** and $NiBr_2$ in toluene at 80 °C over the last 70 mins	S17
**Figure S12.** Electronic absorption spectra of $(Cz^{tBu}(Pyr^{iPr})_2)_2Ni$-Ag-$PF_6$	S17
**Figure S13.** ^1H NMR spectra recorded during monitoring of the reaction of $[(^{Me}N_2N)_2Ni]$ and $NiBr_2$ at 80 °C from 0 to 3h	S18
**Figure S14.** UV–Vis monitoring of the reaction between complex **4** and LiBr in toluene at 80 °C	S18
**Figure S15.** ^1H NMR spectrum of reaction product of $[(Cz^{tBu}(Pyr^{iPr})_2)_2Co]$ and $CoCl_2$ in C_6D_6	S19
**Figure S16.** ^1H NMR spectrum of reaction product of $[(Cz^{tBu}(Pyr^{iPr})_2)_2Fe]$ and $FeCl_2$ in C_6D_6	S19
**Figure S17.** ^1H NMR spectrum of reaction product of $[(Cz^{tBu}(Pyr^{iPr})_2)_2Ni]$ and $CoCl_2$ in C_6D_6	S20
**Figure S18.** ^1H NMR spectrum of reaction product of $[(Cz^{tBu}(Pyr^{iPr})_2)_2Ni]$ and $FeCl_3$ in C_6D_6	S20
**Figure S19.** ^1H NMR spectrum of hydrodehalogenation reaction of entry 1.	S21
**Figure S20.** ^1H NMR spectrum of hydrodehalogenation reaction of entry 2.	S21
**Figure S21.** ^1H NMR spectrum of hydrodehalogenation reaction of entry 3.	S22
**Figure S22.** ^1H NMR spectrum of hydrodehalogenation reaction of entry 4.	S22
**Table S4.** Cartesian coordinates (Å) of all optimized species	S23
**Figure S23.** Simulated UV-Vis spectra for **3^{Int}**.	S71
**Table S5.** Donor and acceptor orbitals of **3^{Int}** for the excited states	S71
**Figure S24.** Selected molecular orbitals of **3^{Int}** (α-spin)	S72
**Figure S25.** Selected molecular orbitals of **3^{Int}** (ß-spin)	S73
**Figure S26.** Simulated UV-Vis spectra for **4^{Int}**.	S74

Table S6. Donor and acceptor orbitals of $\mathbf{4^{Int}}$ for the excited states S74
Figure S27. Selected molecular orbitals of $\mathbf{4^{Int}}$ (α-spin) S75
Figure S28. Selected molecular orbitals of $\mathbf{4^{Int}}$ (ß-spin) S76
Figure S29. ^{1}H NMR spectrum of **1** in THF-d_8 S77
Figure S30. ^{1}H NMR spectrum of **2** in C_6D_6 S77
Figure S31. ^{1}H NMR spectrum of **3** in C_6D_6 S78
Figure S32. ^{1}H NMR spectrum of **4** in C_6D_6 S78
Figure S33. ^{1}H NMR spectrum of $Cz^{tBu}(Pyr^{Me})_2NiBr$ in acetone-d_6 S79
Figure S34. ^{1}H NMR spectrum of $(Cz^{tBu}(Pyr^{iPr})_2)_2Ni$-Ag-$PF_6$ in acetone-d_6 S79
References S80

General Synthesis of Ni Complexes.

*Synthesis of $Cz^{tBu}(Pyr^{H})_2NiBr$ (**1**).* To a Schlenk flask was added 0.188 g (0.457 mmol) of $HCz^{tBu}(Pyr^{H})_2$, 0.048 g (0.457 mmol) of LDA and 0.157g (0.483 mmol) of $NiBr_2(THF)_{1.5}$. The solids were dissolved by adding THF (15 mL), then the mixture was stirred for 4h at 80°C under N_2 atmosphere. After cooling down to room temperature, the solution was filtered through a celite pad, and volatiles were removed under vacuum. The resulting yellow solid was recrystallized from concentrated THF to afford a yellow-green solid (0.225 g, 71%). Crystals suitable for X-ray diffraction were grown from a concentrated toluene solution at ambient temperature. ^{1}H NMR (500 MHz, THF-d_8, δ): 62.9, 43.8 (br), 36.4, 29.2, 9.5, 2.3. μ_{eff} (THF-d_8): 2.9 ± 0.2 μ_B. UV−vis (THF at 25 °C) λ_{max} (ε [$M^{-1}\cdot cm^{-1}$]): 347.5(15200), 404(13600), 422.5(15900). UV−vis (MeCN at 25 °C) λ_{max} (ε [$M^{-1}\cdot cm^{-1}$]): 343(13300), 401(11100), 419(12100), 524(500). HR-MS for $\{C_{26}H_{28}BrN_5Ni\}^+$: Calcd, 547.0876; Found, 547.0849.

*General synthesis of $(Cz^{tBu}(Pyr^{R})_2)_2Ni$ (R= H(**2**), Me(**3**), ^{i}Pr(**4**)).* 0.729 mmol of $HCz^{tBu}(Pyr^{R})_2$ and 0.078 g (0.729 mmol) of LDA were dissolved in 10 mL of THF and stirred for 30 mins at ambient temperature under N_2 atmosphere. Then 0.112 g (0.365 mmol) of $NiBr_2(DME)$ in 3 mL THF solution were added and the resulting slurry was stirred overnight at ambient temperature. Volatiles were removed under vacuum, the residue was extracted with toluene

and filtered through a celite pad. The filtrate was dried under vacuum to afford a bright yellow solid.

*$(Cz^{tBu}(Pyr^{H})_2)_2Ni$ (**2**)*: Yield: 84%. Crystals suitable for X-ray diffraction were grown from a concentrated toluene solution at −30 °C. ^{1}H NMR (500 MHz, C_6D_6, δ): 64.5 (br), 30.7, 25.5, 6.8 (br),1.9. μ_{eff} (C_6D_6): 2.9 ± 0.2 μ_B. UV−vis (toluene at 25 °C) $\lambda_{\max}$ (ε [$M^{-1}\cdot cm^{-1}$]): 346.5(21200), 382.5(9000), 404(18500), 424.5(23200). UV−vis (THF at 25 °C) $\lambda_{\max}$ (ε [$M^{-1}\cdot cm^{-1}$]): 382.5(10000), 402(19300), 422.5(23900). HR-MS for $\{C_{52}H_{56}N_{10}Ni\}^+$: Calcd, 878.4037; Found, 878.3999. Anal. Calcd for $C_{52}H_{56}N_{10}N$: C, 70.90; H, 6.42; N, 15.92. Found: C, 70.64; H, 6.78; N, 15.89.

*$(Cz^{tBu}(Pyr^{Me})_2)_2Ni$ (**3**)*: Yield: 72%. Crystals suitable for X-ray diffraction were grown from a concentrated toluene solution at −30 °C. ^{1}H NMR (500 MHz, C_6D_6, δ): 32.4, 25.6, 23.9, 9.6, 9.3 (br), 2.2. μ_{eff} (C_6D_6): 2.4 ± 0.2 μ_B. UV−vis (toluene at 25 °C) $\lambda_{\max}$ (ε [$M^{-1}\cdot cm^{-1}$]): 352(11700), 405(15800), 423.5(19400). UV−vis (THF at 25 °C) $\lambda_{\max}$ (ε [$M^{-1}\cdot cm^{-1}$]): 351.5(9000), 402(12800), 420.5(15400). HR-MS for $\{C_{56}H_{64}N_{10}Ni\}^+$: Calcd, 934.4663; Found, 934.4649. Anal. Calcd for $C_{56}H_{64}N_{10}Ni\cdot 3H_2O$: C, 67.95; H, 7.13; N, 14.15. Found: C, 68.18; H, 7.17; N, 14.09.

*$(Cz^{tBu}(Pyr^{iPr})_2)_2Ni$ (**4**)*: Yield: 41%. Crystals suitable for X-ray diffraction were grown by slow evaporation of a n-pentane solution at ambient temperature. ^{1}H NMR (500 MHz, C_6D_6, δ): 30.1, 25.4, 23.9, 7.4, 2.3, 0.3. μ_{eff} (C_6D_6): 2.4 ± 0.2 μ_B. UV−vis (toluene at 25 °C) $\lambda_{\max}$ (ε [$M^{-1}\cdot cm^{-1}$]): 369.5(39400), 404(14600), 422.5(17000). UV−vis (THF at 25 °C) $\lambda_{\max}$ (ε [$M^{-1}\cdot cm^{-1}$]): 349(14800), 402(15800), 420.5(18700). Anal. Calcd for $C_{64}H_{80}N_{10}Ni$: C, 73.34; H, 7.69; N, 13.36. Found: C, 72.93; H, 7.83; N, 13.27.

Synthesis of $Cz^{tBu}(Pyr^{Me})_2NiBr$. 0.168 g (0.381 mmol) of $HCz^{tBu}(Pyr^{Me})_2$ and 0.041 g (0.381 mmol) of LDA were dissolved in 10 mL of THF under N_2 atmosphere. The resulting yellow

slurry was stirred for 15 mins, the mixture was then added to 0.131 g (0.400 mmol) of $NiBr_2(THF)_{1.5}$ in 5 mL of THF and stirred for an additional 1h at ambient temperature. The solution was filtered through a celite pad and volatiles were removed under vacuum to afford a dark green solid. The solid was washed with n-pentane (3 x 10mL) and dried to yield a dark green powder (0.250g, 95%). Crystals suitable for X-ray diffraction were grown from a concentrated toluene solution at −30 °C. ^{1}H NMR (500 MHz, Acetone-d_6, δ): 71.9, 55.9, 32.2, 14.9, 7.3, −0.4 (br). μ_{eff} (Acetone-d_6): 2.5 ± 0.2 μ_B. UV−vis (toluene at 25 °C) λ_{max} (ε [$M^{-1}\cdot cm^{-1}$]): 398(11828), 646(3466). UV−vis (THF at 25 °C) λ_{max} (ε [$M^{-1}\cdot cm^{-1}$]): 348(11362), 406(10664), 422.5(11446). HR-MS for $\{C_{28}H_{32}BrN_5Ni\}^+$: Calcd, 575.1189; Found, 575.1161.

Synthesis of $(Cz^{tBu}(Pyr^{iPr})_2)_2Ni\text{-}Ag\text{-}PF_6$. 0.030 g (0.029 mmol) of $(Cz^{tBu}(Pyr^{iPr})_2)_2Ni$ was dissolved in 5 mL of THF and stirred for 5 mins at ambient temperature under N_2 atmosphere. Then 0.007 g (0.029 mmol) of $AgPF_6$ in 3 mL THF solution were added, the resulting purple solution was stirred for an additional 1h at ambient temperature. The solution was filtered through a celite pad and volatiles were removed under vacuum to afford a dark purple solid. The solid was washed with *n*-pentane (3 x 5mL) and dried to yield a dark purple powder (0.030g, 80%). Crystals suitable for X-ray diffraction were grown by slow diffusion n-pentane into THF solution at −30 °C. ^{1}H NMR (500 MHz, Acetone-d_6, δ): 48.6, 46.9, 38.5, 21.9 (br), 18.4, 12.6, 10.5, 6.9, 4.1, 3.7, 1.9, 0.3, -0.1, -1.7, -2.7. μ_{eff} (Acetone-d_6): 2.8 ± 0.2 μ_B. UV−vis(toluene at 25 °C) λ_{max} (ε [$M^{-1}\cdot cm^{-1}$]): 374(4600), 389.5(5100), 528.5(2100). HR-MS for $\{C_{64}H_{80}N_{10}NiAg\}^+$: Calcd, 1153.4966; Found, 1153.4933.

General Catalytic Procedures

To a flask was added 2 equiv of NaO^{i}Pr, 1 equiv of hexamethylbenzene, 1 equiv of $C_{14}H_{29}Cl$, and a solution of 10 mol % of $(^{Me}N_2N)_2Ni$ in 0.5 mL of THP. To the resulting solution, a solution of 2 equiv of silane in 0.5 mL of THP was added dropwise over 30 minutes. After 6 hours, volatiles were removed under vacuum (ca. 20 mmHg) at room temperature. The residue was extracted with n-hexane and filtered through a pad of silica gel. Product conversion was determined by ^{1}H NMR spectroscopy relative to hexamethylbenzene as an internal standard. Each catalytic experiment, including blank reactions, was independently performed twice, and the reported values represent the average of two runs.

Table S1. Catalytic hydrodehalogenation of $[(^{Me}N_2N)_2Ni]$.

$$C_{14}H_{29}Cl + \text{silane} \xrightarrow[\text{NaO}^i\text{Pr}]{10\text{ mol }\%(^{Me}N_2N)_2Ni} C_{14}H_{30}$$

entry	silane	conversion (%)
1[a]	$Me(EtO)_2SiH$	72
2[a]	$PhSiH_3$	78
3[b]	$Me(EtO)_2SiH$	38
4[b]	$PhSiH_3$	62

[a] 5 mol% catalyst and 1 equiv of $NiBr_2$.

[b] 10 mol% catalyst, $[(^{Me}N_2N)_2Ni]$.

[c] Conversion was determined by ^{1}H NMR spectroscopy using hexamethylbenzene as an internal standard by monitoring the decrease of the methylene resonance adjacent to chlorine in $C_{14}H_{29}Cl$.

Crystallography

Data were collected using Bruker Quest CMOS diffractometer. For compound **2**, a diffractometer equipped with a IμS microsource with a laterally graded multilayer (Goebel) mirror for monochromatization (Cu-Kα radiation, λ = 1.54178 Å) was used. The instrument used for all other compounds featured sealed tube X-ray source with graphite monochromated Mo-Kα radiation (λ = 0.71073 Å). A single crystal of each compound was mounted on a Mitegen micromesh mount using a trace of mineral oil and cooled *in situ* to 100 or 150 K for data collection. Frames were collected, reflections were indexed and processed, and the files scaled and corrected for absorption using Apex3 or Apex5.[1] For all structures, the intensity data were corrected for absorption using multi-scan techniques (SADABS).[2] The space groups were assigned and the structures were solved by direct methods using XPREP within the SHELXTL suite of programs and refined by full matrix least squares against F^2 with all reflections using Shelxl 2016 or 2019 using the graphical interface Shelxle.[3-5] If not specified otherwise, H atoms attached to carbon and nitrogen atoms and hydroxyl hydrogens were positioned geometrically and constrained to ride on their parent atoms, with carbon hydrogen bond distances of 0.95 Å for alkene and aromatic C–H, and 0.99 and 0.98 Å for aliphatic CH_2 and CH_3 moieties, respectively. Methyl H atoms were allowed to rotate but not to tip to best fit the experimental electron density. U_{iso}(H) values were set to a multiple of U_{eq}(C) with 1.5 for CH_3 and 1.2 for CH_2 and CH units, respectively. Additional experimental details for all structures are given in the SI. Structures, in CIF format, have been deposited with the Cambridge Crystallographic Data Centre, CCDC 2535506-2535512. These data can be obtained free of charge from The Cambridge Crystallographic Data Centre via www.ccdc.cam.ac.uk/data_request/cif.

Table S2. Crystal data and structure refinement detail for **1**−**4**.

	**1**[a]	**2**[d]	**3**[c]	**4**[d]
Empirical formula	$2(C_{26}H_{28}BrN_5Ni)$, (C_5H_{12})	$C_{52}H_{56}N_{10}Ni$, (C_7H_8)	$C_{56}H_{64}N_{10}Ni$, $3(C_7H_8)$	$C_{64}H_{80}N_{10}Ni$, $2(C_5H_{12})$
Formula weight	1170.45	971.91	1212.28	1192.38
Space group	*C*2/*c*	*P*2_1/*c*	*C*2/*c*	*C*2/*c*
a/Å	21.683(7)	13.4670(3)	54.924(3)	40.170(7)
b/Å	14.844(4)	21.7388(5)	13.5762(9)	13.427(2)
c/Å	17.985(5)	18.0780(4)	18.0165(9)	29.607(5)
a/°	90	90	90	90
b/°	108.261(13)	105.7650(10)	103.073(2)	120.550(7)
g/°	90	90	90	90
$V/Å^3$	5497(3)	5093.4(2)	13086.0(13)	13752(4)
Z	4	4	8	8
D_{calcd}, g cm^{-3}	1.414	1.267	1.231	1.152
F(000)	2424	2064	5184	5168
Temp, K	100	150	150	104
R(F), %	6.22	4.73	5.03	6.11
R_w(F), %	17.17	10.76	12.02	14.43

Special refinement details:

[a] One tert-butyl group showed signs of minor disorder. It was refined as rotationally disordered with both units restrained to have similar geometries, and U^{ij} components of ADPs of disordered atoms were restrained to be similar. The occupancy ratio refined to 0.917(5) to 0.083(5).

[b] One tert-butyl group was refined as disordered by rotation. The major and minor moieties were each restrained to have similar geometries using SAME restraint. U^{ij} components of ADPs

for disordered atoms closer to each other than 2.0 Angstrom were restrained to be similar. Subject to these conditions the occupancy ratio refined to 0.593(3) to 0.407(3).

[c] Two toluene molecules were disordered across a mirror plane. The molecule of C78 was restrained to be close to planar. U^{ij} components of ADPs for disordered atoms closer to each other than 2.0 Angstrom.

[d] Two solvate n-pentane molecules were refined as disordered. All disordered moieties were each restrained to have similar geometries using SAME restraints (same C65 > C69). C-C distances of pentane molecule were restrained to target values of 1.55(2) Angstrom for CH_2-CH_3 distances, 1.53(2) Angstrom for CH_2-CH_2 distances, and all 1,3 distances were restrained to a target value of 2.50(2) Angstrom. U^{ij} components of ADPs for disordered atoms closer to each other than 2.0 Angstrom were restrained to be similar. Subject to these conditions the occupancy ratio refined to 0.531(8) to 0.469(8) for molecule of C65, to 0.595(9) to 0.405(9) for molecule of C70. One pyrazole arm of N6 was refined as disordered. The major and minor disordered moieties were each restrained to have similar geometries using SAME restraints, atom N6 and C36 were omitted from the disorder, N7 was using EADP constraint to give the major and minor moiety atoms identical ADPs. U^{ij} components of ADPs for disordered atoms closer to each other than 2.0 Angstrom were restrained to be similar. Subject to these conditions the occupancy ratio refined to 0.740(18) to 0.260(18).

Table S3. Crystal data and structure refinement detail for **1(THF)$_2$**, Cz^{tBu}(Pyr^{Me})$_2$NiBr, and (Cz^{tBu}(Pyr^{iPr})$_2$)$_2$Ni-Ag-PF$_6$.

	**1(THF)$_2$**[a]	Cz^{tBu}(Pyr^{Me})$_2$NiBr	(Cz^{tBu}(Pyr^{iPr})$_2$)$_2$Ni-Ag-PF$_6$[b]
Empirical formula	$C_{34}H_{44}BrN_5NiO_2$	$C_{28}H_{32}BrN_5Ni$, (C_7H_8)	$C_{72}H_{96}N_{10}AgNiO_2$, 3($C_4H_8O$), F_6P
Formula weight	693.33	669.34	1589.33
Space group	*P*2$_1$/*c*	*P*2$_1$/*c*	*C*2/*c*
a/Å	25.5004(11)	14.8321(5)	30.0739(5)
b/Å	13.9767(5)	19.4939(6)	18.0927(4)
c/Å	19.2967(8)	10.9354(3)	15.3386(3)
a/°	90	90	90
b/°	112.013(2)	90.3600(10)	108.3840(10)
g/°	90	90	90
V/Å^3	6376.2(5)	3161.75(17)	7920.1(3)
Z	8	4	4
D_{calcd}, g cm^{-3}	1.445	1.406	1.333
F(000)	2896	1392	3352
Temp, K	100	150	150
R(F), %	4.10	3.69	3.75
R_w(F), %	10.62	7.64	7.86

Special refinement details:

[a] The structure is twinned by emulating a double volume orthorhombic C-centered lattice and was refined as a 2-component twin. Twin matrix 1.000 0.000 0.991 0.000 -1.0000.000 0.000 0.000 -1.000. The twin ratio refined to 0.729(2) to 0.271(2). One solvate THF molecule was refined as disordered. The major and minor disordered moieties were each restrained to have

similar geometries as another not disordered THF molecule using SAME restraints. U^{ij} components of ADPs for disordered atoms closer to each other than 2.0 Angstrom were restrained to be similar. Subject to these conditions the occupancy ratio refined to 0.551(2) to 0.449(2).

[b] One tert-butyl group was refined as disordered by rotation. Atom C1 was omitted from the disorder using EXYZ and EADP constraints to give the major and minor moiety atoms identical ADPs and positions. The major and minor disordered moieties were each restrained to have similar geometries using SAME restraints. U^{ij} components of ADPs for disordered atoms closer to each other than 2.0 Angstrom were restrained to be similar. Subject to these conditions the occupancy ratio refined to 0.797(5) to 0.203(5).

Computational Methods

Based on the reported X-ray crystal structure and experimental observations, all molecular geometries were optimized in the open-shell triplet state at the UM06[6]-D3[7]/def2-SVP[8] level of theory. Frequency calculations were subsequently performed to confirm that the optimized structures correspond to true stationary points, characterized by internal forces below 0.000450 Hartree/Bohr and the absence of imaginary frequencies.

Time-dependent DFT (TD-DFT) calculations were carried out at the UM06-D3/def2-SVP level to simulate the UV–Vis spectra. A total of 50 excited states (NStates = 50) were included to improve the accuracy of the predicted absorption features. Solvent effects were not considered in the present study. All calculations were performed using the Gaussian 16 (Revision B.01)[9] program.

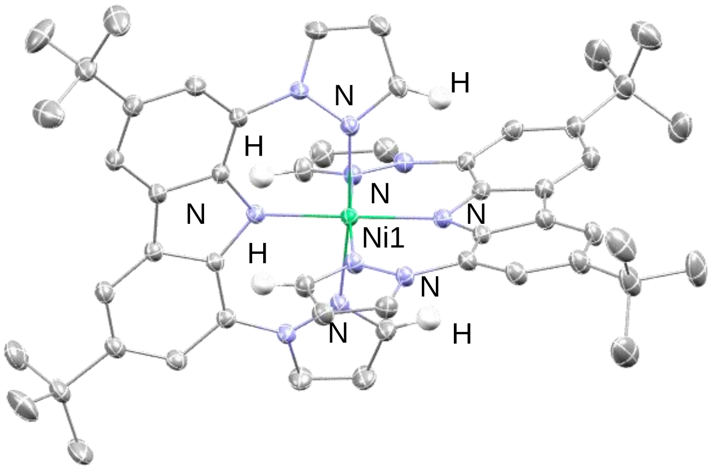

Figure S1. Molecular structure of [(Cz^{tBu}(Pyr^{H})$_2$)$_2$Ni] (**2**) with thermal ellipsoids at the 50% probability level. Non-coordinated solvent molecules and most hydrogen atoms are omitted for clarity. Color key: turquoise = Ni, blue = N, gray = C, white = H.

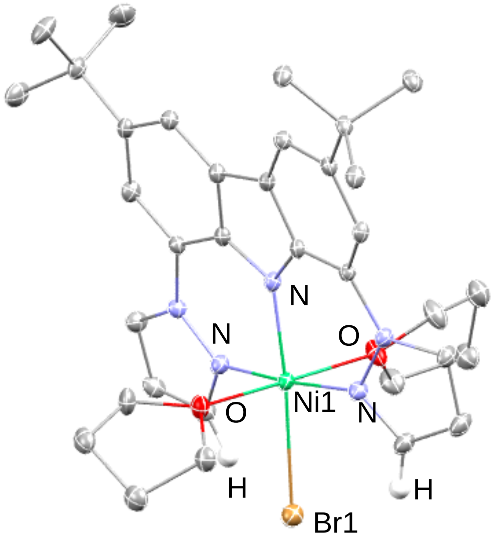

Figure S2. Molecular structure of **1(THF)$_2$** with thermal ellipsoids at the 50% probability level. One of two crystallographically independent molecules is shown. Most hydrogen atoms are omitted for clarity. Color key: turquoise = Ni, blue = N, gray = C, red = O, brown = Br, white = H.

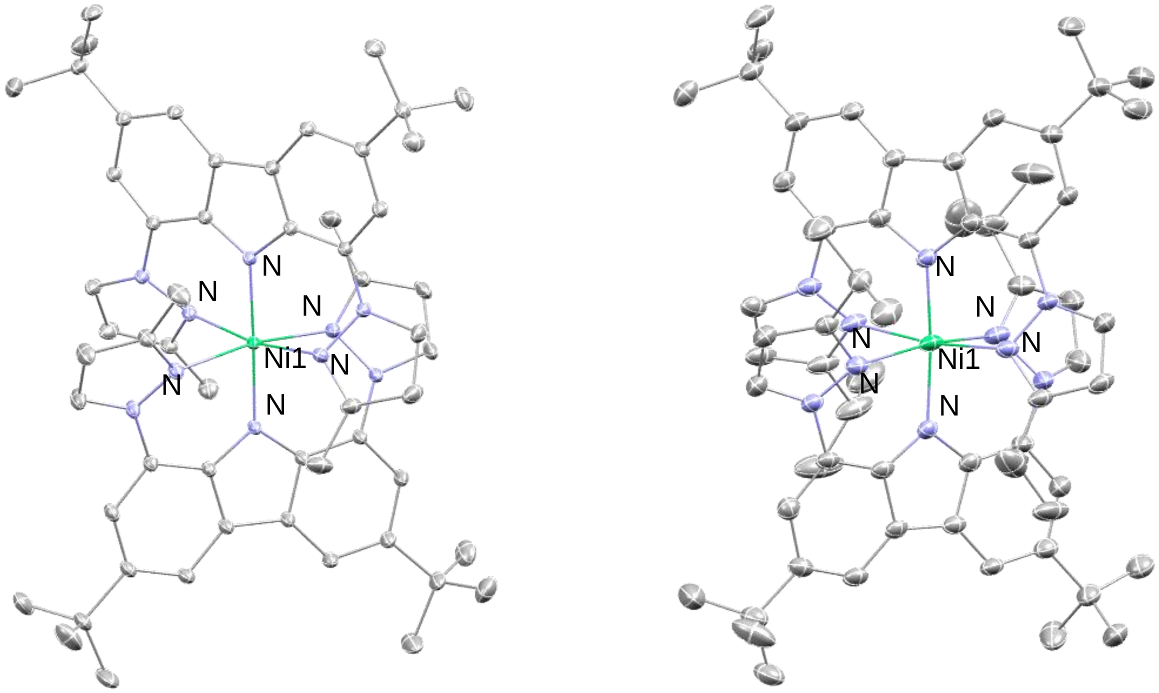

Figure S3. Molecular structure of **3** (left) and **4** (right) with thermal ellipsoids at the 50% probability level. Non-coordinated solvent molecules and hydrogen atoms are omitted for clarity. Color key: turquoise = Ni, blue = N, gray = C.

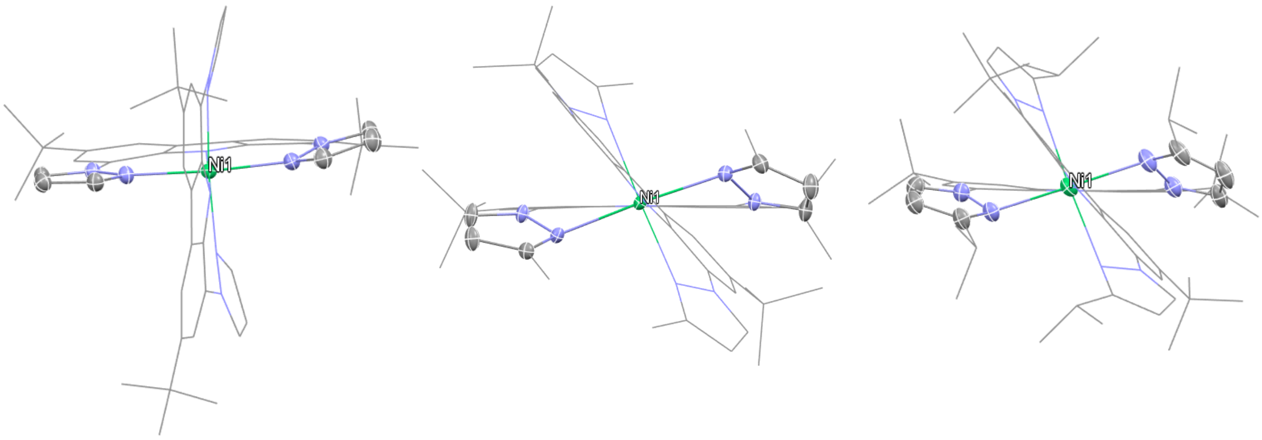

2 **3** **4**

Figure S4. Side view of molecular structure of **2**–**4** with selected thermal ellipsoids at the 50% probability level. Non-coordinated solvent molecules and hydrogen atoms are omitted for clarity. Color key: turquoise = Ni, blue = N, gray = C.

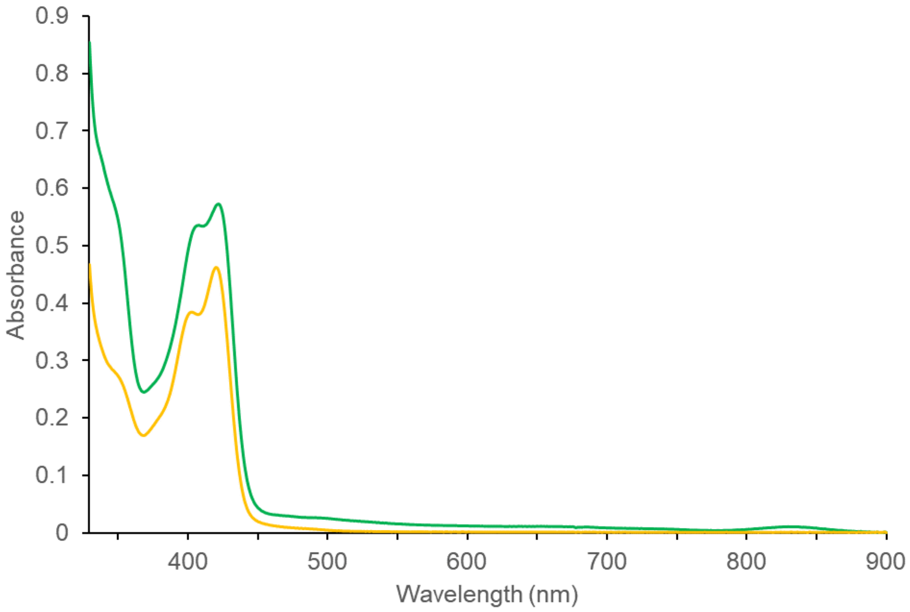

Figure S5. Electronic absorption spectra **3** (yellow line, 0.05 mM) and $Cz^{tBu}(Pyr^{Me})_2NiBr$ (green line, 0.05 mM) in THF.

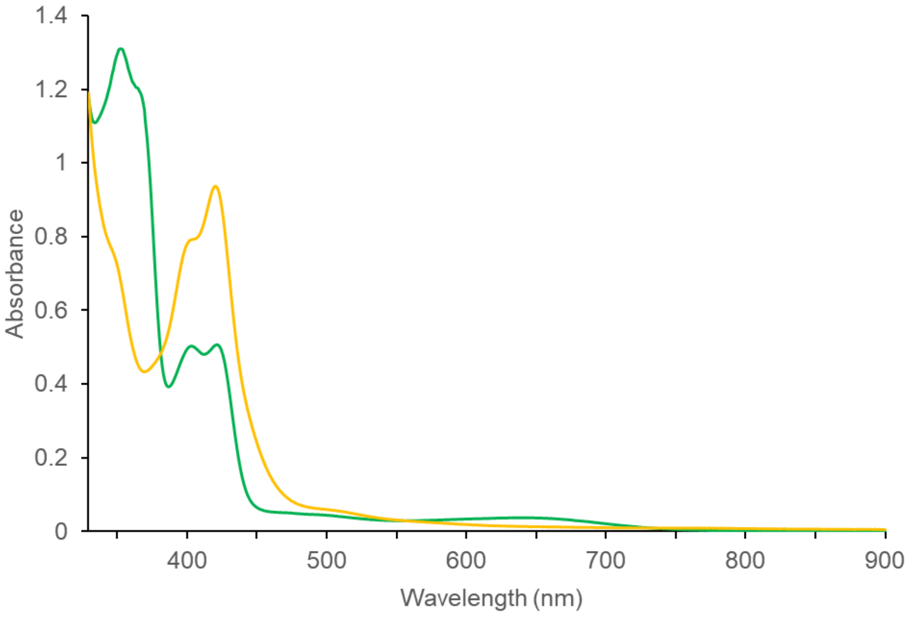

Figure S6. Electronic absorption spectra **4** (yellow line, 0.05 mM) and $Cz^{tBu}(Pyr^{iPr})_2NiBr$ (green line, 0.05 mM) in THF.

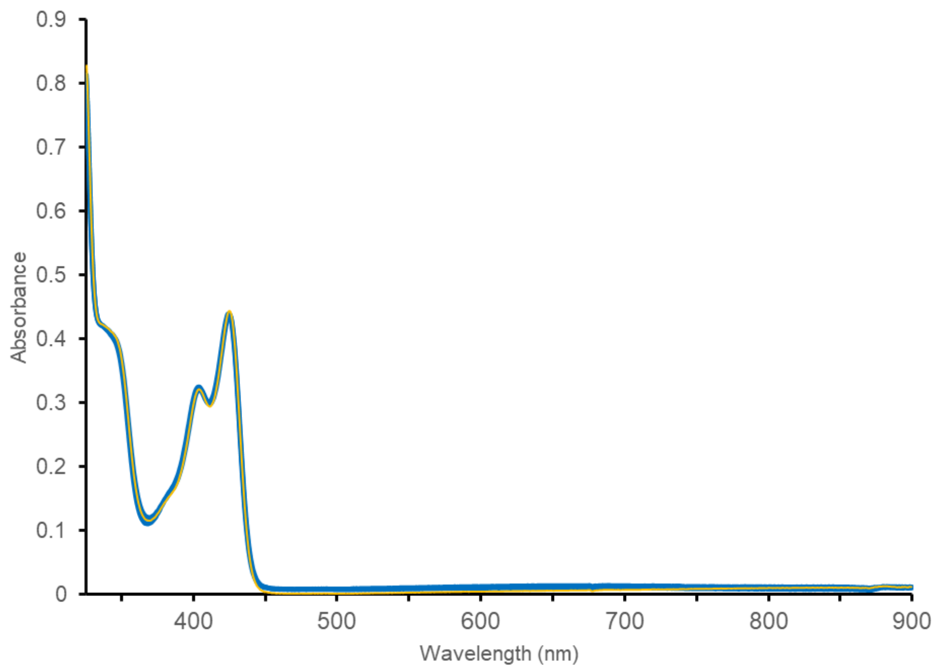

Figure S7. UV–Vis monitoring of the reaction between complex **2** and $NiBr_2$ in toluene at 80 °C over 12h.

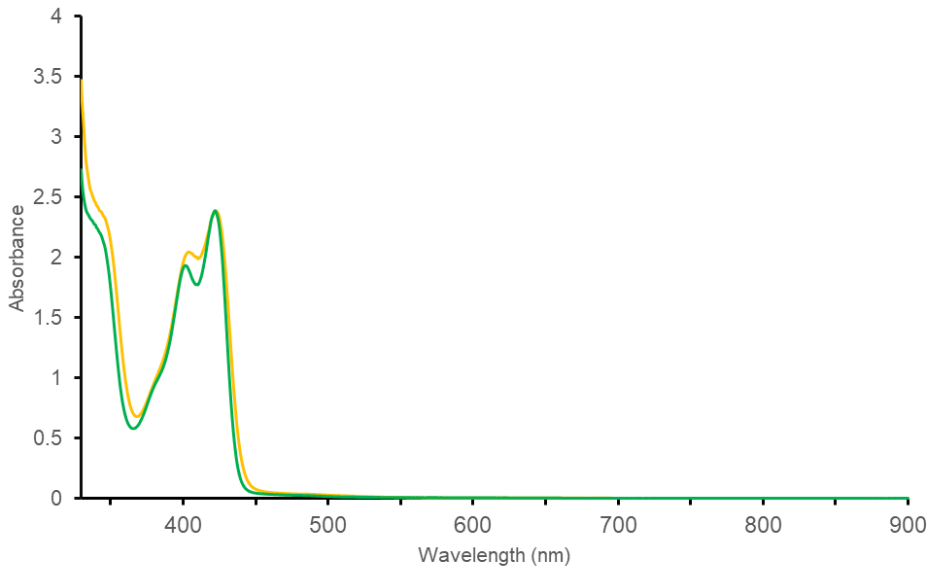

Figure S8. Electronic absorption spectra **2** (yellow line, 0.1 mM) and **1(THF)₂** (green line, 0.1 mM) in THF.

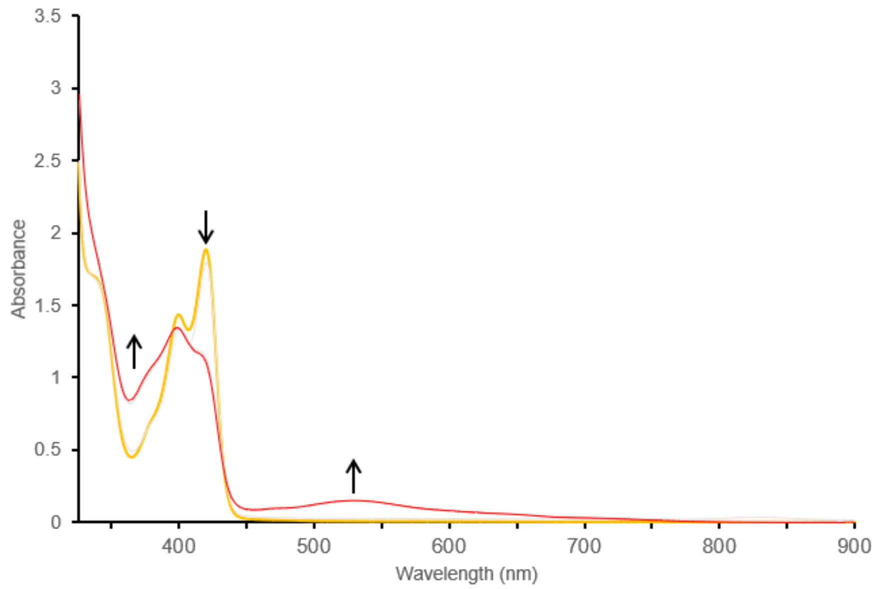

Figure S9. UV–Vis monitoring of the reaction between complex **2** and $NiBr_2$ in MeCN at 80 °C. The yellow trace shows the electronic absorption spectrum of **2** (0.1 mM) and the red trace corresponds to **1** generated *in-situ*.

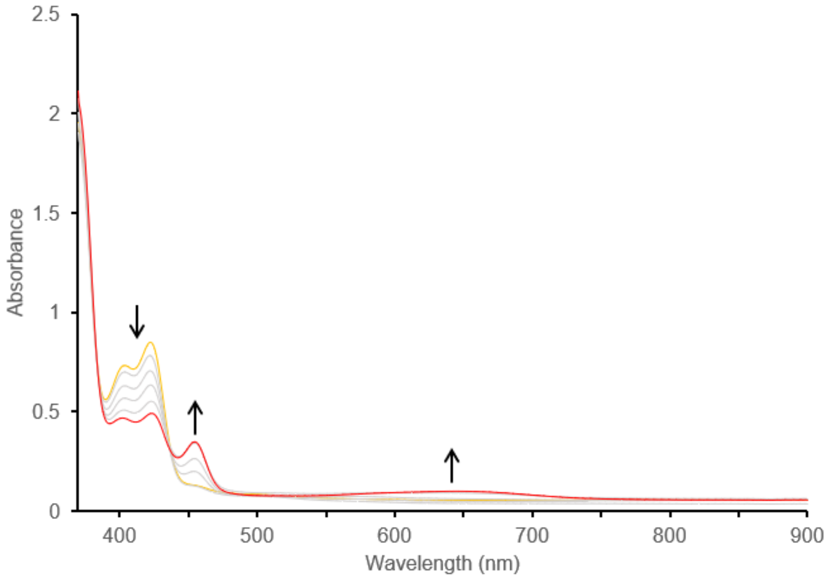

Figure S10. UV–Vis monitoring of the reaction between complex **4** and $NiBr_2$ in toluene at 80 °C in the first 40 mins. The yellow trace shows the electronic absorption spectrum of **2** (0.05 mM) and the red trace corresponds to intermediate generated *in-situ*.

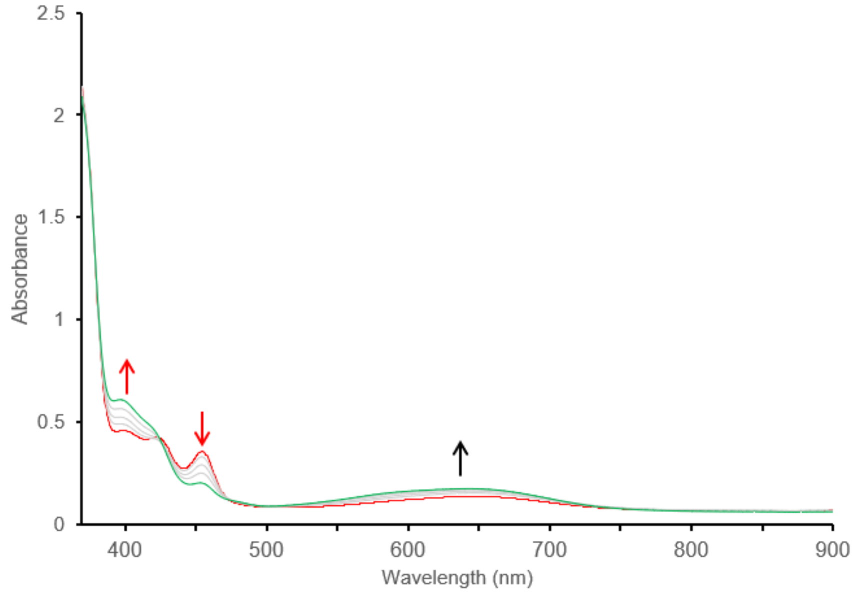

Figure S11. UV–Vis monitoring of the reaction between complex **4** and $NiBr_2$ in toluene at 80 °C over the last 70 mins. The green trace shows the electronic absorption spectrum of $Cz^{tBu}(Pyr^{iPr})_2NiBr$ (0.05 mM) and the red trace corresponds to the intermediate generated *in-situ*.

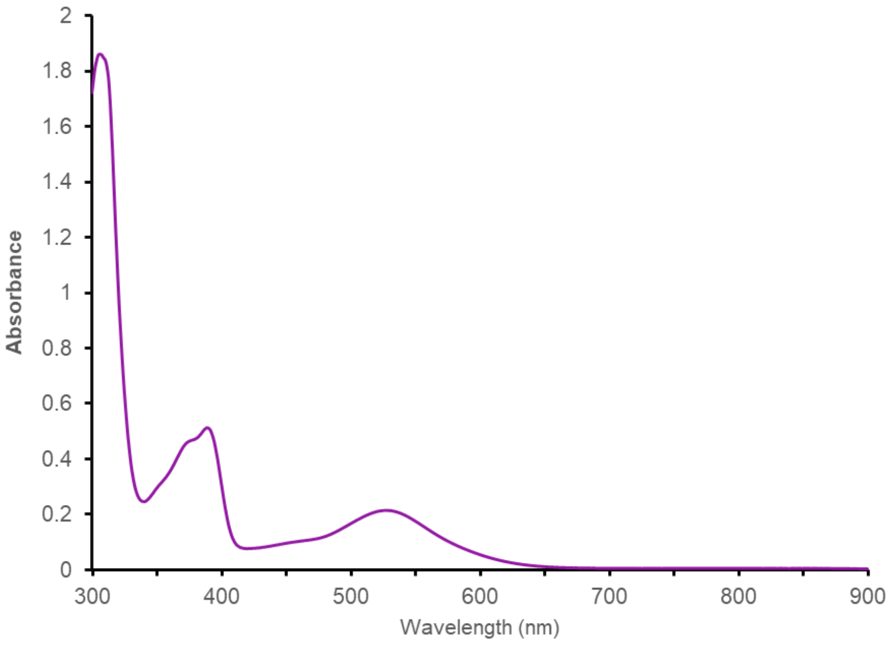

Figure S12. Electronic absorption spectra of $(Cz^{tBu}(Pyr^{iPr})_2)_2Ni\text{-}Ag\text{-}PF_6$ (0.05 mM) in toluene.

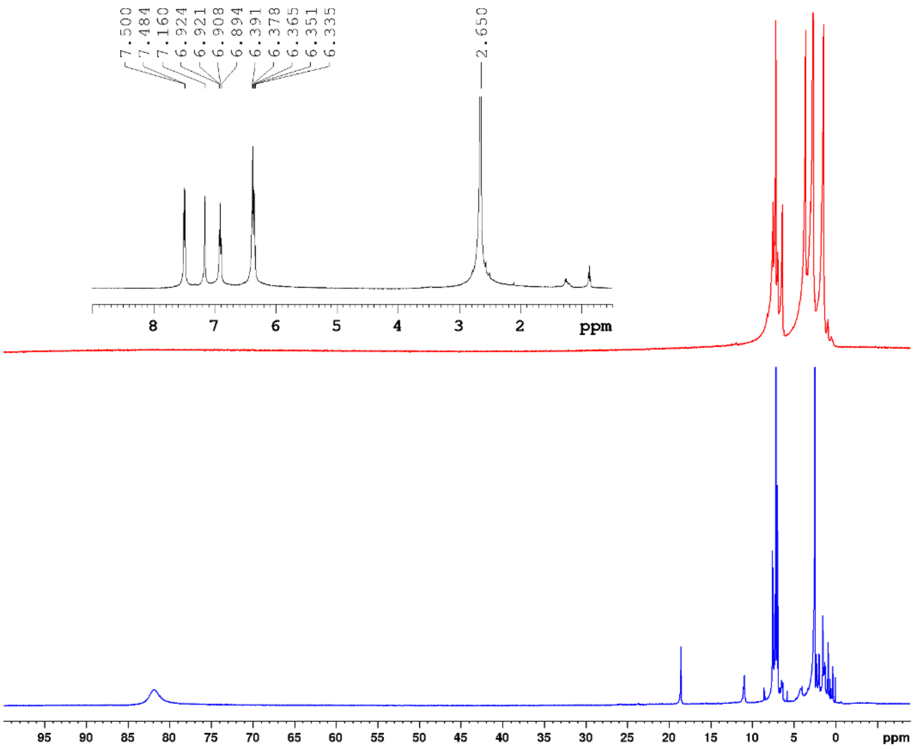

Figure S13. ^{1}H NMR spectra recorded during monitoring of the reaction of [($^{Me}N_2N)_2Ni$] and $NiBr_2$ at 80 °C from 0 (bottom) to 3h (top). Inset: zoom-in view of the top spectrum.

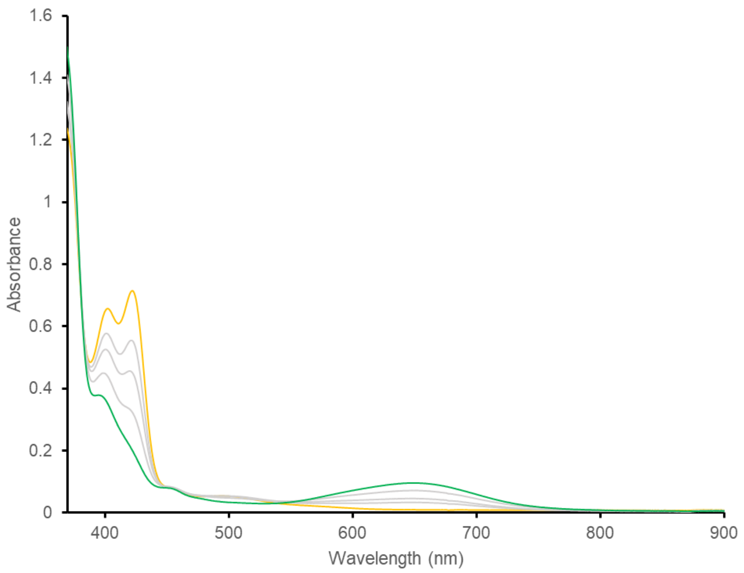

Figure S14. UV–Vis monitoring of the reaction between complex **4** and LiBr in toluene at 80 °C. The yellow trace shows the electronic absorption spectrum of **4** (0.05 mM) and the green trace corresponds to $Cz^{tBu}(Pyr^{iPr})_2NiBr$ generated in situ after 2.5 days under these reaction conditions. The reaction was monitored over an extended period due to the limited solubility of LiBr in toluene.

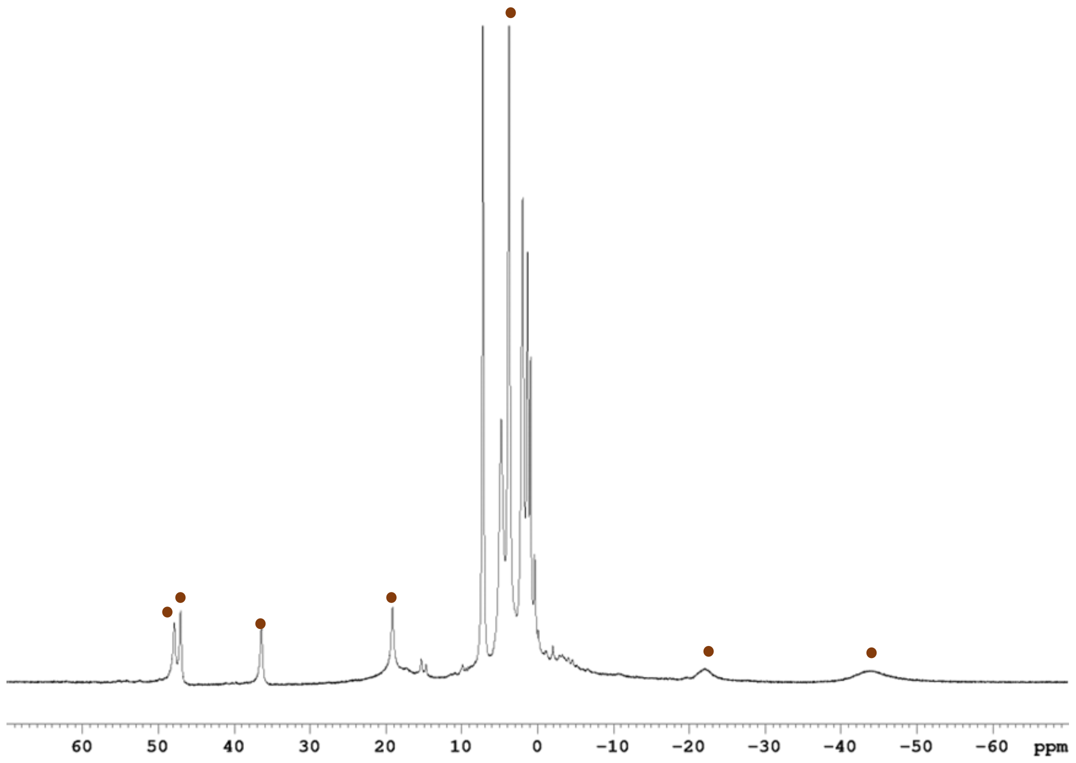

Figure S15. ^{1}H NMR spectrum of reaction product of [(Cz^{tBu}(Pyr^{iPr})$_2$)$_2$Co] and $CoCl_2$ in C_6D_6. ● = Cz^{tBu}(Pyr^{iPr})$_2$CoCl.

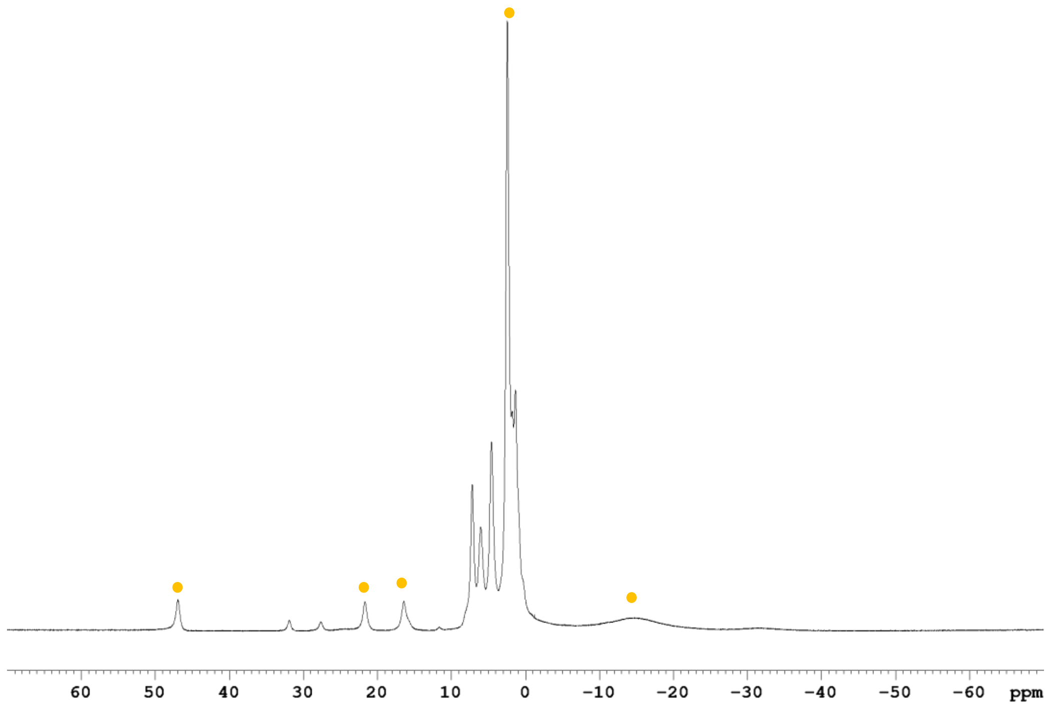

Figure S16. ^{1}H NMR spectrum of reaction product of [(Cz^{tBu}(Pyr^{iPr})$_2$)$_2$Fe] and $FeCl_2$ in C_6D_6. ● = Cz^{tBu}(Pyr^{iPr})$_2$FeCl.

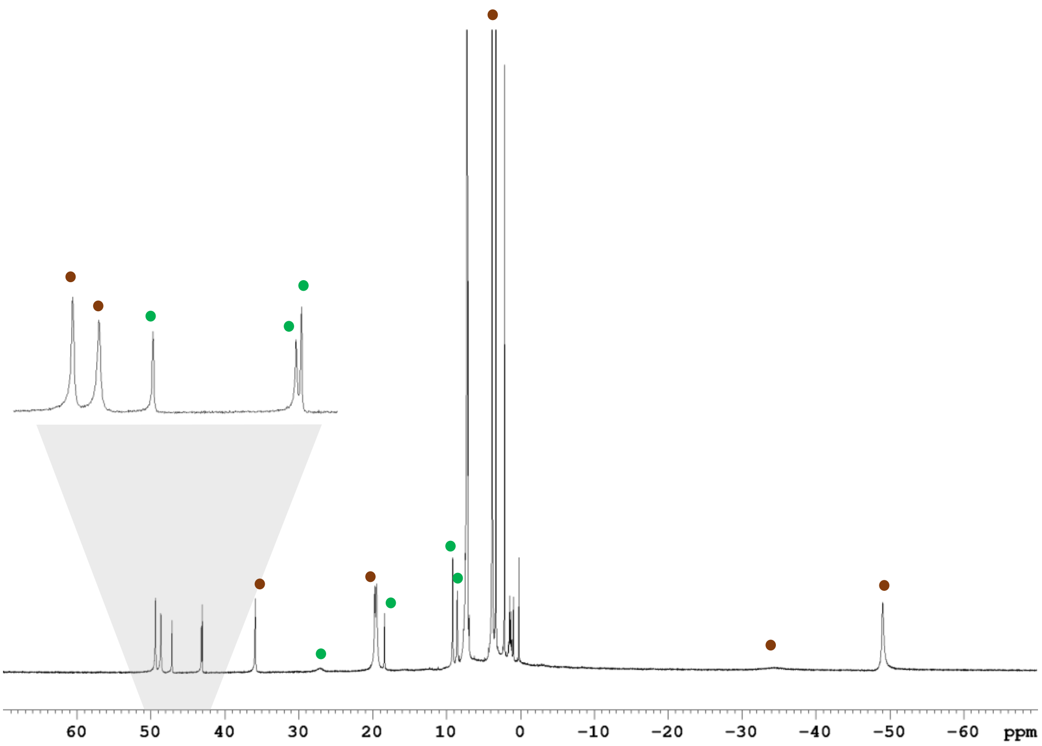

Figure S17. ^{1}H NMR spectrum of reaction product of [(Cz^{tBu}(Pyr^{iPr})$_2$)$_2$Ni] and $CoCl_2$ in C_6D_6. ● = Cz^{tBu}(Pyr^{iPr})$_2$NiCl; ● = Cz^{tBu}(Pyr^{iPr})$_2$CoCl.

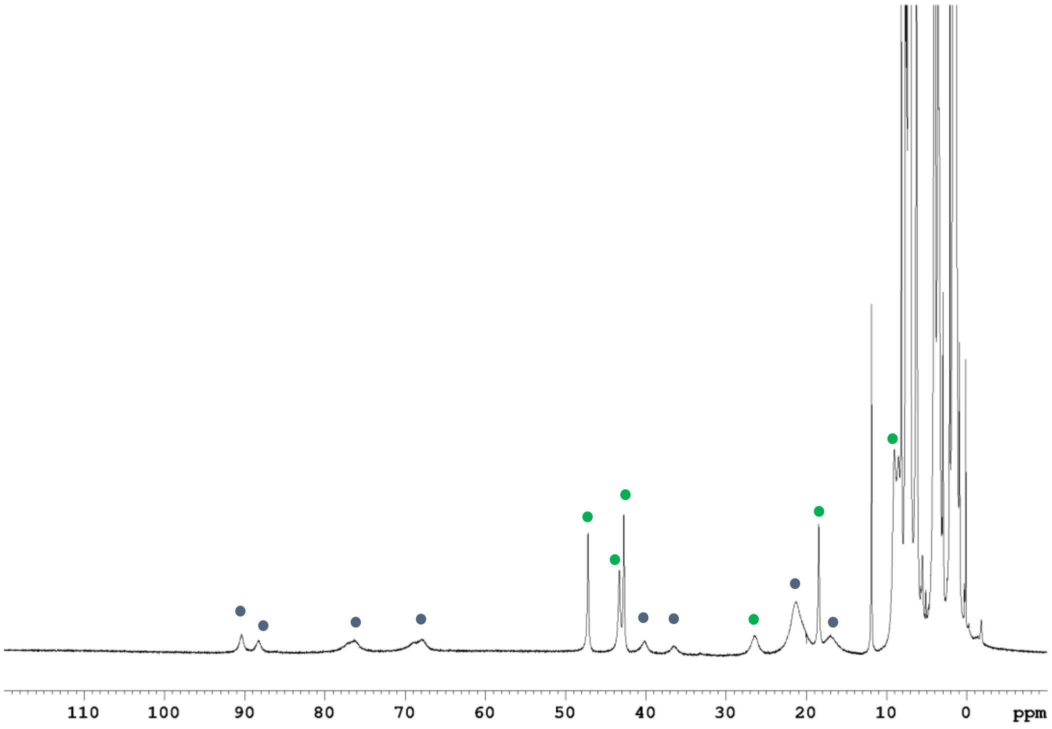

Figure S18. ^{1}H NMR spectrum of reaction product of [(Cz^{tBu}(Pyr^{iPr})$_2$)$_2$Ni] and $FeCl_3$ in C_6D_6. ● = Cz^{tBu}(Pyr^{iPr})$_2$NiCl; ● = Cz^{tBu}(Pyr^{iPr})$_2$FeCl$_2$.

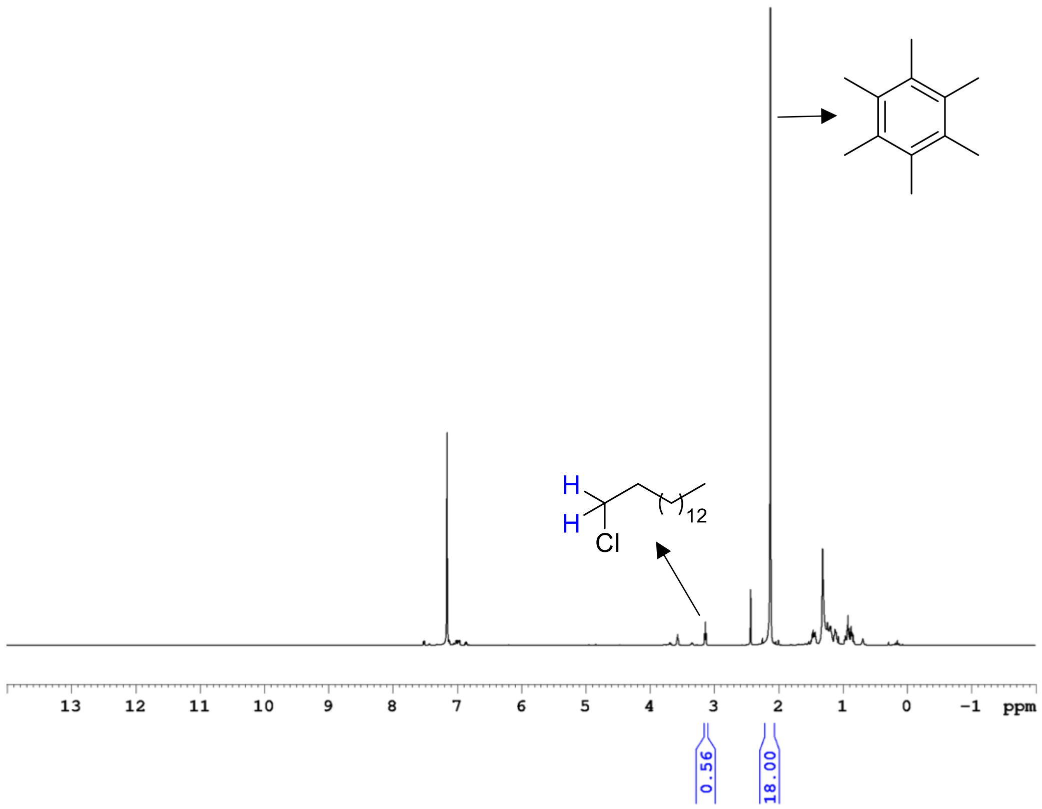

Figure S19. ^{1}H NMR spectrum of hydrodehalogenation reaction of entry 1.

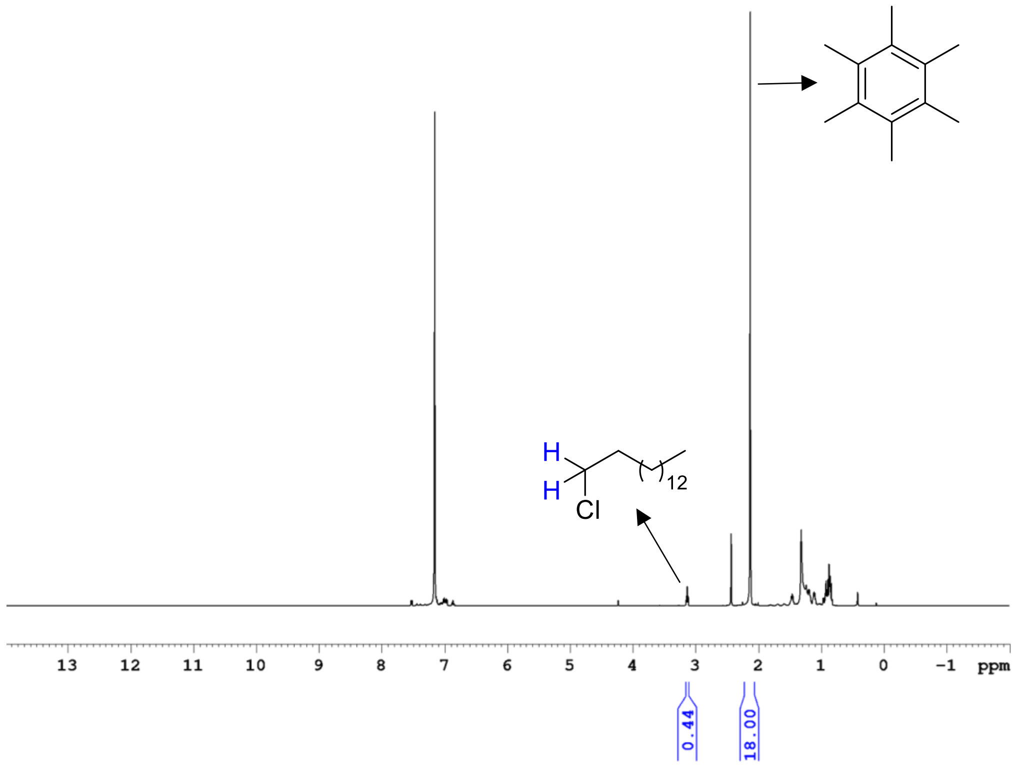

Figure S20. ^{1}H NMR spectrum of hydrodehalogenation reaction of entry 2.

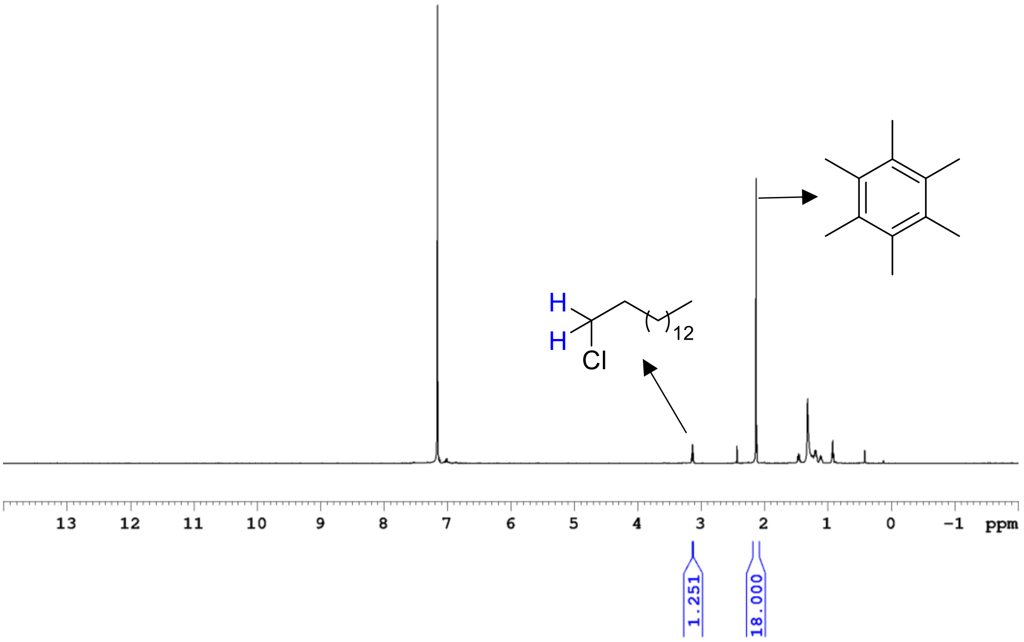

Figure S21. ¹H NMR spectrum of hydrodehalogenation reaction of entry 3.

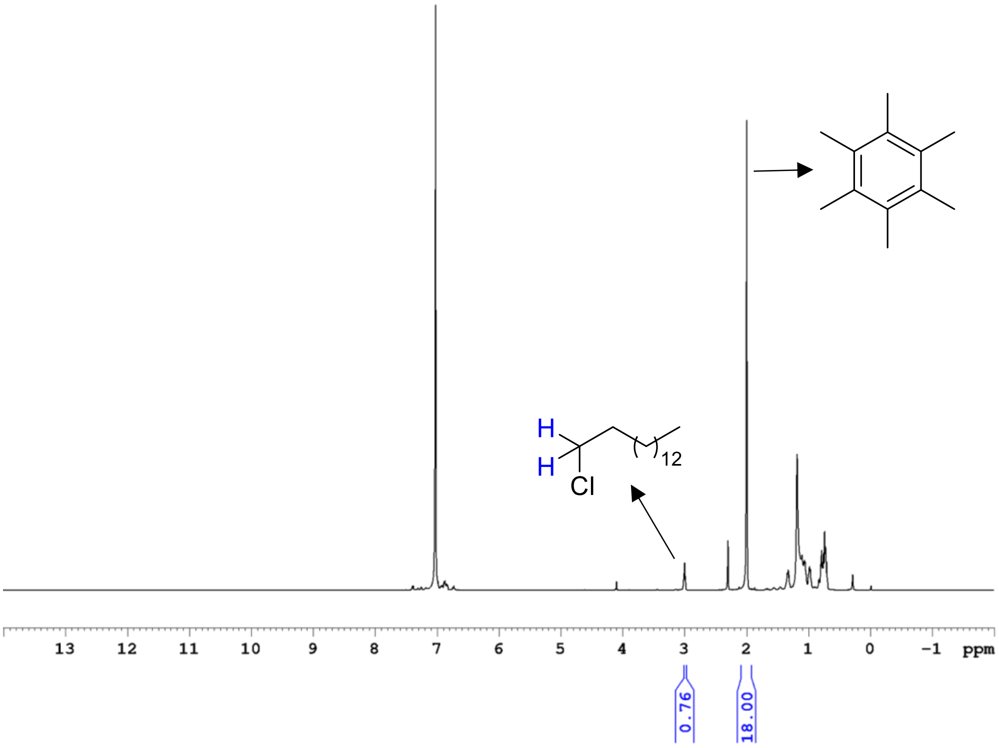

Figure S22. ¹H NMR spectrum of hydrodehalogenation reaction of entry 4.

(All were calculated at the UM06-D3/def2-SVP level)

Table S4. Cartesian coordinates (Å) of all optimized structure.

3 (G = -4223.335897 Hartree)

Atomic Number	Coordinates (Angstroms) X	Y	Z
Ni	0.00658600	0.00954200	-0.02397300
N	0.16535000	1.70152200	-1.48064300
N	1.09207100	2.65444100	-1.20307800
N	1.97649300	0.01960900	-0.03288700
N	1.13287600	-2.62809600	1.13597600
N	0.19817100	-1.68889200	1.43358500
C	0.62305400	3.88614000	-1.52255100
H	1.22121300	4.77927600	-1.35427700
C	-0.63366300	3.72817600	-2.06055000
H	-1.29983700	4.50423400	-2.43221500
C	-0.87265700	2.33662100	-2.02999400
C	-2.05663500	1.62202100	-2.57206800
H	-1.85936400	0.54505000	-2.65390600
C	0.68061300	-3.86945800	1.44244700
H	1.28799800	-4.75275400	1.25560400
C	-0.57328500	-3.73284900	1.99190200
H	-1.22869100	-4.52109200	2.35702400
C	-0.82784400	-2.34320500	1.98203600
C	-2.01535000	-1.65004400	2.54364400
H	-2.91512000	-1.80649200	1.92532900

C	2.78530000	1.03910900	-0.42998300
C	2.44469900	2.30656000	-0.93228800
C	3.46721300	3.20825500	-1.22632800
H	3.19304700	4.17456000	-1.65200200
C	4.82969900	2.90739400	-1.04246000
C	5.16574500	1.63363300	-0.58082700
H	6.21548600	1.34851400	-0.44571800
C	4.16543700	0.70919000	-0.28587900
C	4.17487700	-0.64142900	0.20999900
C	5.19536800	-1.55110000	0.50110600
H	6.23523500	-1.24472500	0.35758500
C	4.87776700	-2.82473700	0.96433100
C	3.51539400	-3.14289500	1.15458400
H	3.26391000	-4.11635400	1.58473400
C	2.47978000	-2.26183000	0.86415700
C	2.80292900	-0.98729600	0.35958300
C	5.93585700	3.91580100	-1.35947500
C	6.84810300	3.34961700	-2.45172400
H	7.65524800	4.06309400	-2.69418000
H	6.27794400	3.15115500	-3.37500800
H	7.32175800	2.40355000	-2.14386000
C	6.75710700	4.18113000	-0.09396700
H	7.22607300	3.26231400	0.29346300
H	6.12061900	4.59294300	0.70759900
H	7.56386000	4.90708500	-0.29756100
C	5.38302500	5.25049700	-1.85069400
H	6.21303000	5.94675100	-2.05664500
H	4.73191500	5.72795700	-1.09881200
H	4.80823800	5.14038700	-2.78580800

C	5.93379500	-3.87595100	1.30571800
C	7.34651100	-3.37270600	1.02593400
H	7.59452400	-2.48628900	1.63324600
H	7.48554200	-3.11087700	-0.03636100
H	8.08074200	-4.15762900	1.27305400
C	5.84408200	-4.23631900	2.79199700
H	6.60805700	-4.98835900	3.05653300
H	4.86082000	-4.65569600	3.06091000
H	6.00874700	-3.34551800	3.42134200
C	5.70190900	-5.13149000	0.45938500
H	5.76534100	-4.89626500	-0.61658300
H	4.71206900	-5.58012000	0.64404500
H	6.46112300	-5.90023400	0.68683300
H	-2.94980300	1.76388200	-1.94077000
H	-2.29798800	2.01557700	-3.57195000
H	-2.23475200	-2.04964800	3.54622400
H	-1.83564400	-0.57008600	2.62533000
N	-0.17141300	-1.51739600	-1.65080900
N	-1.09522900	-2.49511100	-1.46492400
N	-1.96328200	-0.00083400	-0.00932100
N	-1.10336100	2.50748200	1.42484200
N	-0.16565900	1.54249200	1.60891300
C	-0.63102700	-3.68539900	-1.91973200
H	-1.22763900	-4.59178500	-1.84022300
C	0.61898500	-3.47010600	-2.45345100
H	1.27998800	-4.20165200	-2.91400000
C	0.85926400	-2.08958700	-2.27762300
C	2.03686800	-1.32043800	-2.75508100
H	2.93779400	-1.52841700	-2.15384500

C	-0.64642100	3.70938300	1.85621400
H	-1.25550200	4.60716200	1.77221800
C	0.61417100	3.51578800	2.37220800
H	1.27467000	4.26103700	2.81100500
C	0.86761400	2.13527600	2.21107000
C	2.06156500	1.38695600	2.68068000
H	2.95373600	1.60938100	2.07163500
C	-2.77765700	-0.97264400	-0.50290000
C	-2.44416800	-2.17900200	-1.14183300
C	-3.47085000	-3.04479500	-1.51780900
H	-3.20263800	-3.95992800	-2.04762800
C	-4.83075000	-2.76618000	-1.28595400
C	-5.16018000	-1.54925000	-0.68674800
H	-6.20797200	-1.28089900	-0.50891900
C	-4.15567100	-0.66091800	-0.30721000
C	-4.15802700	0.62899600	0.33018700
C	-5.17419300	1.50169700	0.72952200
H	-6.21597500	1.21170900	0.56710300
C	-4.84997900	2.71877000	1.32215400
C	-3.48511700	3.01571700	1.52825000
H	-3.22761200	3.93781400	2.05676500
C	-2.45381000	2.17143000	1.13240100
C	-2.78409600	0.95784100	0.49870500
C	-5.94144400	-3.73565900	-1.69515500
C	-6.86674600	-3.05660900	-2.70929000
H	-7.67732500	-3.74061200	-3.01656900
H	-6.30792100	-2.76028700	-3.61307600
H	-7.33590100	-2.14914500	-2.29628200
C	-6.74714900	-4.13518400	-0.45516400

H	-7.21064700	-3.26331400	0.03398500
H	-6.10107300	-4.62987000	0.28987800
H	-7.55686400	-4.83574400	-0.72514100
C	-5.39566200	-5.00989000	-2.33295700
H	-6.22863900	-5.68067600	-2.60182700
H	-4.73563800	-5.56454000	-1.64450200
H	-4.83240900	-4.80014600	-3.25799600
C	-5.90095500	3.72677800	1.78708300
C	-7.31739200	3.25539000	1.47275800
H	-7.55840500	2.30896500	1.98487900
H	-7.46984600	3.10852700	0.39037900
H	-8.04795300	4.00890900	1.81151500
C	-5.79246400	3.92628300	3.30212800
H	-6.55257400	4.64523000	3.65504400
H	-4.80564000	4.31516800	3.60203400
H	-5.94986000	2.97320800	3.83466000
C	-5.67879200	5.06573400	1.07696500
H	-5.75581700	4.94681800	-0.01710400
H	-4.68641500	5.49270100	1.29614200
H	-6.43457500	5.80527200	1.39474100
H	2.26533600	-1.60474100	-3.79425300
H	1.83937800	-0.24100700	-2.71888400
H	2.29349900	1.67717600	3.71748700
H	1.88231100	0.30431600	2.64857600

$NiBr_2$ (G = -6655.619959 Hartree)

Atomic Number	Coordinates (Angstroms) X	Y	Z
Ni	0.00000000	0.86937000	0.00000000
Br	1.78904300	-0.34774300	0.00000000
Br	-1.78904300	-0.34775300	0.00000000

3[TS1] (G = -10878.953610 Hartree)

Atomic Number	Coordinates (Angstroms) X	Y	Z
Ni	-0.15926200	-1.86532500	0.03058600
N	-0.70026700	-2.95945100	1.51062600
N	-1.96486500	-3.04473300	2.08118300
N	-1.83908300	-1.07600200	-0.19926200
N	-0.16435100	0.77031600	-1.90329700
N	0.77437000	1.58633800	-1.27108800
C	-1.89885100	-3.68702500	3.28217900
H	-2.76986800	-3.82227900	3.90258000
C	-0.58592600	-4.06076700	3.48632100
H	-0.18281300	-4.59138300	4.33422800
C	0.13405400	-3.60170100	2.37124200
C	1.59884400	-3.73243000	2.13828800
C	0.46101400	-0.12529600	-2.71581500
H	-0.11948500	-0.85630700	-3.26490000

C	1.81442100	0.10479500	-2.63724200
H	2.60989300	-0.43905800	-3.12459000
C	1.98764500	1.14931400	-1.71798100
C	3.30872200	1.69919500	-1.29735700
C	-2.97770000	-1.56807100	0.41820500
C	-3.10386200	-2.50305300	1.44391800
C	-4.37823500	-2.86404300	1.88641800
H	-4.46400100	-3.62792300	2.65098400
C	-5.54531500	-2.28844200	1.37154600
C	-5.41799600	-1.30870100	0.38150100
H	-6.29972800	-0.81957800	-0.02759800
C	-4.16080700	-0.96049600	-0.08668300
C	-3.70687900	0.02359000	-1.03662300
C	-4.38319300	1.00862600	-1.74354100
H	-5.46780400	1.03683700	-1.69429900
C	-3.68563400	1.94669200	-2.50021200
C	-2.27450500	1.86175600	-2.50266200
H	-1.68303800	2.60748800	-3.02739400
C	-1.58864100	0.86605100	-1.80921900
C	-2.29006800	-0.07310900	-1.05391900
C	-6.93764600	-2.69327600	1.86363000
C	-7.75615300	-3.24084200	0.67887800
H	-8.75967500	-3.53223600	1.02100200
H	-7.25828900	-4.12106300	0.25039400
H	-7.86587300	-2.48570900	-0.10987700
C	-7.64594100	-1.46140000	2.45890400
H	-7.75697800	-0.66644000	1.71060700
H	-7.06822600	-1.06428700	3.30432700
H	-8.64753200	-1.74271800	2.81481800

C	-6.88618500	-3.78018500	2.94716300
H	-7.91192300	-4.02776600	3.25234900
H	-6.34319400	-3.42978000	3.83622800
H	-6.41274200	-4.69717400	2.56914100
C	-4.43119700	3.06048400	-3.25486500
C	-5.94961000	2.82801900	-3.23123100
H	-6.34796400	2.90974500	-2.21076300
H	-6.21013800	1.84327800	-3.64517900
H	-6.43481300	3.60027100	-3.84342000
C	-4.16276000	4.43930200	-2.62819000
H	-4.70857500	5.20056500	-3.20688900
H	-3.09917600	4.68498500	-2.65075200
H	-4.50679100	4.46495400	-1.58535100
C	-3.96263100	3.08707800	-4.72234600
H	-4.09692300	2.10136500	-5.18860900
H	-2.90641100	3.37674800	-4.79660100
H	-4.55405700	3.82586300	-5.28187500
N	0.20455300	-2.98390400	-1.49657100
N	1.42960300	-3.18573800	-2.13246000
N	1.61389400	-1.35916000	0.22913200
N	0.15315700	0.47601100	2.12006400
N	-0.53158100	1.47065600	1.42461300
C	1.24012800	-3.80810300	-3.33176600
H	2.06147300	-4.03205900	-3.99247600
C	-0.11041000	-4.04589900	-3.47445300
H	-0.60479100	-4.52803900	-4.30269000
C	-0.72685600	-3.53099800	-2.32232800
C	-2.18366100	-3.54433400	-2.01845400
C	-0.67778400	-0.16647600	2.99225800

H	-0.30837000	-0.96145900	3.62663300
C	-1.92436300	0.41977000	2.86682600
H	-2.83276200	0.16072100	3.38979600
C	-1.78485000	1.44245800	1.89753000
C	-2.83470800	2.41893300	1.48546600
C	2.67006600	-1.90097400	-0.47089900
C	2.65386500	-2.77040600	-1.55668800
C	3.86601000	-3.17715400	-2.11983600
H	3.84978300	-3.88323200	-2.94205800
C	5.10249700	-2.71557200	-1.65384400
C	5.11736500	-1.81803200	-0.57819400
H	6.05942200	-1.42940600	-0.19654100
C	3.92619100	-1.42621900	0.01015600
C	3.60771800	-0.50768600	1.08033400
C	4.37867400	0.35540600	1.86034000
H	5.46184300	0.34320200	1.76061800
C	3.75976600	1.26135800	2.72166800
C	2.35262700	1.26807200	2.77425500
H	1.82512700	1.98897100	3.38772100
C	1.57195800	0.39992400	2.02065300
C	2.18909800	-0.48909600	1.15133400
C	6.42531600	-3.16400200	-2.28178800
C	7.28817900	-3.85656300	-1.20979100
H	8.24282600	-4.17896400	-1.64989500
H	6.76452000	-4.73716400	-0.81417400
H	7.50436100	-3.17728000	-0.37553000
C	7.17011300	-1.93265700	-2.83217000
H	7.38801800	-1.21064500	-2.03502300
H	6.56140900	-1.43245500	-3.59749800

H	8.12228900	-2.24422900	-3.28514800
C	6.22351800	-4.15285400	-3.43931200
H	7.20606000	-4.43663600	-3.84014700
H	5.64324400	-3.69847000	-4.25484100
H	5.71861600	-5.06734800	-3.09776300
C	4.57268600	2.32579900	3.46750200
C	5.97347700	1.81854700	3.84695300
H	6.59390500	1.63369700	2.95959800
H	5.91025100	0.89459200	4.43963300
H	6.47930000	2.58858700	4.44535800
C	4.71985200	3.56621900	2.54852400
H	5.27430800	4.34638000	3.09216700
H	3.74866100	3.96919000	2.18637800
H	5.27793600	3.29019200	1.63914200
C	3.84977100	2.74618400	4.76154300
H	3.66365200	1.87457400	5.40472300
H	2.89567100	3.24336500	4.54539500
H	4.47756600	3.46291300	5.30825900
Br	2.16261500	4.04217500	0.88429600
Br	-0.99236700	4.81598100	-0.71492900
Ni	0.46944600	3.19236500	-0.28652900
H	-2.79316100	2.63751100	0.41517000
H	-2.68641500	3.36879500	2.02161500
H	-3.82612300	2.01688400	1.72597000
H	3.39212000	1.72809200	-0.20249000
H	3.43323800	2.72577600	-1.66589700
H	4.09132900	1.05606000	-1.71465500
H	-2.37555600	-3.87928500	-0.99153800
H	-2.69176900	-4.22471800	-2.71021200

H	-2.61949400	-2.54376000	-2.12973800
H	1.81581100	-4.06193000	1.11434500
H	2.01554100	-4.46318700	2.83949300
H	2.10720700	-2.77077100	2.28896600

3^Int (G = -10879.05726 Hartree)

Atomic Number	Coordinates (Angstroms) X	Y	Z
Ni	0.20029200	0.86371700	0.57682100
N	2.21960500	0.58489600	0.64374600
N	2.90682100	-0.14052200	1.55810200
N	-0.06694600	-0.34050300	2.05731700
N	-2.75647400	0.89483700	1.60770500
N	-2.96789500	2.18132500	1.95563200
C	4.22087100	0.18566900	1.52035500
H	4.93640300	-0.27042100	2.19954700
C	4.39368100	1.13520300	0.53635200
H	5.32119700	1.61669300	0.23406100
C	3.11076300	1.35954800	0.00977800
C	2.73047200	2.31145700	-1.06443600
C	-3.08577800	0.69405300	0.30636800
H	-3.00565300	-0.30278000	-0.13066200
C	-3.50524400	1.90199500	-0.20431000
H	-3.82333200	2.12524600	-1.22189600
C	-3.42473000	2.81121000	0.87281500
C	-3.80981000	4.24231800	0.90496800

C	0.91016200	-1.10375900	2.63517700
C	2.29440200	-1.09501000	2.41797600
C	3.09596100	-1.99814500	3.12309000
H	4.16543000	-2.01815600	2.90909300
C	2.58077600	-2.87622000	4.08791300
C	1.20608000	-2.83693300	4.34757600
H	0.76478500	-3.48561300	5.11235300
C	0.38251400	-1.97557300	3.63239000
C	-1.02632700	-1.66736200	3.69683800
C	-2.06160400	-2.05263600	4.55447900
H	-1.85761700	-2.80294100	5.32451900
C	-3.31421000	-1.44106200	4.47254600
C	-3.51148800	-0.47451800	3.46618800
H	-4.46337600	0.05877800	3.37841500
C	-2.50185400	-0.10250800	2.59126700
C	-1.23027100	-0.67169500	2.71088000
C	3.46896000	-3.85387100	4.85844200
C	2.99256600	-5.28616500	4.59819000
H	3.62063500	-6.00947000	5.14660400
H	3.04866700	-5.53065200	3.52392200
H	1.95037400	-5.44015400	4.92091400
C	3.37922000	-3.54778800	6.35665900
H	2.35051300	-3.64543800	6.73904700
H	3.71781900	-2.51966200	6.56949700
H	4.01448400	-4.24261900	6.93284600
C	4.93389400	-3.75923500	4.44277600
H	5.53364300	-4.48202000	5.02001700
H	5.35454800	-2.75790500	4.63694800
H	5.07630600	-3.99504100	3.37444300

C	-4.42613200	-1.68875800	5.49017700
C	-4.21201100	-2.99330100	6.25255200
H	-3.29586200	-2.97251900	6.86558800
H	-4.15031300	-3.85924700	5.57077000
H	-5.05361300	-3.16666300	6.94337700
C	-4.42360200	-0.52163700	6.48602600
H	-5.23492000	-0.64123600	7.22527000
H	-4.56674800	0.44930300	5.97927600
H	-3.46765500	-0.47689700	7.03628000
C	-5.79149800	-1.75243900	4.80128700
H	-5.80944200	-2.52477800	4.01313200
H	-6.07534400	-0.78894400	4.34831800
H	-6.57419000	-1.99945600	5.53816000
N	-0.21502300	-0.00204200	-1.22254000
N	-0.74209600	0.60793100	-2.31111700
N	-0.28882000	2.60549800	-0.08814100
N	0.10621800	3.01888800	2.85001000
N	-0.80258100	2.53126300	3.72017800
C	-1.21616600	-0.31665500	-3.18006300
H	-1.70336000	-0.02461600	-4.10667500
C	-0.96906500	-1.56396300	-2.64825500
H	-1.21473000	-2.53103600	-3.08175100
C	-0.33908200	-1.32231800	-1.41609300
C	0.12023200	-2.32835900	-0.42536200
C	1.34869200	2.56728100	3.15682500
H	2.20906500	2.88960100	2.56767600
C	1.22898000	1.74419500	4.25431900
H	2.01439600	1.18610600	4.76248100
C	-0.14365000	1.75730500	4.58346800

C	-0.82960800	1.08843600	5.71467900
C	-0.59699300	2.88963300	-1.39039800
C	-0.77418600	2.02112600	-2.47561000
C	-1.06302300	2.55916700	-3.73347800
H	-1.14691500	1.87793000	-4.58131100
C	-1.24498300	3.93341400	-3.94685000
C	-1.13173500	4.79147700	-2.84705400
H	-1.29290300	5.86902700	-2.96291000
C	-0.80621000	4.28521500	-1.59425600
C	-0.66641900	4.88299600	-0.28789400
C	-0.89257700	6.16780300	0.21588800
H	-1.16869500	6.96623400	-0.47953500
C	-0.83202400	6.41111900	1.58935400
C	-0.48019000	5.33906100	2.43409100
H	-0.44857700	5.46694100	3.52086800
C	-0.22678600	4.06630300	1.94535400
C	-0.34615500	3.80794600	0.57610500
C	-1.56604900	4.51006800	-5.32620400
C	-0.46830600	5.49900600	-5.73032700
H	-0.68162600	5.93086400	-6.72351000
H	0.51367000	4.99871100	-5.77918000
H	-0.38145600	6.33404000	-5.01671000
C	-2.91446800	5.23477500	-5.27179700
H	-2.91038600	6.06477600	-4.54718400
H	-3.72237200	4.54223800	-4.98083800
H	-3.16691000	5.65818800	-6.25938200
C	-1.65128000	3.43156400	-6.40199600
H	-1.87901700	3.89349200	-7.37674000
H	-2.45145600	2.70135500	-6.19278100

H	-0.70042400	2.88337900	-6.51416500
C	-1.23665200	7.74417500	2.21580000
C	-1.25121500	8.87039900	1.18626000
H	-2.00764600	8.70712700	0.40084600
H	-0.26847800	8.99120500	0.69829200
H	-1.50297100	9.82457700	1.67793000
C	-2.64621500	7.58498000	2.80012500
H	-2.96858500	8.51872700	3.29324600
H	-2.69227900	6.77332300	3.54781300
H	-3.37645800	7.35241100	2.00562700
C	-0.27127800	8.13195100	3.33867100
H	0.76981000	8.17944800	2.97550900
H	-0.31024200	7.42711500	4.18469300
H	-0.53783600	9.12439000	3.73958700
Br	-2.34985400	4.32897700	5.54949500
Br	-5.00527400	2.93348100	4.01768700
Ni	-2.70283200	2.93783200	3.72602400
H	-1.80531000	0.68108800	5.40666300
H	-1.03109000	1.82165500	6.51390800
H	-0.21419600	0.26934300	6.11568400
H	-3.12627700	4.82576600	1.54125700
H	-4.81593700	4.34691200	1.34488700
H	-3.80728500	4.67148700	-0.10820000
H	1.13841500	-2.11094300	-0.06710500
H	0.11820800	-3.32959700	-0.87761100
H	-0.53424700	-2.35235700	0.46243600
H	2.06019100	1.84516800	-1.80292400
H	3.62902100	2.66680100	-1.58731100
H	2.20074500	3.19049800	-0.65958000

3^{TS2} (G = -10878.953101 Hartree)

Atomic Number	Coordinates (Angstroms) X	Y	Z
Br	-1.03201300	0.11692300	-2.10022000
Br	2.75776200	-2.90797800	-2.25331500
Ni	-0.52094400	0.25271600	0.69873100
Ni	1.21282000	-1.09092400	-1.75237800
N	1.97559800	-0.02886500	-0.11960300
N	-0.10001100	-0.75322500	2.39353100
N	-0.39810500	2.14533000	1.52071200
N	-2.49067600	0.23105600	0.68875500
N	0.84894800	-1.72930800	2.34224900
N	-1.00819700	-2.79999700	0.32138000
N	1.73420200	0.08103000	-3.27421400
N	-2.02511800	-2.62910800	-0.53938100
N	-1.47356800	2.95690300	1.68928100
N	1.99036300	1.36314700	-2.94280100
C	0.47098800	-2.77572400	3.11298400
C	-0.74033400	-2.47735200	3.68530900
C	-1.06462000	-1.19287400	3.21047700
C	-3.40755400	-0.71529800	0.28773700
C	-3.19748700	1.39223300	0.84229500
C	2.92469200	0.82220500	-0.70712800
C	2.64479100	-0.59842800	0.96938500
C	-3.26443100	-2.03487000	-0.19077400
C	-4.72825600	-0.18380000	0.28572600

C	-2.77574600	2.68804800	1.18975700
C	-4.58522700	1.20753800	0.59538700
C	-0.11316400	-3.54853000	-0.33577200
C	0.59752600	2.69293500	2.23918400
C	1.18456600	0.08206700	-4.48566000
C	2.92393400	1.58917200	-1.89688000
C	4.14024600	0.83110300	0.01521500
C	2.20987400	-1.39165800	2.05845400
C	3.98359600	-0.13433900	1.05829400
C	-4.40346000	-2.77863100	-0.48568100
C	-5.85588200	-0.94124900	-0.04252400
C	-3.69238700	3.73682000	1.14122000
C	-5.50040900	2.25936500	0.62158700
C	-0.55305200	-3.81511000	-1.65581600
C	-1.78596100	-3.21339000	-1.74118800
C	0.15451900	3.86941800	2.86206900
C	-1.16520100	3.99371700	2.50263500
C	1.10906800	1.40709400	-4.96407100
C	1.62985300	2.18779600	-3.95381100
C	3.98352400	2.45004500	-2.16428600
C	5.20205200	1.69448900	-0.26576800
C	3.13289500	-1.77516000	3.03492300
C	4.89172600	-0.53353400	2.03702600
C	-5.71523400	-2.27321400	-0.40884900
H	-4.24038500	-3.81037300	-0.81448200
H	-6.83788800	-0.46044300	-0.01508100
C	-5.05911800	3.55707300	0.86466100
H	-3.32683900	4.75082300	1.32398700
H	-6.55349600	2.04834800	0.41699600
H	0.73595500	4.52041400	3.51106100

H	-1.91851500	4.71947700	2.79598200
H	-1.33391200	-3.09852400	4.35206900
H	1.11384500	-3.64724300	3.21675100
C	5.11739000	2.56885000	-1.34075500
C	4.48703300	-1.40712900	3.03581100
H	2.75480400	-2.35214900	3.88261700
H	5.90937300	-0.13535900	2.00373200
H	-0.01741500	-4.37433600	-2.42064000
H	-2.50253300	-3.11893900	-2.55605100
H	3.95536400	3.00161600	-3.10888800
H	6.08869700	1.66378800	0.37325500
H	0.69896200	1.74054300	-5.91484200
H	1.78148700	3.26338200	-3.87872700
C	5.40787800	-1.91927800	4.13938200
C	5.44241800	-3.44983000	4.09724400
H	6.11282900	-3.84416200	4.88045300
H	4.44540500	-3.89052900	4.26139500
H	5.80817900	-3.80850900	3.12057700
C	4.89171700	-1.45282000	5.50399500
H	5.55787800	-1.80639000	6.30995000
H	4.85117300	-0.35146400	5.55343300
H	3.88092900	-1.83447100	5.72203000
C	6.83272100	-1.40335800	3.96598900
H	7.26730500	-1.71723800	3.00230500
H	6.88162700	-0.30304200	4.02369500
H	7.47674000	-1.80361200	4.76643200
C	6.21255800	3.56682100	-1.70615100
C	7.34821600	3.55469000	-0.68797700
H	6.99218500	3.80463300	0.32566700
H	7.85101300	2.57425500	-0.64426600

H	8.10986500	4.30177100	-0.96644200
C	5.61829000	4.97758800	-1.74566900
H	4.81273100	5.06653000	-2.49280800
H	5.19562300	5.25473400	-0.76505800
H	6.39463000	5.71819800	-2.00491100
C	6.79561700	3.21512200	-3.07835900
H	6.03777400	3.25641600	-3.87745600
H	7.59932900	3.92189000	-3.34853500
H	7.22105500	2.19764400	-3.07535500
C	-6.89099900	-3.18342400	-0.75463200
C	-8.22574700	-2.45686300	-0.62436600
H	-8.39372600	-2.08954100	0.40200600
H	-8.29410000	-1.59732200	-1.31208400
H	-9.05318100	-3.14313200	-0.87064300
C	-6.75428700	-3.67607500	-2.19860400
H	-5.82858100	-4.25374800	-2.35445100
H	-7.60173300	-4.33064600	-2.46759300
H	-6.74091100	-2.82714000	-2.90293100
C	-6.90373500	-4.38332000	0.19710900
H	-5.97750900	-4.97661500	0.12708700
H	-7.00895100	-4.05226600	1.24417400
H	-7.74828000	-5.05536700	-0.03578200
C	-5.98007500	4.77605200	0.84010900
C	-7.42463300	4.38712100	0.54137100
H	-7.52382200	3.90367400	-0.44470500
H	-7.83120300	3.70100200	1.30314500
H	-8.06169800	5.28721200	0.53343800
C	-5.51289000	5.74812700	-0.24743100
H	-6.17240800	6.63267500	-0.28736000
H	-4.48616000	6.10815500	-0.07007700

H	-5.52834600	5.26421500	-1.23840500
C	-5.94968800	5.47668100	2.20205200
H	-4.94034100	5.83468600	2.46413100
H	-6.61994600	6.35396900	2.20295900
H	-6.28023600	4.79359700	3.00274300
C	0.67216600	-1.17450400	-5.08278300
H	-0.36390800	-1.35332300	-4.74210200
H	0.66625400	-1.12356800	-6.18068500
H	1.28362600	-2.03154600	-4.75773100
C	-2.25944500	-0.39475700	3.58282000
H	-3.14785400	-0.69103000	3.00215400
H	-2.48588700	-0.56037000	4.64719700
H	-2.09622500	0.67974000	3.42307500
C	1.06848600	-4.16952700	0.32871600
H	1.83467100	-3.44352000	0.64133500
H	0.74224900	-4.74236100	1.21237000
H	1.56615200	-4.85653500	-0.36788900
C	1.95865900	2.13028400	2.37276700
H	1.93938900	1.10231500	2.76523800
H	2.49050800	2.11871100	1.40979900
H	2.54271100	2.74798000	3.06863700

$Cz^{tBu}(Pyr^{Me})_2NiBr$ (G = -5439.512478 Hartree)

Atomic Number	Coordinates (Angstroms) X	Y	Z
Br	-3.12755900	-0.11972800	2.03989300
Ni	-1.99830300	-0.08615000	-0.02236900

N	-2.01748700	-2.08338900	-0.47932500
N	-0.94039300	-2.89948300	-0.57544800
N	-0.14786000	-0.02414300	-0.44353900
N	-1.13150800	2.79057600	-0.58548300
N	-2.15262400	1.90587100	-0.48650100
C	-1.33666800	-4.15627300	-0.89210100
H	-0.61427700	-4.95509300	-1.04019100
C	-2.71114100	-4.15529200	-0.99228300
H	-3.35912800	-4.99434300	-1.23646300
C	-3.09686700	-2.83033000	-0.72464800
C	-4.46133900	-2.24519500	-0.70732300
H	-4.56810000	-1.46036100	-1.47575900
C	-1.60791000	4.01602600	-0.91487600
H	-0.93765800	4.85859000	-1.06663600
C	-2.97849000	3.92361100	-1.02076000
H	-3.67941200	4.71582100	-1.27497800
C	-3.27731200	2.57764200	-0.74380000
C	-4.59924900	1.90145200	-0.73929300
H	-4.74770300	1.34634000	0.20227400
C	0.69822800	-1.08882900	-0.32353300
C	0.39118300	-2.45392300	-0.35185400
C	1.42741400	-3.37273600	-0.17530600
H	1.18220100	-4.43564500	-0.15641500
C	2.76507300	-2.97855500	-0.00425800
C	3.06305700	-1.61078100	0.00443300
H	4.09529500	-1.26975100	0.14057900
C	2.04590100	-0.67321000	-0.14258400
C	1.99579200	0.77245700	-0.14177400
C	2.95326100	1.77610900	0.00977200

H	4.00133600	1.49718100	0.14589700
C	2.56274000	3.11610800	0.00282500
C	1.19684700	3.41793900	-0.17188700
H	0.88765700	4.46661000	-0.15121500
C	0.22589400	2.43754800	-0.35264000
C	0.62630600	1.09373600	-0.32318900
C	3.89347700	-3.99467600	0.18376700
C	4.56518500	-3.76376300	1.54101700
H	5.38307700	-4.48860100	1.69727000
H	3.84042100	-3.88303900	2.36387700
H	4.99694600	-2.75326200	1.62242600
C	4.92483800	-3.82053100	-0.93534500
H	5.36761500	-2.81153300	-0.93739100
H	4.46402300	-3.98395900	-1.92424900
H	5.74964000	-4.54491400	-0.81840400
C	3.39227100	-5.43523200	0.14367600
H	4.23845300	-6.12889200	0.27937000
H	2.91947400	-5.68081800	-0.82245400
H	2.66707900	-5.64314600	0.94852000
C	3.55067500	4.26837700	0.19072400
C	4.98248500	3.76801500	0.35532400
H	5.32494000	3.20331700	-0.52795700
H	5.09253700	3.12143900	1.24178700
H	5.66531400	4.62396400	0.48515600
C	3.50869200	5.18897000	-1.03297800
H	4.22632200	6.01972600	-0.91731600
H	2.51224800	5.63468600	-1.18617900
H	3.77149200	4.63599100	-1.95057400
C	3.17873600	5.06440600	1.44561800

H	3.20162400	4.42021500	2.34055200
H	2.16994200	5.50335300	1.37832200
H	3.88998900	5.89391500	1.60267900
H	-5.21761100	-3.01621300	-0.90887200
H	-4.67171400	-1.78135500	0.27112500
H	-5.41057700	2.63330400	-0.85424000
H	-4.67983000	1.18076800	-1.57142600

----

4 (G = -4537.157719 Hartree)

Atomic Number	Coordinates (Angstroms) X	Y	Z
Ni	0.00636800	-0.00884700	-0.11715800
N	0.02588800	-0.02555400	2.17257400
N	1.26278900	-0.15032900	2.72362200
N	1.57550400	1.16369400	0.01675600
N	0.52094300	1.46413100	-2.80470500
N	0.24985400	0.20323600	-2.37535800
N	1.22689700	-1.93842500	-0.05343400
N	-1.56892900	-1.17196600	-0.25553300
N	-2.46531100	1.58449200	-1.12539100
N	-1.47995300	1.72489900	-0.19959300
C	-2.28658500	-0.71721500	2.79451400
H	-2.49975200	-0.52309300	1.73287800
C	-2.92404300	-2.03576400	3.21076300
H	-2.46651700	-2.89923600	2.69988900
H	-3.99918000	-2.03642100	2.96758700

H	-2.83532100	-2.19442600	4.30072200
C	-2.86466200	0.44268200	3.60145900
H	-2.44417400	1.40497200	3.27037000
H	-2.64325500	0.32743300	4.67735100
H	-3.95939400	0.49044600	3.48200900
C	-0.79663100	-0.73431100	2.95320000
C	-0.07277500	-1.36436400	3.99000700
H	-0.45848600	-2.02491800	4.76400500
C	1.23146500	-0.96898700	3.80330000
H	2.13862700	-1.20938300	4.35397500
C	2.32897200	0.71675500	2.36425600
C	3.31665800	1.02165300	3.29424400
H	3.24592500	0.57705300	4.29057500
C	4.38372300	1.90752500	3.02790500
C	4.41223200	2.54767800	1.79333600
H	5.20018900	3.26376700	1.54482600
C	3.42541400	2.26836600	0.84351100
C	2.39231400	1.32335500	1.09563800
C	2.05109400	2.00618500	-0.94327200
C	3.18790700	2.73402300	-0.49374600
C	3.80696800	3.69792500	-1.29484900
H	4.66510200	4.24643600	-0.89718200
C	3.31858700	3.95143500	-2.57222500
C	2.22610900	3.18393600	-3.03198200
H	1.88659400	3.32853300	-4.06102100
C	1.58601500	2.22566800	-2.25393500
C	-0.14473400	1.73723400	-3.95339300
H	-0.07186600	2.71308100	-4.42886200
C	-0.84409600	0.60737400	-4.30980700

H	-1.48728900	0.47553100	-5.17742700
C	-0.55247500	-0.33575600	-3.29953500
C	-0.93943000	-1.78308200	-3.29237400
H	-1.00310900	-2.09990600	-2.24158900
C	0.16696100	-2.60463600	-3.94875700
H	-0.08901700	-3.67674200	-3.93864100
H	1.12040400	-2.47720800	-3.41284300
H	0.32088300	-2.29915200	-4.99892700
C	-2.27713100	-2.02237300	-3.97632600
H	-2.59229300	-3.07047800	-3.84671600
H	-2.21038300	-1.83276500	-5.06291600
H	-3.07404100	-1.38084600	-3.56459000
C	5.44877700	2.11977900	4.10267200
C	6.13683500	0.78239900	4.39593900
H	6.93145500	0.90812000	5.15222100
H	6.59597400	0.37084600	3.48044800
H	5.42955400	0.02879700	4.77958200
C	4.80467900	2.65978200	5.38288500
H	5.56890100	2.82103800	6.16317400
H	4.05257800	1.96892300	5.79738000
H	4.30245000	3.62326900	5.19253000
C	6.51659300	3.11306300	3.65532300
H	7.26936100	3.23898900	4.45135200
H	6.08935000	4.10755200	3.44393900
H	7.04566600	2.76729900	2.75164600
C	3.90902700	5.01624500	-3.49538400
C	4.40175600	4.37166700	-4.79437400
H	4.83578300	5.13375000	-5.46499700
H	3.59087100	3.87063000	-5.34768800

H	5.17894300	3.61683200	-4.58706900
C	5.08772600	5.73741700	-2.84921300
H	5.48644400	6.49894400	-3.54006500
H	5.91147200	5.04435000	-2.60978200
H	4.79331700	6.25490700	-1.92098300
C	2.83091600	6.05763600	-3.81320700
H	3.23542100	6.85506800	-4.46102000
H	2.45512500	6.52540000	-2.88678300
H	1.96705000	5.61291200	-4.33408200
C	3.52235100	-1.22093700	-0.71079900
H	3.01503500	-0.29057900	-1.00618400
C	4.79015800	-0.88730200	0.06334000
H	4.57390000	-0.39888400	1.02803200
H	5.42715500	-0.20313400	-0.52066100
H	5.38495600	-1.79666800	0.26413100
C	3.85454400	-1.99182900	-1.98600500
H	2.94693200	-2.19445100	-2.57555300
H	4.33054900	-2.96058200	-1.75190300
H	4.54915700	-1.41476000	-2.61806600
C	2.55233900	-2.01699400	0.10816100
N	0.69713200	-2.92093100	0.72327800
C	2.87697400	-3.02802600	1.03934100
H	3.86532500	-3.31213900	1.39519700
C	1.66851600	-3.57312100	1.40716000
H	1.42717500	-4.37336400	2.10364400
C	-0.64841900	-3.35035900	0.56900600
C	-0.99064500	-4.66881200	0.87025100
H	-0.20193100	-5.33540900	1.22154800
C	-2.29068900	-5.18109800	0.70199400

C	-3.26164900	-4.34221900	0.15416600
H	-4.28178500	-4.70389900	-0.01699600
C	-2.94454500	-3.02411200	-0.16631000
C	-1.64358400	-2.49267900	0.07252200
C	-2.80249700	-0.82846500	-0.72306500
C	-3.70078800	-1.93095900	-0.71226300
C	-5.00465500	-1.82241900	-1.20259800
H	-5.65022200	-2.70479800	-1.19264400
C	-5.45495500	-0.60512200	-1.70362700
C	-4.57676000	0.49909400	-1.65612300
H	-4.95054200	1.47412800	-1.97985200
C	-3.27204400	0.41696400	-1.18180500
C	-2.72633000	2.76569000	-1.73689300
H	-3.46774000	2.83829200	-2.52984300
C	-1.91399800	3.71912600	-1.16778100
H	-1.85984900	4.77610100	-1.42019900
C	-1.16148000	3.02364500	-0.19546500
C	-0.23786100	3.62781800	0.81760100
H	0.51505400	2.86741400	1.06953000
C	-1.01896700	3.93349400	2.09289100
H	-0.35436900	4.36224400	2.86069900
H	-1.46931600	3.01723800	2.50538600
H	-1.83070300	4.65709700	1.89960100
C	0.45690500	4.87389700	0.28955200
H	1.21709400	5.22250300	1.00733800
H	-0.26105300	5.70146600	0.14538900
H	0.96535500	4.68943600	-0.67149000
C	-2.66354900	-6.60310300	1.12325700
C	-3.29673000	-7.35364500	-0.05116400

H	-3.57177700	-8.37994700	0.24842900
H	-2.59418600	-7.42214100	-0.89887900
H	-4.21249200	-6.86171400	-0.41592000
C	-1.45553000	-7.40548300	1.59785600
H	-1.77019700	-8.42357500	1.88151600
H	-0.98137200	-6.95235600	2.48474000
H	-0.69058500	-7.50375100	0.80892800
C	-3.66471200	-6.52020100	2.28098600
H	-3.94888500	-7.52925700	2.62849600
H	-4.58715300	-5.99326900	1.98704700
H	-3.22922500	-5.97269400	3.13490300
C	-6.84899800	-0.41141500	-2.29834700
C	-6.71344800	0.01676900	-3.76331700
H	-7.70699900	0.14161900	-4.22856200
H	-6.15634100	-0.74075300	-4.34129900
H	-6.17588900	0.97374000	-3.86632100
C	-7.61045200	0.66260000	-1.51588500
H	-8.62162400	0.80439800	-1.93576500
H	-7.10306700	1.64061300	-1.54387300
H	-7.71796200	0.37370200	-0.45676200
C	-7.66813200	-1.69753300	-2.25490600
H	-8.66476800	-1.52297200	-2.69354100
H	-7.81905100	-2.05306500	-1.22190800
H	-7.19077200	-2.50774200	-2.83092400

4^{TS1} (G = -11192.77269 Hartree)

Atomic Coordinates (Angstroms)

Number	X	Y	Z
Ni	0.08932800	0.38863500	-0.13551700
N	2.10571000	0.42037200	-0.04775800
N	0.15813500	1.19939500	1.79495600
N	0.24202400	2.32868000	-1.08098000
N	-2.05003700	0.37365300	0.05000600
Br	1.44400200	-0.73185500	-2.84311400
Ni	-0.46045700	-1.55172900	-1.88121600
N	-0.57166700	2.29455100	2.05856000
C	-0.09826800	2.93241300	3.15709100
C	0.96136600	2.20443000	3.63700300
C	1.08028200	1.11097900	2.76423700
Br	-1.58841900	-1.17442300	-3.85940200
N	0.31907400	-2.08558800	0.05568700
N	-1.76190100	-2.82824800	-1.55561500
C	2.82167400	1.58366100	-0.08801100
C	3.02027700	-0.55946900	-0.27091800
C	-3.20515300	-0.09256400	-0.61429100
C	-2.39871300	1.64908200	0.49331200
C	2.37690700	2.91332000	-0.10230000
C	4.21834500	1.34209500	-0.24133500
C	2.80143600	-1.93770000	-0.34587300
C	4.33329600	-0.07413800	-0.43395400
C	-0.74410600	3.03963800	-1.65099300
N	1.02181700	3.22378500	-0.41682300
C	-0.34783300	-2.53967300	1.16099800
N	1.62264200	-2.45089400	0.27287300
C	-1.57427200	-4.13012800	-1.78022100

N	-3.04298600	-2.62452500	-1.23390900
C	-3.68756800	-1.33578700	-1.15204600
C	-4.20065200	0.93934100	-0.65890100
C	-1.76058200	2.58506300	1.34799600
C	-3.66797500	2.05496000	0.02809700
C	3.32631600	3.93878200	-0.05459000
C	5.14552300	2.37909400	-0.21580400
C	3.84378600	-2.79218700	-0.65246700
C	5.38760200	-0.94422400	-0.73712800
C	-1.64797700	2.46726000	-2.70001700
C	-0.64773900	4.38881100	-1.26645800
C	0.48573000	4.46328200	-0.48715700
C	-1.77652800	-2.33995700	1.56881900
C	0.52627100	-3.21668100	2.02028200
C	1.75678900	-3.12506800	1.43084600
C	1.99049100	-0.06024600	2.96767600
C	-0.26296200	-4.68604300	-2.24731300
C	-2.78560700	-4.80644700	-1.55985000
C	-3.68231200	-3.82578200	-1.19996900
C	-4.99988000	-1.41570300	-1.65062300
C	-5.49656800	0.82265700	-1.14386500
C	-2.34568000	3.82697000	1.58566000
C	-4.25666600	3.29301500	0.28838800
C	4.71128600	3.69970100	-0.06889900
H	2.97420100	4.96988400	-0.07007400
H	6.21098700	2.14612600	-0.31347300
C	5.15430900	-2.31241500	-0.88412100
H	3.63335200	-3.86704600	-0.67090200
H	6.38930400	-0.52859900	-0.87130900

H	-2.07059200	1.50856200	-2.34397100
H	-2.33195100	-1.83102200	0.78849000
H	0.28801400	-3.67111900	2.97959500
H	2.74026000	-3.43542300	1.78121300
H	1.89495100	-0.71752100	2.09176500
H	1.55106000	2.41000900	4.52778100
H	-0.56078100	3.82293300	3.57092200
H	0.46175000	-3.85033900	-2.28582500
C	-5.94443200	-0.39291300	-1.63385200
C	-3.58566200	4.22475800	1.06592100
H	-1.80926200	4.53940700	2.21390200
H	-5.24358500	3.51360100	-0.12364400
C	5.73599500	4.83407700	-0.01060800
C	6.25308200	-3.31327700	-1.23910400
C	-7.40266900	-0.59744300	-2.03678600
C	-4.14956100	5.60102300	1.41354300
C	6.65702300	4.62015100	1.19567000
C	6.57072900	4.84183700	-1.29560100
C	5.08109000	6.20604300	0.13543300
C	5.87970600	-4.04023500	-2.53569800
C	6.40825200	-4.33505600	-0.10685800
C	7.60314600	-2.63207100	-1.44776900
C	-8.29613500	-0.25345400	-0.83823400
C	-7.76573700	0.30994600	-3.21470800
C	-7.70129800	-2.04073800	-2.43843000
C	-3.20349600	6.69900200	0.91792400
C	-4.31207500	5.71693400	2.93355800
C	-5.51457800	5.83490300	0.77098000
H	6.07793500	4.61583300	2.13522600

H	7.40970000	5.42615900	1.25981200
H	7.19972900	3.66256500	1.13309100
H	5.92794000	4.99335200	-2.17961700
H	7.31693000	5.65586700	-1.26952200
H	7.11795400	3.89575300	-1.43842000
H	4.47277200	6.27285000	1.05410400
H	5.85623400	6.98881000	0.19353200
H	4.43653600	6.44806800	-0.72708000
H	5.76384700	-3.32367900	-3.36645800
H	6.66320200	-4.76741500	-2.81403000
H	4.93099500	-4.59427900	-2.44011800
H	6.67824900	-3.83366400	0.83885100
H	7.20264900	-5.06399200	-0.34656000
H	5.48104900	-4.90647800	0.06727600
H	7.56623100	-1.89892700	-2.27129900
H	8.36845900	-3.38343200	-1.70638000
H	7.94493700	-2.11190900	-0.53663000
H	-8.05127500	-0.88897700	0.03030500
H	-9.35944900	-0.41147800	-1.09097200
H	-8.18358100	0.79766500	-0.52589100
H	-7.20292800	0.02320100	-4.11770600
H	-8.84183100	0.22549500	-3.44855700
H	-7.55098300	1.37042700	-3.00200800
H	-7.49753300	-2.74634400	-1.61367000
H	-8.76693400	-2.14388800	-2.70391800
H	-7.11376500	-2.35445900	-3.31835400
H	-3.13350300	6.68414600	-0.18274400
H	-3.57592100	7.69467800	1.21707800
H	-2.18337200	6.58999900	1.32423400

H	-4.98463800	4.93055300	3.31671500
H	-4.74130400	6.69771300	3.20443900
H	-3.34955300	5.62207900	3.46339500
H	-5.46212700	5.77252900	-0.32962300
H	-5.88551300	6.84125800	1.02879500
H	-6.26379900	5.10614900	1.12421000
H	-1.28810400	5.20991200	-1.57359200
H	0.97343500	5.33133700	-0.05003200
H	-5.29867900	-2.36273600	-2.09271600
H	-6.17265200	1.68115800	-1.08273300
H	-2.97751400	-5.87393000	-1.64784400
H	-4.73155200	-3.91268800	-0.93147700
C	0.25846600	-5.76680800	-1.30544000
H	-0.44790700	-6.61484400	-1.25609000
H	1.22565900	-6.15742900	-1.66243300
H	0.40263900	-5.40235200	-0.27487300
C	-0.42179700	-5.22117700	-3.66772100
H	-0.78298300	-4.42731000	-4.34223700
H	0.54407500	-5.58446000	-4.05498200
H	-1.14099200	-6.05858800	-3.69887000
C	-0.80093600	2.20105900	-3.94648300
H	-1.41954100	1.76308900	-4.74575200
H	-0.37250000	3.15070100	-4.31667400
H	0.02877300	1.50845400	-3.73818900
C	-2.78668600	3.41157000	-3.05782500
H	-2.39726300	4.37515900	-3.43226500
H	-3.38579700	2.97558000	-3.87242700
H	-3.45619600	3.61528600	-2.20575900
C	3.44474500	0.36740500	3.12148600

H	3.58125700	0.96568200	4.03991000
H	4.10146500	-0.51596200	3.19619300
H	3.78940300	0.97932200	2.27352500
C	1.54266000	-0.85761300	4.19163700
H	2.16008700	-1.76605400	4.30279000
H	1.64590400	-0.26390200	5.11707000
H	0.49020200	-1.16902500	4.10327500
C	-1.87105100	-1.42737600	2.79161300
H	-2.93068300	-1.22656600	3.02185700
H	-1.41794300	-1.89852000	3.68215300
H	-1.37673900	-0.45952400	2.61781400
C	-2.45564600	-3.67066700	1.88544200
H	-2.01870700	-4.14080600	2.78348700
H	-3.52751100	-3.50731000	2.08832700
H	-2.36879200	-4.39402000	1.06182100

4^{Int} (G = -11192.87057 Hartree)

Atomic Number	Coordinates (Angstroms) X	Y	Z
C	-4.66851500	-0.55012000	6.53651200
Ni	-0.07746100	0.86990800	0.66857300
N	1.96550100	0.67990300	0.76614000
N	2.65563400	-0.09025400	1.64322100
N	-0.29736200	-0.30342200	2.18626400
N	-3.01212200	0.81017000	1.68161300
N	-3.28226700	2.11973600	1.89333200

C	3.96328200	0.26271600	1.64550300
H	4.67288200	-0.21835400	2.31448800
C	4.13217700	1.27750100	0.72797800
H	5.05655200	1.79573400	0.48158300
C	2.85077800	1.51065200	0.19755500
C	2.45075800	2.47602000	-0.87332000
C	-3.34816700	0.44638100	0.41709100
H	-3.22363900	-0.59002100	0.09843900
C	-3.83310300	1.56202700	-0.21425900
H	-4.17393400	1.65683300	-1.24486500
C	-3.77808800	2.59714800	0.74863700
C	-4.24663200	3.99676000	0.46271900
C	0.68114100	-1.10827700	2.70267700
C	2.05654700	-1.10446800	2.44043700
C	2.85552000	-2.09629100	3.01577000
H	3.91437000	-2.12189200	2.75298500
C	2.34464900	-3.05749000	3.90205600
C	0.98340500	-3.00378000	4.22514400
H	0.55145300	-3.71903600	4.93389600
C	0.15998100	-2.05340000	3.63232500
C	-1.24149300	-1.72617700	3.75237700
C	-2.26597900	-2.13930600	4.60985500
H	-2.05677800	-2.92299300	5.34420700
C	-3.51273400	-1.51116000	4.57495700
C	-3.73183400	-0.52361400	3.59207500
H	-4.69383500	-0.00586100	3.51671700
C	-2.73115200	-0.11816100	2.72335300
C	-1.45394100	-0.67715200	2.82669200
C	3.21992600	-4.15729000	4.50338600
C	2.69285600	-5.52045300	4.04342700

H	3.31091700	-6.33687800	4.45578500
H	2.71425100	-5.59779000	2.94266500
H	1.65396600	-5.69166400	4.36853300
C	3.17109000	-4.07492200	6.03187700
H	2.14923600	-4.20152500	6.42362800
H	3.54473100	-3.09915400	6.38560900
H	3.79782000	-4.86411500	6.48195700
C	4.67821200	-4.04195800	4.07001400
H	5.26932300	-4.85199400	4.52802200
H	5.12907300	-3.08705100	4.38958400
H	4.79365000	-4.13198500	2.97653200
C	-4.60419500	-1.77093100	5.60993700
C	-4.30601700	-3.00849600	6.44998200
H	-3.38127600	-2.89364500	7.04035800
H	-4.20704600	-3.91417600	5.82685100
H	-5.12579500	-3.18180200	7.16650300
H	-5.45147700	-0.68438600	7.30329600
H	-4.88986000	0.37939600	5.98278600
H	-3.70465100	-0.40504000	7.05623900
C	-5.96191100	-1.96283800	4.93017100
H	-5.93536500	-2.80051300	4.21218900
H	-6.29014700	-1.05909900	4.39209100
H	-6.73585100	-2.18485200	5.68435500
N	-0.46333700	-0.06314400	-1.12522800
N	-1.15140900	0.49402400	-2.15023900
N	-0.70220100	2.56525500	-0.01753600
N	-0.07502600	3.12876200	2.87457600
N	-1.04696900	2.61955600	3.66931000
C	-1.67721600	-0.46954900	-2.94243200
H	-2.28464600	-0.21380600	-3.80802500

C	-1.29837500	-1.69264000	-2.43014100
H	-1.54328000	-2.67824000	-2.82185600
C	-0.53028300	-1.39010000	-1.29051300
C	0.19007900	-2.33410200	-0.38279300
C	1.14981800	2.75949900	3.32087900
H	2.04637100	3.09883000	2.79974300
C	0.96544200	1.96793400	4.42922500
H	1.73325300	1.47565200	5.02152800
C	-0.43360100	1.90594600	4.62297200
C	-1.14818100	1.14627100	5.70971200
C	-0.96405700	2.80459800	-1.34234200
C	-1.16766400	1.89394900	-2.38468500
C	-1.38338800	2.37528700	-3.67826100
H	-1.49018100	1.64905800	-4.48591800
C	-1.42965500	3.74629300	-3.97469900
C	-1.23587400	4.65455200	-2.92796900
H	-1.25794100	5.73353200	-3.11698700
C	-0.99682400	4.19666100	-1.63636700
C	-0.74665600	4.85375600	-0.37605800
C	-0.67462700	6.19283500	0.01578100
H	-0.82488400	6.97237700	-0.73661100
C	-0.43417700	6.52645100	1.34871100
C	-0.25012000	5.47507500	2.26887700
H	-0.09895100	5.68273200	3.33321800
C	-0.31465900	4.13959000	1.89630100
C	-0.57338000	3.80609500	0.56154000
C	-1.65810900	4.26629900	-5.39424100
C	-0.44538100	5.09404200	-5.83101100
H	-0.58522200	5.47637500	-6.85701800
H	0.47328500	4.48295200	-5.81644200

H	-0.27857000	5.96220700	-5.17326100
C	-2.91415000	5.14243900	-5.41760200
H	-2.82716100	6.00956700	-4.74351200
H	-3.80185200	4.56642300	-5.10606200
H	-3.09778300	5.52955000	-6.43482600
C	-1.84891800	3.13714400	-6.40238900
H	-2.01384800	3.55789500	-7.40794400
H	-2.72597200	2.51279300	-6.16130500
H	-0.96284700	2.48272000	-6.46334700
C	-0.42753300	7.96490400	1.85996200
C	-0.50764400	8.97295300	0.71815600
H	-1.44462800	8.87213600	0.14527100
H	0.33893300	8.87102800	0.01728400
H	-0.48394900	9.99828400	1.12285600
C	-1.64234300	8.16437500	2.77267100
H	-1.65028200	9.18747200	3.18756100
H	-1.65731700	7.45098700	3.61362000
H	-2.58062600	8.01644400	2.21113400
C	0.85836600	8.23483700	2.64571300
H	1.75003800	8.07492000	2.01512800
H	0.94904300	7.58685000	3.53191900
H	0.87624000	9.27864500	3.00317700
Br	-2.57297800	4.89199300	4.98473700
Br	-5.21134300	2.88093700	4.11349600
Ni	-2.95111200	3.06486500	3.57752500
H	-1.95896600	0.57454100	5.22052800
C	-1.79224900	2.03636900	6.77029200
C	-0.20328500	0.16281500	6.38878200
C	-3.86066900	5.06901000	1.46450600
C	-5.76195600	3.98359000	0.25848900

H	-3.77008000	4.24772800	-0.50909600
H	0.58168100	-1.72759800	0.44163100
C	1.38395600	-2.95616600	-1.09641400
C	-0.73899900	-3.37581700	0.22168000
C	2.56154900	1.82685400	-2.25025300
C	3.22235700	3.78283500	-0.79310600
H	1.38969200	2.70072800	-0.70148300
H	2.06667400	-2.17625100	-1.47493100
H	1.06924800	-3.57795400	-1.95308200
H	1.95157000	-3.59775900	-0.40154300
H	-1.56658300	-2.90007300	0.77612600
H	-0.18628200	-4.01179700	0.93381500
H	-1.17452400	-4.03446100	-0.55045900
H	3.61237400	1.59744200	-2.50118000
H	2.15990400	2.50209400	-3.02537000
H	1.98869500	0.88466800	-2.29046800
H	4.29912600	3.63929700	-0.99270500
H	3.11653000	4.25804200	0.19699700
H	2.83810400	4.49322200	-1.54289500
H	-6.25955400	3.68691100	1.19736700
H	-6.07338500	3.28287700	-0.53282500
H	-6.11862100	4.98945200	-0.01617400
H	-4.46286600	5.00507100	2.38376400
H	-4.03328200	6.06380100	1.02035100
H	-2.80064700	5.01991100	1.75344800
H	0.34477800	-0.47315000	5.67563900
H	-0.77293200	-0.50094400	7.06014600
H	0.53611200	0.69774200	7.01143100
H	-2.64914500	2.60382800	6.38276600
H	-1.06768400	2.76542900	7.17149900

H	-2.14589700	1.40857600	7.60526100

4^{TS2} (G = -11192.77864 Hartree)

Atomic Number	Coordinates (Angstroms) X	Y	Z
Ni	0.28488900	0.19905200	0.42360500
Br	1.11842300	-0.76438100	-2.55552500
Ni	-0.71055800	-1.46930000	-1.46869900
N	-1.86500900	0.11717500	0.58392600
N	0.34188300	1.53081700	2.01858300
N	-0.53107900	2.55732400	2.04033200
C	-0.15232500	3.49357000	2.94615400
C	1.02410600	3.06671000	3.51280000
C	1.30286100	1.82472000	2.90818400
Br	-2.23922700	-1.98629700	-3.07685900
N	2.22507400	0.31476400	0.27482700
N	0.59553500	-1.44201200	1.80492600
N	0.26613700	2.04561700	-1.01437800
N	-1.13598900	-2.94040300	-0.36560500
C	2.95428100	1.32101800	-0.28637000
C	3.06345800	-0.75099000	0.37413100
C	-3.00676400	-0.64969900	0.33306000
C	-2.35334400	1.39467500	0.79901400
C	2.56260400	2.62444900	-0.62260100
C	4.29928300	0.92481800	-0.52559600
C	2.83789700	-2.00882800	0.95742000
C	4.35669800	-0.46167500	-0.14234500

C	-0.72167200	2.70109800	-1.62437300
N	1.18489600	2.99299200	-0.65495200
C	-0.13059400	-1.83616900	2.86512800
N	1.68525600	-2.25790500	1.76201300
C	-0.46896600	-4.09238800	-0.24515200
N	-2.27848100	-3.04557800	0.32457000
C	-3.27217600	-2.03629000	0.23482900
C	-4.17354600	0.17252100	0.25601100
C	-1.74036100	2.56288600	1.30310800
C	-3.74070900	1.50721300	0.52972600
C	3.53952800	3.52139100	-1.05532700
C	5.25380100	1.83754600	-0.96769000
C	3.84244500	-2.97069200	0.86971600
C	5.36142700	-1.42762100	-0.17528300
C	-1.77458300	1.99745700	-2.42372600
C	-0.50295900	4.10026300	-1.55831100
C	0.71917000	4.23651100	-0.94634500
C	-1.37431700	-1.16686000	3.37949900
C	0.49543300	-2.93002800	3.49651100
C	1.64610300	-3.15330200	2.78230500
C	2.49215400	0.97430300	3.24461000
C	0.80974900	-4.34066500	-0.98054600
C	-1.22212400	-4.96997500	0.55682000
C	-2.36659300	-4.27076500	0.88946600
C	-4.56806700	-2.50609800	0.00917300
C	-5.45617700	-0.30873700	0.01982900
C	-2.39942600	3.78108600	1.16978700
C	-4.43328700	2.71833100	0.53028100
C	4.89310900	3.16477900	-1.20496100
H	3.22643400	4.52635200	-1.34089700

H	6.28758300	1.50488000	-1.11480700
C	5.10705400	-2.71207600	0.30457100
H	3.64686200	-3.96800300	1.27148400
H	6.33935000	-1.15997300	-0.58417600
H	-1.96781400	1.01928300	-1.94334400
H	-1.73548600	-0.50372600	2.58534500
H	0.15041900	-3.46234100	4.38116900
H	2.46673500	-3.84570200	2.95381500
H	2.42691300	0.05435600	2.65316500
H	1.60737500	3.56775900	4.28330300
H	-0.76887500	4.36492200	3.15040200
H	1.35474600	-3.37647300	-1.03054600
C	-5.68529600	-1.67510300	-0.11390800
C	-3.73186400	3.89998200	0.74366100
H	-1.83535000	4.68964900	1.39909800
H	-5.49432100	2.72231900	0.26510200
C	5.95403800	4.16991200	-1.65689600
C	6.15640400	-3.82445700	0.29206100
C	-7.08785600	-2.20486000	-0.39862500
C	-4.29004800	5.28862000	0.45351300
C	7.04067600	4.27074900	-0.58180100
C	6.57896200	3.70152000	-2.97437000
C	5.37783800	5.56562000	-1.87680800
C	5.60167200	-5.06611800	-0.41096600
C	6.53320800	-4.18464700	1.73304900
C	7.42612500	-3.39656300	-0.43799200
C	-8.03656400	-1.76695500	0.72086200
C	-7.57312900	-1.63742200	-1.73592000
C	-7.12106500	-3.72747800	-0.48421600
C	-3.53536700	5.83258600	-0.76919600

C	-4.10318900	6.21934100	1.65266300
C	-5.77506300	5.24305700	0.11033400
H	6.61332600	4.60969500	0.37730900
H	7.82173900	4.99096900	-0.88279300
H	7.53260700	3.30126300	-0.40167200
H	5.81365600	3.61855700	-3.76436400
H	7.34825300	4.41618900	-3.31591300
H	7.06177900	2.71602600	-2.87553300
H	4.92525600	5.97370000	-0.95707900
H	6.17999800	6.25731700	-2.18367300
H	4.61441300	5.57694800	-2.67300200
H	5.27672200	-4.82845000	-1.43852300
H	6.37173700	-5.85478900	-0.46831000
H	4.73966900	-5.49624600	0.12503900
H	6.94267500	-3.30758200	2.26219200
H	7.29685200	-4.98205200	1.74979500
H	5.66413800	-4.54494500	2.30838000
H	7.22253200	-3.12638900	-1.48767200
H	8.15413100	-4.22480300	-0.44141700
H	7.91267200	-2.53598000	0.05011100
H	-7.70473800	-2.16223200	1.69602200
H	-9.05822400	-2.13958600	0.53092300
H	-8.09515600	-0.66993800	0.80820400
H	-6.89585700	-1.93109500	-2.55535000
H	-8.58414300	-2.01205700	-1.97340500
H	-7.61959300	-0.53633100	-1.72216900
H	-6.79887300	-4.19950500	0.45965900
H	-8.14957000	-4.06884400	-0.68785900
H	-6.48118600	-4.10825800	-1.29780900
H	-3.73057000	5.19059400	-1.64787200

H	-3.86589100	6.85651500	-1.01678800
H	-2.44152600	5.84216300	-0.61760400
H	-4.61940400	5.82108500	2.54242500
H	-4.51928500	7.21819500	1.43539500
H	-3.04233700	6.36202100	1.91498700
H	-5.97270800	4.61466800	-0.77411200
H	-6.13950300	6.25772800	-0.12120500
H	-6.37690800	4.85547300	0.94995800
H	-1.17190800	4.89329500	-1.89332300
H	1.30843100	5.12314500	-0.71925000
H	-4.67797900	-3.58493900	-0.11842100
H	-6.28319500	0.40821200	-0.03840600
H	-0.95690500	-5.98019900	0.86049300
H	-3.22471600	-4.53969500	1.50337900
C	1.66484100	-5.37311800	-0.26541800
H	1.20672400	-6.37622300	-0.32842100
H	2.65801200	-5.43786600	-0.73622600
H	1.80020000	-5.13257600	0.80287500
C	0.51039100	-4.78149000	-2.41287800
H	0.03953400	-3.97245000	-2.99289400
H	1.43973700	-5.07833500	-2.92490900
H	-0.17547100	-5.64643600	-2.41628800
C	-1.21181800	1.72009500	-3.82282900
H	-1.84278100	0.98194600	-4.34438300
H	-1.18367700	2.64928000	-4.41791800
H	-0.19356500	1.30496100	-3.77943600
C	-3.07444000	2.77551700	-2.55609800
H	-2.96492500	3.60190600	-3.28214100
H	-3.87209400	2.11374300	-2.93182800
H	-3.41333500	3.20692200	-1.60576000

C	3.79674200	1.69761200	2.88938100
H	4.14950300	2.31353600	3.73536300
H	4.59133300	0.97132600	2.65104200
H	3.68762800	2.35935700	2.01589000
C	2.49884000	0.57556200	4.71778900
H	3.40485700	-0.01259400	4.93936500
H	2.51200200	1.46042400	5.37797300
H	1.62664800	-0.03890800	4.98836900
C	-1.08591100	-0.29896500	4.60216700
H	-2.03046100	0.11421600	4.99370600
H	-0.62055100	-0.88672200	5.41440200
H	-0.42578200	0.54958200	4.36620400
C	-2.46724000	-2.17960400	3.72434600
H	-2.35622900	-2.55945200	4.75579000
H	-3.46298100	-1.71234000	3.65066500
H	-2.45652200	-3.05060200	3.05328900

$Cz^{tBu}(Pyr^{iPr})_2NiBr$ (G = -5596.42195 Hartree)

Atomic Number	Coordinates (Angstroms) X	Y	Z
Br	2.43165500	-0.26611300	2.25767800
Ni	1.47948400	-0.10102600	0.11428000
N	1.72381900	1.90870500	-0.27689400
N	0.72643500	2.80060900	-0.50341800
N	-0.34612800	0.01958600	-0.40154100
N	0.35852900	-2.88047800	-0.49536700

N	1.45623000	-2.09688400	-0.38641000
C	1.24749400	4.00899400	-0.82842900
H	0.60395900	4.85044000	-1.07385700
C	2.61947900	3.90387300	-0.79501000
H	3.34607900	4.68635300	-1.00599400
C	2.87471900	2.56507000	-0.44081400
C	4.20717700	1.91730200	-0.22599200
H	4.02239700	0.83351500	-0.10295100
C	5.12287500	2.13579900	-1.42268000
H	4.67449200	1.76091100	-2.35750100
H	6.08713700	1.62267400	-1.27402500
H	5.34297900	3.20832300	-1.56370200
C	4.83633500	2.42226300	1.06780400
H	4.18681600	2.19963200	1.92896400
H	5.00564300	3.51261200	1.02332600
H	5.80992900	1.93524100	1.24026900
C	0.72049300	-4.14769600	-0.81091100
H	-0.02427700	-4.92384900	-0.96913000
C	2.09540800	-4.18731100	-0.89822200
H	2.71380500	-5.04982300	-1.13566100
C	2.51579200	-2.87278200	-0.62409400
C	3.89820600	-2.29637900	-0.63805800
H	3.99992300	-1.70325500	0.29329200
C	4.96045400	-3.38127800	-0.64558600
H	4.84759400	-4.06950500	0.20689500
H	5.96695000	-2.93848700	-0.58974400
H	4.91522600	-3.97699900	-1.57454500
C	4.06323000	-1.36035000	-1.83302000
H	3.92805600	-1.90893200	-2.78119700

H	5.06726500	-0.90496400	-1.84147500
H	3.32284100	-0.54002200	-1.82096700
C	-1.09070000	1.16082600	-0.32005600
C	-0.65107700	2.48815500	-0.34334700
C	-1.59837200	3.50570600	-0.21670900
H	-1.24969800	4.53917700	-0.18941100
C	-2.97439900	3.24564900	-0.10072400
C	-3.40425600	1.91343300	-0.09194200
H	-4.46932600	1.67567800	0.00558800
C	-2.47791600	0.87985400	-0.18837300
C	-2.56746100	-0.56361300	-0.17083900
C	-3.62219600	-1.46777600	-0.03931000
H	-4.64205800	-1.08650000	0.05738400
C	-3.36343400	-2.83911200	-0.01339800
C	-2.02845300	-3.27347200	-0.13963100
H	-1.82202200	-4.34640400	-0.09560800
C	-0.96256600	-2.39397200	-0.30166300
C	-1.22977300	-1.01714300	-0.29998700
C	-4.00435400	4.36929400	0.03273700
C	-4.74553900	4.22443300	1.36545200
H	-5.49449800	5.02709600	1.48203300
H	-4.04379500	4.28526100	2.21426200
H	-5.27585800	3.26154900	1.44108500
C	-5.00532000	4.28061000	-1.12332000
H	-5.54558700	3.32034900	-1.13092400
H	-4.49412600	4.38281800	-2.09554500
H	-5.75811900	5.08444600	-1.04647700
C	-3.36352400	5.75359600	-0.00337900
H	-4.14241900	6.52810500	0.09213400

H	-2.83317500	5.93824100	-0.95305300
H	-2.65219500	5.90184700	0.82662100
C	-4.46285100	-3.88756500	0.16245400
C	-5.84460100	-3.24988500	0.27076900
H	-6.10369300	-2.67353300	-0.63313200
H	-5.92048800	-2.57796400	1.14180700
H	-6.60992700	-4.03424700	0.39276500
C	-4.47179200	-4.83425300	-1.04182200
H	-5.26897900	-5.59021300	-0.93410400
H	-3.51851400	-5.37612600	-1.15426200
H	-4.65224600	-4.27871200	-1.97756900
C	-4.20721400	-4.68770200	1.44356800
H	-4.19650600	-4.02486700	2.32502700
H	-3.24272700	-5.22061300	1.41769000
H	-4.99841900	-5.44295600	1.59273500

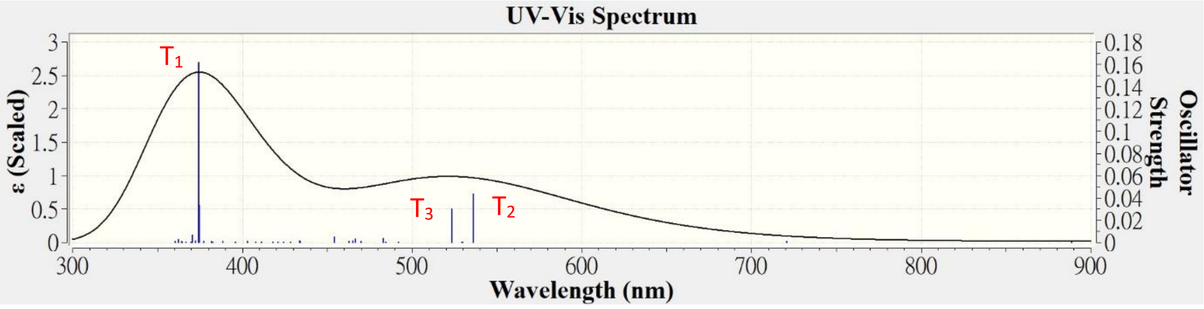

Figure S23. Simulated UV-Vis spectra for **3**$^{\mathbf{Int}}$.

Table S5. Donor and acceptor orbitals of **3**$^{\mathbf{Int}}$ for the excited states responsible for the absorption spectra in Figure S19.

state	λ (nm)	f_{calc} [a]	Nature[b]			contribution
T_1	374.30	0.1613	HOMO-3 (α)	→	LUMO+1 (α)	32%
			HOMO-2 (α)	→	LUMO (α)	52%
			HOMO-1 (α)	→	LUMO (α)	25%
			HOMO-3 (β)	→	LUMO+2 (β)	51%
			HOMO-1 (β)	→	LUMO+2 (β)	13%
T_2	523.41	0.0299	HOMO-14 (β)	→	LUMO+2 (β)	13%
			HOMO-3 (β)	→	LUMO+2 (β)	28%
			HOMO-2 (β)	→	LUMO+1 (β)	86%
T_3	536.10	0.0431	HOMO-14 (β)	→	LUMO (β)	26%
			HOMO-4 (β)	→	LUMO (β)	13%
			HOMO-3 (β)	→	LUMO (β)	81%
			HOMO-1 (β)	→	LUMO (β)	18%

[a] f_{calc} = oscillator strength. [b] α and ß represent α-spin and β-spin of electron.

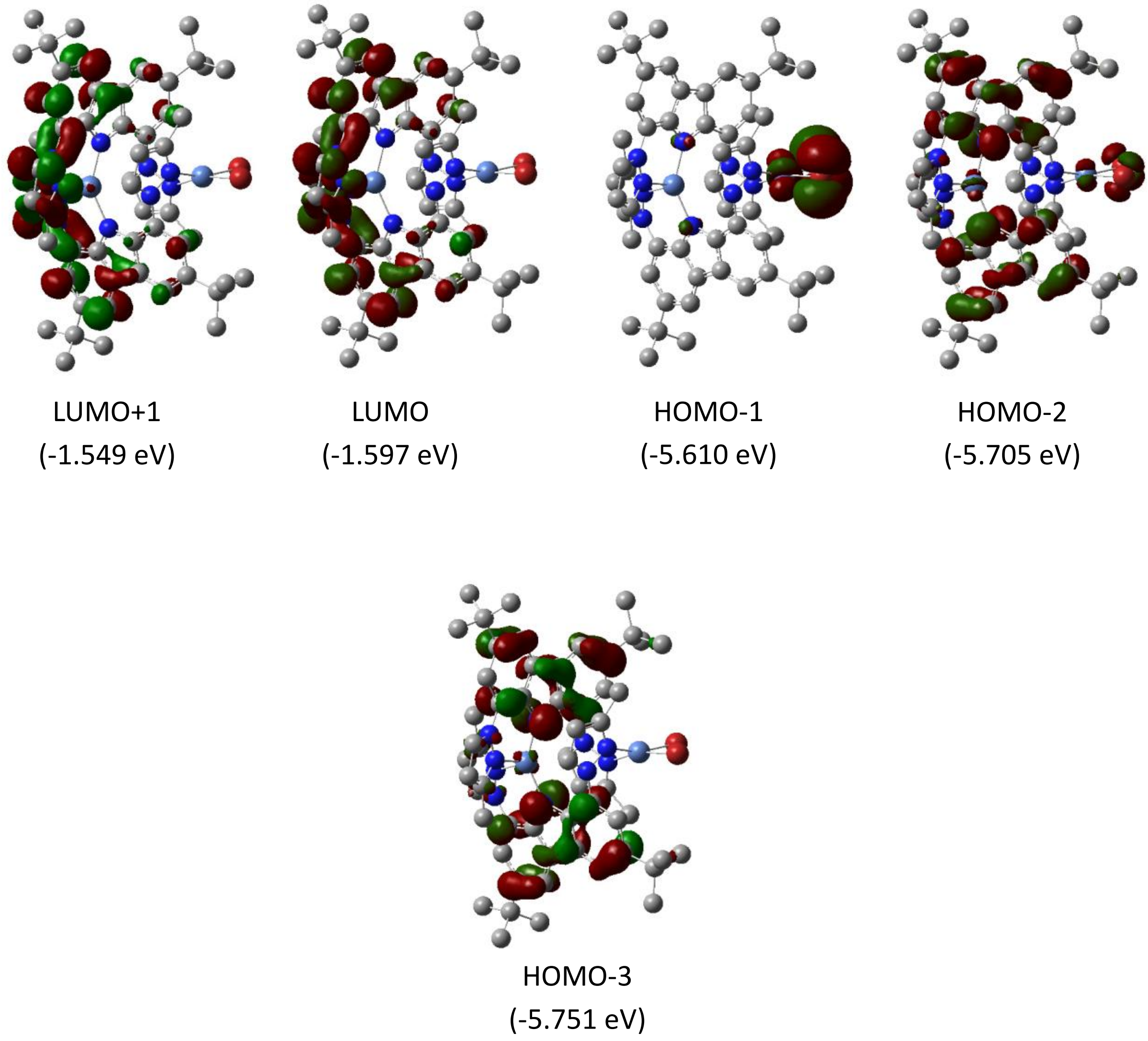

Figure S24. Selected molecular orbitals of $\mathbf{3^{Int}}$ (α-spin). Isovalue = 0.04.

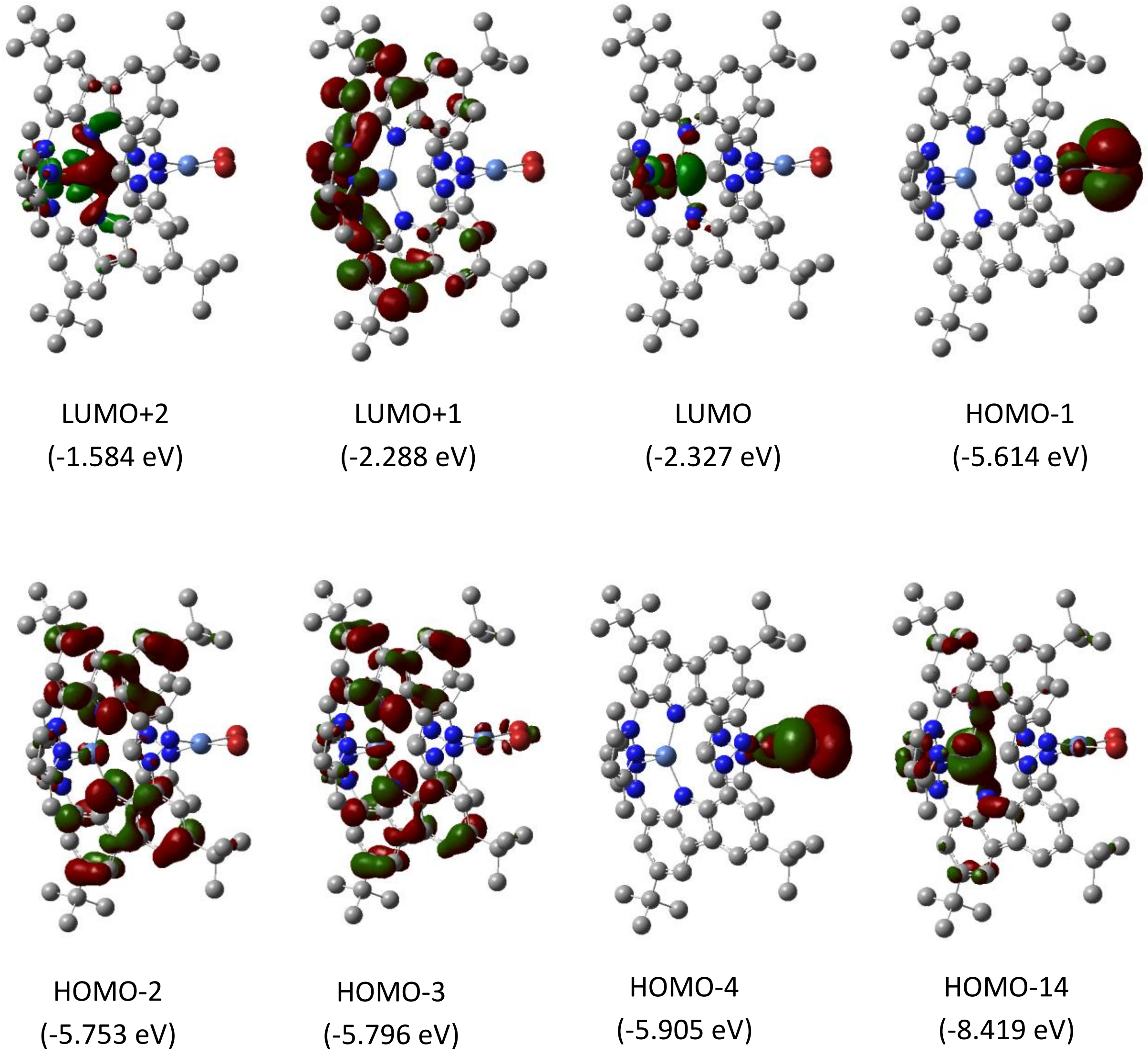

Figure S25. Selected molecular orbitals of $\mathbf{3^{Int}}$ (β-spin). Isovalue = 0.04.

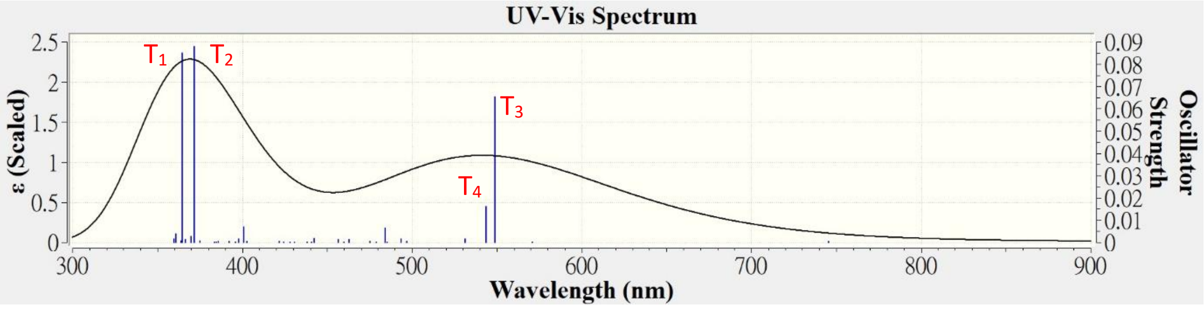

Figure S26. Simulated UV-Vis spectra for $\mathbf{4^{Int}}$.

Table S6. Donor and acceptor orbitals of $\mathbf{4^{Int}}$ for the excited states responsible for the absorption spectra in Figure S22.

state	λ (nm)	f_{calc}[a]	Nature[b]			contribution
T_1	364.70	0.0848	HOMO-3 (α)	→	LUMO (α)	16%
			HOMO-3 (α)	→	LUMO+1 (α)	15%
			HOMO-3 (α)	→	LUMO+2 (α)	25%
			HOMO-2 (α)	→	LUMO (α)	24%
			HOMO-2 (α)	→	LUMO+1 (α)	17%
			HOMO-2 (α)	→	LUMO+2 (α)	41%
			HOMO-1 (α)	→	LUMO (α)	11%
			HOMO-1 (α)	→	LUMO+2 (α)	15%
			HOMO (α)	→	LUMO+2 (α)	13%
			HOMO-5 (β)	→	LUMO+4 (β)	18%
			HOMO-4 (β)	→	LUMO+2 (β)	15%
			HOMO-3 (β)	→	LUMO+4 (β)	38%
			HOMO-2 (β)	→	LUMO+2 (β)	20%
T_2	371.74	0.0877	HOMO-2 (α)	→	LUMO (α)	49%
			HOMO-1 (α)	→	LUMO (α)	16%
			HOMO-4 (β)	→	LUMO+2 (β)	11%
			HOMO-3 (β)	→	LUMO+3 (β)	12%
			HOMO-2 (β)	→	LUMO+3 (β)	39%
T_3	549.05	0.0653	HOMO-4 (β)	→	LUMO (β)	15%
			HOMO-2 (β)	→	LUMO (β)	86%
			HOMO-2 (β)	→	LUMO+1 (β)	14%
T_4	543.84	0.0159	HOMO-20 (β)	→	LUMO+1 (β)	12%
			HOMO-5 (β)	→	LUMO (β)	14%
			HOMO-5 (β)	→	LUMO+1 (β)	30%
			HOMO-3 (β)	→	LUMO (β)	31%
			HOMO-3 (β)	→	LUMO+1 (β)	63%
			HOMO-2 (β)	→	LUMO (β)	12%

[a] f_{calc} = oscillator strength. [b] α and ß represent α-spin and β-spin of electron.

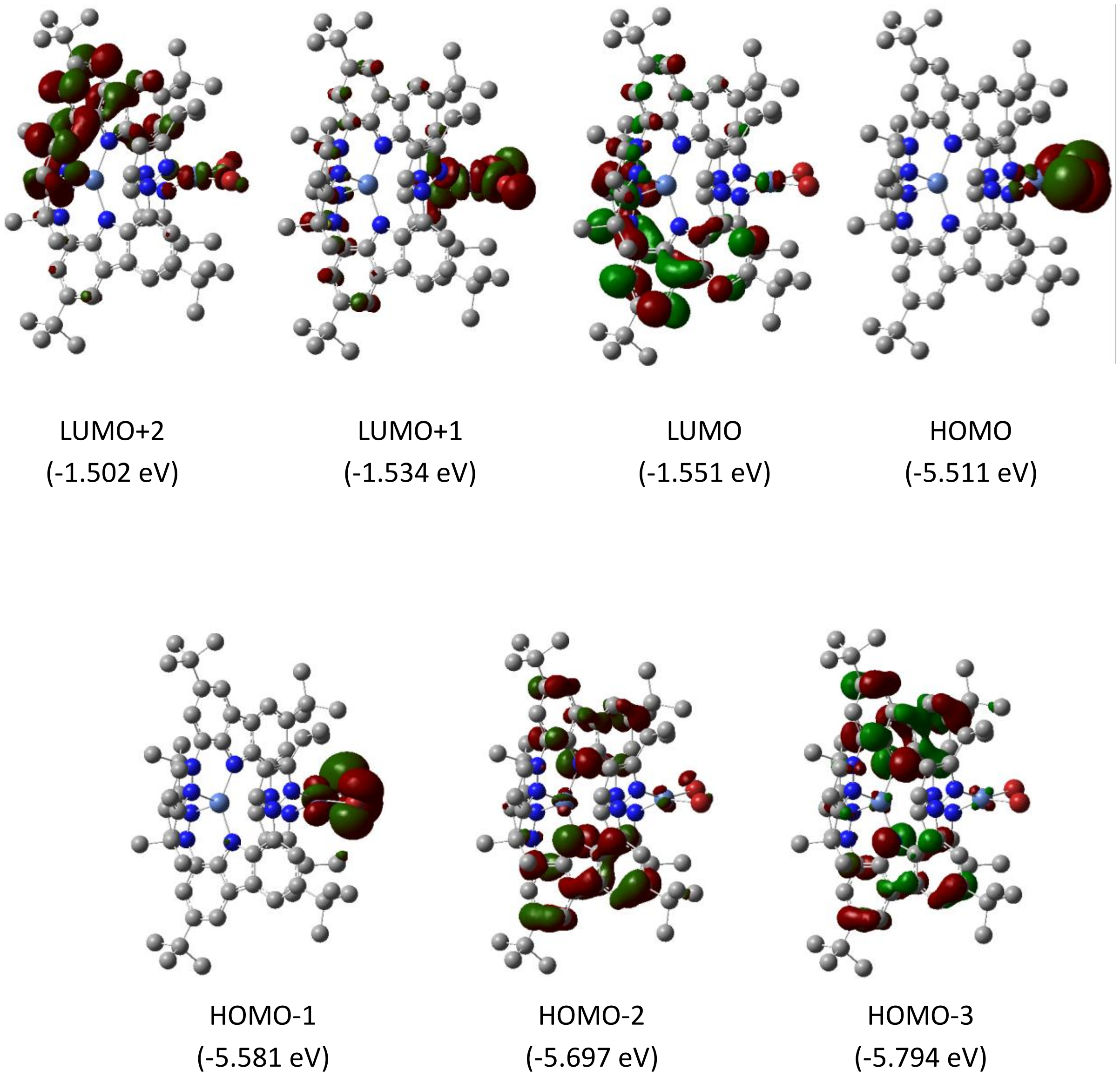

Figure S27. Selected molecular orbitals of $\mathbf{4}^{\mathbf{Int}}$ (α-spin). Isovalue = 0.04.

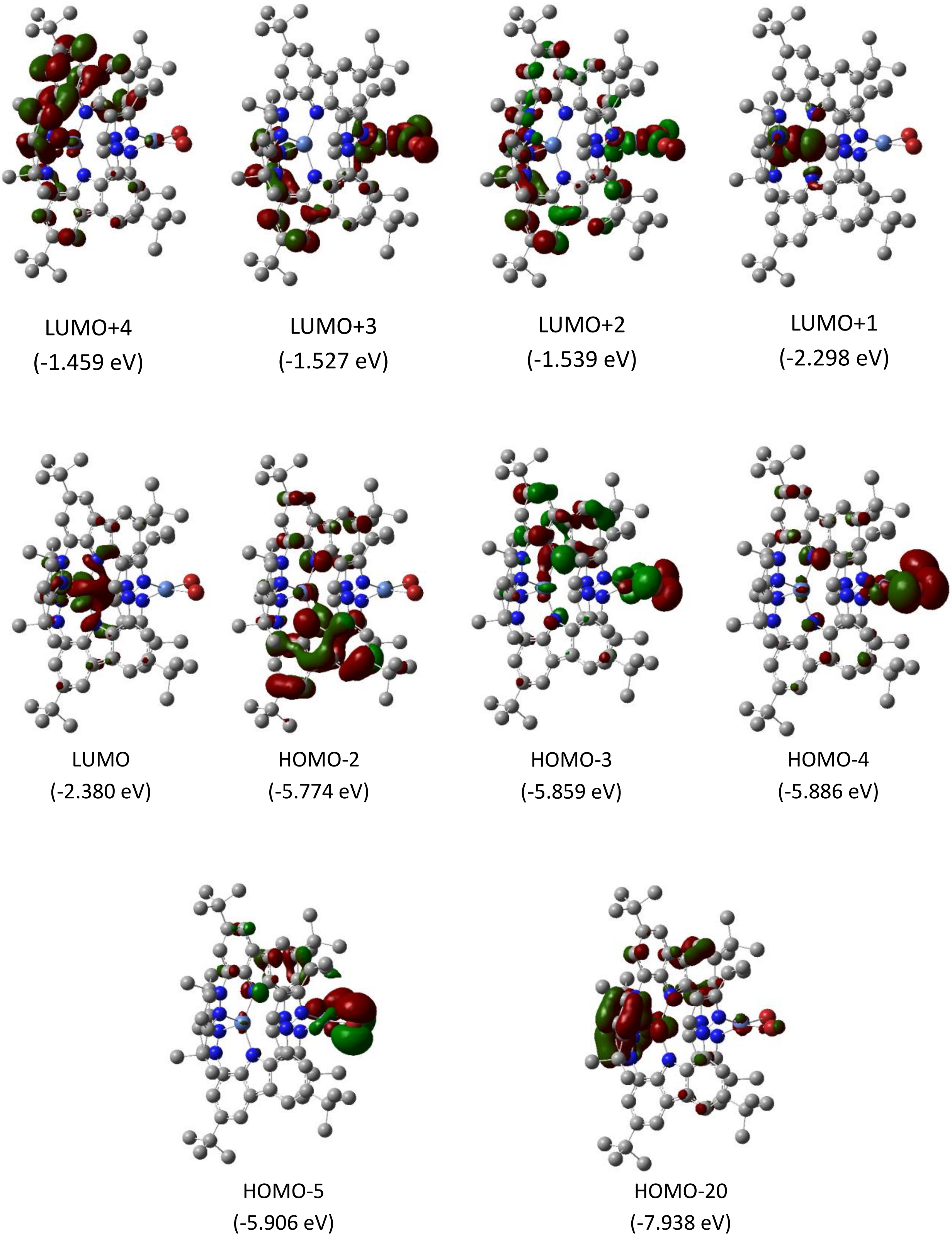

Figure S28. Selected molecular orbitals of $\mathbf{4^{Int}}$ (β-spin). Isovalue = 0.04.

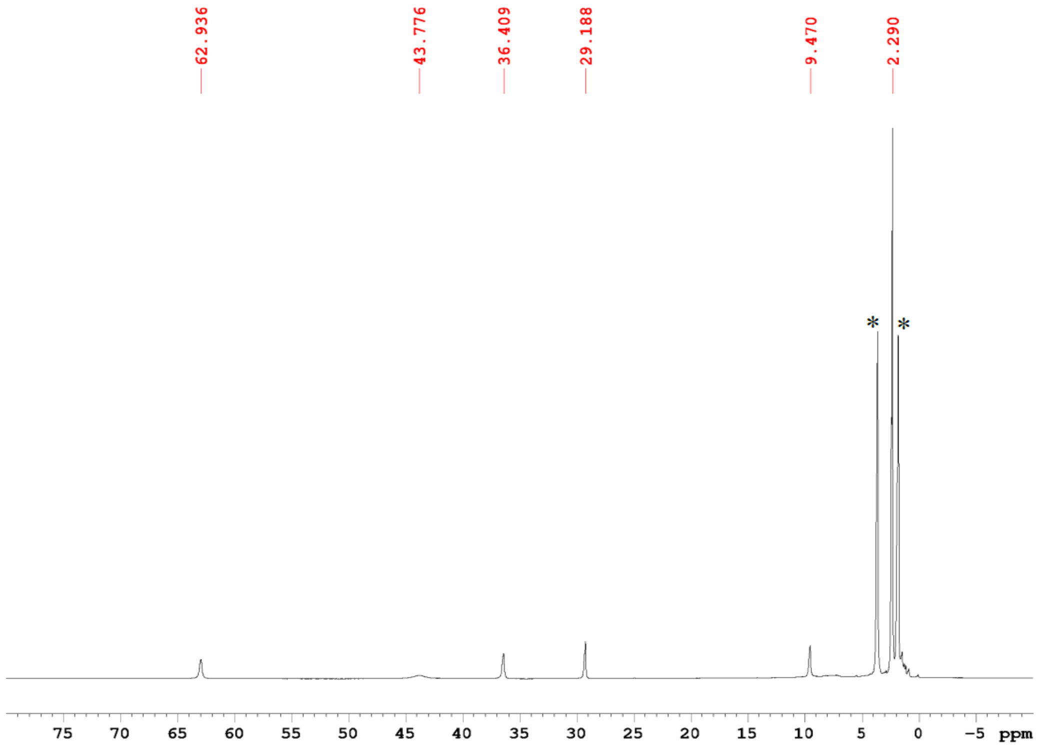

Figure S29. ^{1}H NMR spectrum of **1** in THF-d_8. * solvent residual peaks.

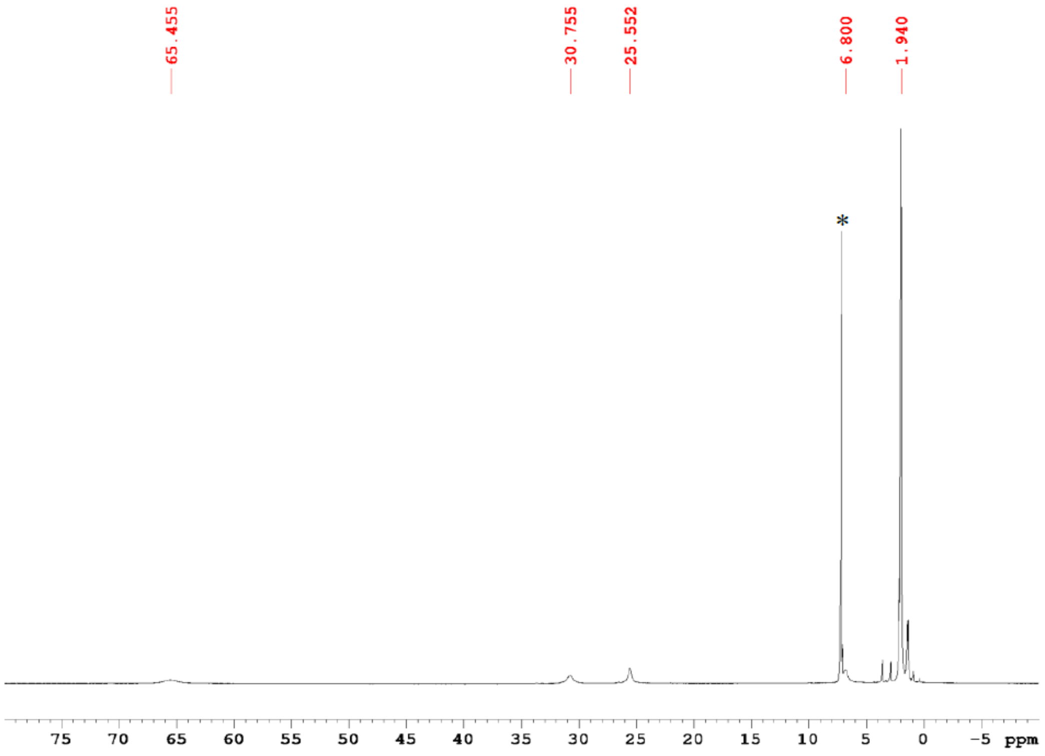

Figure S30. ^{1}H NMR spectrum of **2** in C_6D_6. * solvent residual peaks.

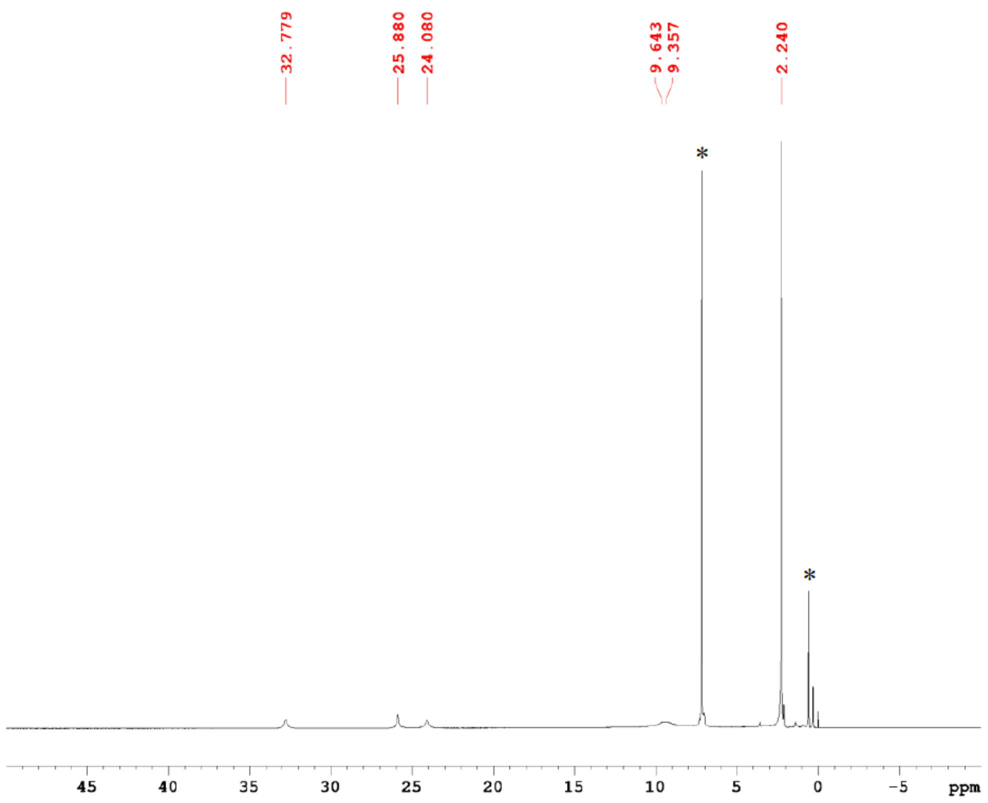

Figure S31. ^{1}H NMR spectrum of **3** in C_6D_6. * solvent residual peaks.

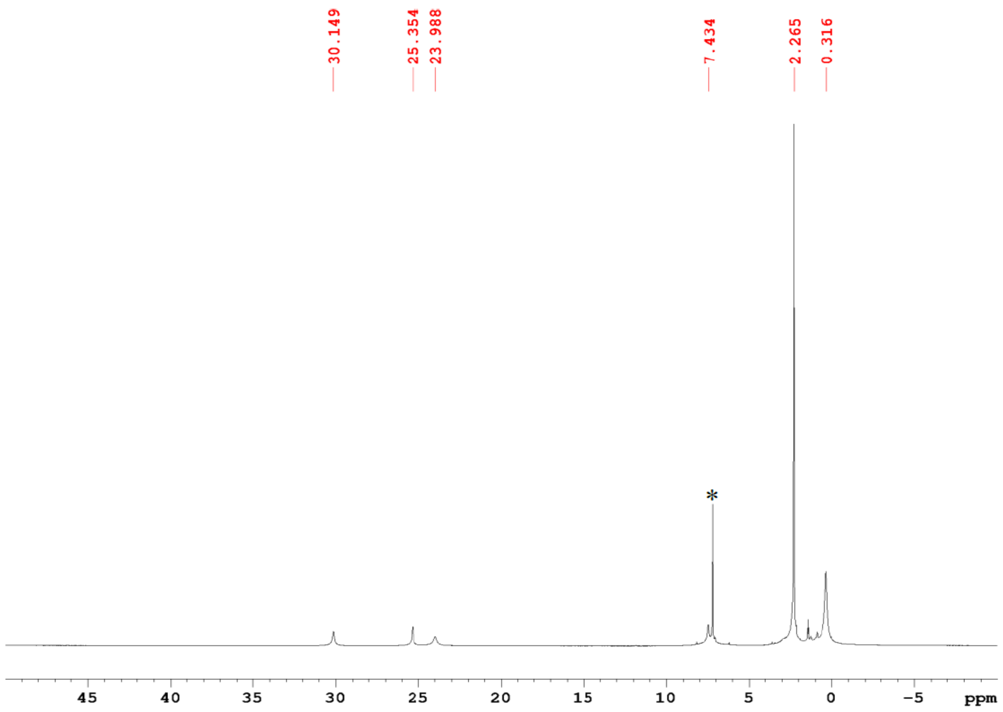

Figure S32. ^{1}H NMR spectrum of **4** in C_6D_6 * solvent residual peaks.

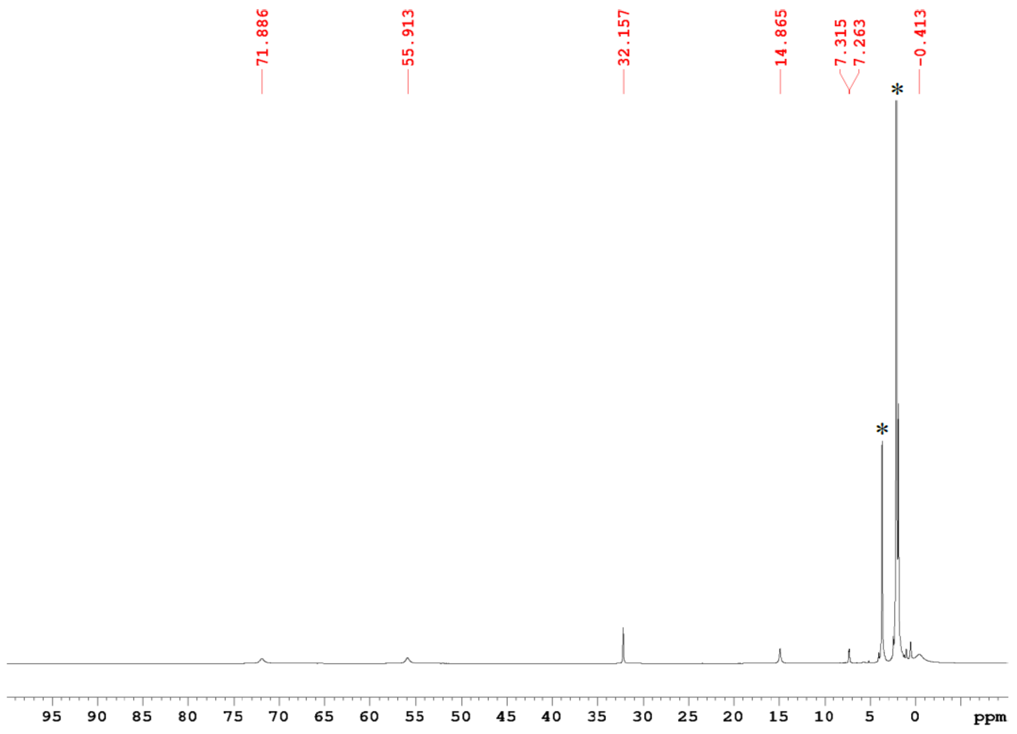

Figure S33. ^{1}H NMR spectrum of $Cz^{tBu}(Pyr^{Me})_2NiBr$ in acetone-d_6. * solvent residual peaks.

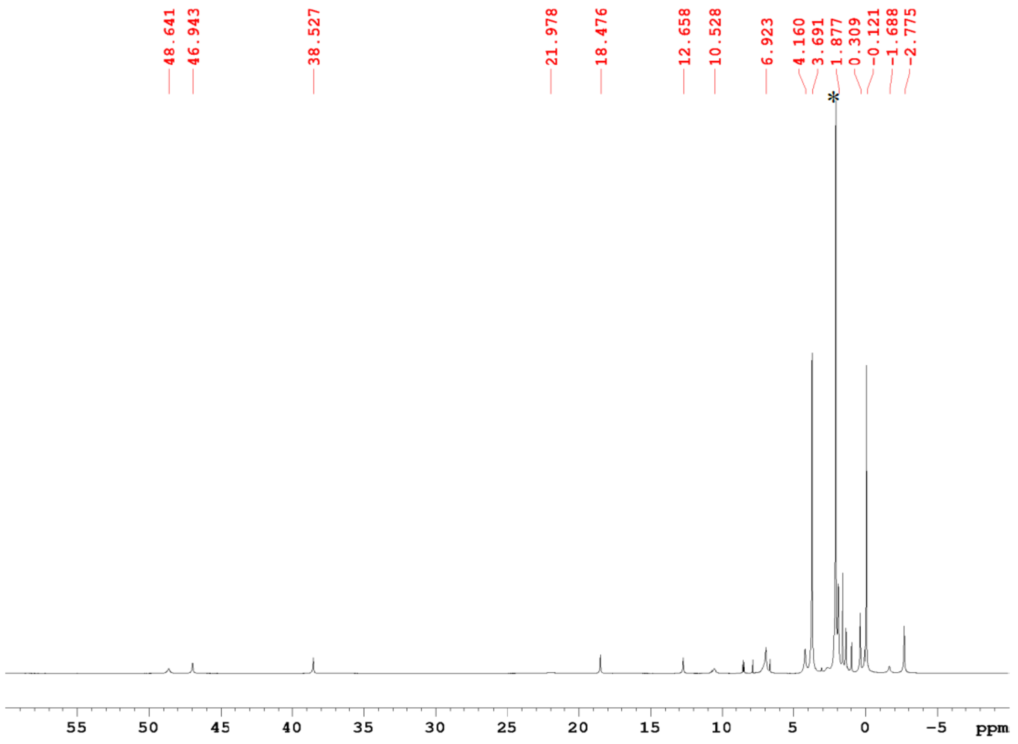

Figure S34. ^{1}H NMR spectrum of $(Cz^{tBu}(Pyr^{iPr})_2)_2Ni$-Ag-$PF_6$ in acetone-d_6. * solvent residual peaks.

References

(1) (a) Apex3 v2016.9-0, APEX5 v2023.9-2; Bruker AXS Inc.: Madison, WI, 2016/2023.

(b) SAINT V8.37A, SAINT V8.40B; Bruker AXS Inc.: Madison, WI, 2016/2019.

(2) Blessing, R. H. An empirical correction for absorption anisotropy. *Acta Crystallogr A* **1995**, *51 (Pt 1)*, 33–38.

(3) Hubschle, C. B.; Sheldrick, G. M.; Dittrich, B. ShelXle: a Qt graphical user interface for SHELXL. *J Appl Crystallogr* **2011**, *44* (Pt 6), 1281–1284.

(4) Sheldrick, G. M. SHELXT - integrated space-group and crystal-structure determination. *Acta Crystallogr A Found Adv* **2015**, *71* (Pt 1), 3–8.

(5) Sheldrick, G. M. A short history of SHELX. *Acta Crystallogr A* **2008**, *64* (Pt 1), 112–122.

(6) Zhao, Y.; Truhlar, D. G. Exploring the Limit of Accuracy of the Global Hybrid Meta Density Functional for Main-Group Thermochemistry, Kinetics, and Noncovalent Interactions. *J Chem Theory Comput* **2008**, *4* (11), 1849–1868.

(7) Grimme, S.; Antony, J.; Ehrlich, S.; Krieg, H. A consistent and accurate ab initio parametrization of density functional dispersion correction (DFT-D) for the 94 elements H-Pu. *J Chem Phys* **2010**, *132* (15), 154104.

(8) Weigend, F.; Ahlrichs, R. Balanced basis sets of split valence, triple zeta valence and quadruple zeta valence quality for H to Rn: Design and assessment of accuracy. *Phys Chem Chem Phys* **2005**, *7* (18), 3297–3305.

(9) Frisch, M. J.; Trucks, G. W.; Schlegel, H. B.; Scuseria, G. E.; Robb, M. A.; Cheeseman, J. R.; Scalmani, G.; Barone, V.; Mennucci, B.; Petersson, G. A.; Nakatsuji, H.; Caricato, M.; Li, X.; Hratchian, H. P.; Izmaylov, A. F.; Bloino, J.; Zheng, G.; Sonnenberg, J. L.; Hada, M.; Ehara, M.; Toyota, K.; Fukuda, R.; Hasegawa, J.; Ishida, M.; Nakajima, T.; Honda, Y.; Kitao, O.; Nakai, H.; Vreven, T.; Montgomery, Jr., J. A.; Peralta, J. E.; Ogliaro, F.; Bearpark, M.; Heyd, J. J.; Brothers, E.; Kudin, K. N.; Staroverov, V. N.; Keith, T.; Kobayashi, R.; Normand, J.; Raghavachari, K.; Rendell, A.; Burant, J. C.; Iyengar, S. S.; Tomasi, J.; Cossi, M.; Rega, N.; Millam, J. M.; Klene, M.; Knox, J. E.; Cross, J. B.; Bakken, V.; Adamo, C.; Jaramillo, J.; Gomperts, R.; Stratmann, R. E.; Yazyev, O.; Austin, A. J.; Cammi, R.; Pomelli, C.; Ochterski, J. W.; Martin, R. L.; Morokuma, K.; Zakrzewski, V. G.; Voth, G. A.; Salvador, P.; Dannenberg, J. J.; Dapprich, S.; Daniels, A. D.; Farkas, O.; Foresman, J. B.; Ortiz, J. V.; Cioslowski, R. L. Martin; K., Morokuma; Ö ., Farkas; J. B., Foresman; D. J., Fox, Gaussian 16; revision C.01; Gaussian, Inc.: Wallingford CT, 2016.
